# Antibiotic management of urinary tract infection in elderly patients in primary care and its association with bloodstream infections and all cause mortality: population based cohort study

**DOI:** 10.1136/bmj.l525

**Published:** 2019-02-27

**Authors:** Myriam Gharbi, Joseph H Drysdale, Hannah Lishman, Rosalind Goudie, Mariam Molokhia, Alan P Johnson, Alison H Holmes, Paul Aylin

**Affiliations:** 1 NIHR Health Protection Research Unit, Healthcare Associated Infections and Antimicrobial Resistance, Imperial College London, London, UK; 2 Department of Primary Care and Public Health, Imperial College London, London, UK; 3 Medical School, St George’s University of London, London, UK; 4 Nuffield Department of Population Health, University of Oxford, Oxford, UK; 5 Department of Primary Care and Public Health Sciences, King’s College, London, UK; 6 Healthcare-Associated Infections and Antimicrobial Resistance Division, National Infection Service, Public Health England, London, UK

## Abstract

**Objective:**

To evaluate the association between antibiotic treatment for urinary tract infection (UTI) and severe adverse outcomes in elderly patients in primary care.

**Design:**

Retrospective population based cohort study.

**Setting:**

Clinical Practice Research Datalink (2007-15) primary care records linked to hospital episode statistics and death records in England.

**Participants:**

157 264 adults aged 65 years or older presenting to a general practitioner with at least one diagnosis of suspected or confirmed lower UTI from November 2007 to May 2015.

**Main outcome measures:**

Bloodstream infection, hospital admission, and all cause mortality within 60 days after the index UTI diagnosis.

**Results:**

Among 312 896 UTI episodes (157 264 unique patients), 7.2% (n=22 534) did not have a record of antibiotics being prescribed and 6.2% (n=19 292) showed a delay in antibiotic prescribing. 1539 episodes of bloodstream infection (0.5%) were recorded within 60 days after the initial UTI. The rate of bloodstream infection was significantly higher among those patients not prescribed an antibiotic (2.9%; n=647) and those recorded as revisiting the general practitioner within seven days of the initial consultation for an antibiotic prescription compared with those given a prescription for an antibiotic at the initial consultation (2.2% *v* 0.2%; P=0.001). After adjustment for covariates, patients were significantly more likely to experience a bloodstream infection in the deferred antibiotics group (adjusted odds ratio 7.12, 95% confidence interval 6.22 to 8.14) and no antibiotics group (8.08, 7.12 to 9.16) compared with the immediate antibiotics group. The number needed to harm (NNH) for occurrence of bloodstream infection was lower (greater risk) for the no antibiotics group (NNH=37) than for the deferred antibiotics group (NNH=51) compared with the immediate antibiotics group. The rate of hospital admissions was about double among cases with no antibiotics (27.0%) and deferred antibiotics (26.8%) compared with those prescribed immediate antibiotics (14.8%; P=0.001). The risk of all cause mortality was significantly higher with deferred antibiotics and no antibiotics than with immediate antibiotics at any time during the 60 days follow-up (adjusted hazard ratio 1.16, 95% confidence interval 1.06 to 1.27 and 2.18, 2.04 to 2.33, respectively). Men older than 85 years were particularly at risk for both bloodstream infection and 60 day all cause mortality.

**Conclusions:**

In elderly patients with a diagnosis of UTI in primary care, no antibiotics and deferred antibiotics were associated with a significant increase in bloodstream infection and all cause mortality compared with immediate antibiotics. In the context of an increase of *Escherichia coli* bloodstream infections in England, early initiation of recommended first line antibiotics for UTI in the older population is advocated.

## Introduction

Urinary tract infection (UTI) is the most common bacterial infection in the older patient population, and *Escherichia coli* is the most common uropathogen in community dwelling people older than 65 years.^1^ The spectrum of UTI ranges from a mild self limiting illness to severe sepsis, with a mortality rate of 20-40%. The incidence of sepsis and its associated mortality increases disproportionately with age, and UTI in men is more likely to be severe.^2^ ^3^ ^4^ Both sexes develop UTI in old age, with a female to male ratio of 2:1 in patients older than 70 years, compared with the overwhelming UTI susceptibility of females in younger populations, with a 50:1 ratio.^5^ The diagnosis of UTI in older patients can be problematic, as these patients are less likely to present with a typical clinical history and localised urinary symptoms compared with younger patients.^6^ The rising incidence of asymptomatic bacteriuria in older adults is also contributing to further diagnostic difficulty (>20% of women aged ≥65 years compared with <5% in younger women), which results in probable over-diagnosis of UTI and unnecessary treatment.^6^ ^7^ ^8^

UTI is the second most common diagnosis for which empirical antibiotics are prescribed in both primary and secondary care, with more than 50% of the antibiotics prescribed for a suspected UTI in older adults being considered unnecessary.^9^ ^10^ ^11^ With the spread of antibiotic resistance and its increasing threat to public health (about 30% of urinary isolates of *E coli* are now resistant to trimethoprim), national guidelines and antimicrobial stewardship programmes have been proposed to combat these challenges.^12^ ^13^ ^14^ ^15^ ^16^ NHS England, for example, released the Quality Premium to incentivise Clinical Commissioning Groups to reduce antibiotic use in primary care.^17^ As a result of these new initiatives, a substantial decrease in antibiotic use has been reported for the first time in England across the whole healthcare system between 2013 and 2017.^16^ ^18^ ^19^ A recent study has also shown a decrease in prescribing of broad spectrum antibiotics for UTI in older people in primary care between 2004 and 2014.^20^ In the meantime, however, increases in the incidence of Gram negative bloodstream infections have been reported, which has led the UK government to announce a plan to reduce healthcare associated Gram negative bloodstream infections in England by 50% by March 2021.^16^

As the pattern of antibiotic use changes in the context of antimicrobial resistance, it is now more important than ever to assess the management and outcome of UTIs. *Clostridium difficile* in elderly people has also been one of the drivers for scrutiny of unnecessary antibiotic use in this population. A decline in antibiotic use may, however, harm vulnerable older populations who are already more likely to develop UTI related complications and bloodstream infection. More evidence is needed about the initial treatment of UTI in primary care, including an assessment of prescribing approaches involving no antibiotics, deferred antibiotics, or immediate antibiotics, and the subsequent clinical outcome. We linked primary care data in England with hospital admissions and mortality data at a patient level, allowing a pragmatic approach to assessing the impact of standard care in the community for a large cohort of older patients with confirmed or suspected UTI on adverse events, including hospital admission, bloodstream infection, and death.

## Methods

We conducted a retrospective population based cohort study in England on patients attending National Health Service general practices submitting data to the UK based Clinical Practice Research Datalink (CPRD) between November 2007 and June 2015.

### Data source

Anonymous medical patient records were extracted from CPRD, the world’s largest primary care electronic health database containing information on a representative national sample. About 7% of English NHS general practices across the country contribute data to this database.^21^ CPRD has been extensively used and validated for pharmacoepidemiological research.

The CPRD database contains a wide ranging set of information, which includes patient sociodemographics, medical diagnoses using the READ classification system, outpatient prescriptions, physiological and laboratory investigations, health behaviours, and referrals to secondary care.^22^ More than 50% of the practices registered with CPRD have agreed to linkage of their records with the corresponding patient hospital records from the hospital episode statistics database, which contains information on all hospital admissions, together with information about the causes of each episode of inpatient care using ICD-10 (international classification of diseases, 10th revision) for the coding of diagnosis, type of admission, procedure performed, length of stay, and discharge status (https://digital.nhs.uk/data-and-information/data-tools-and-services/data-services/hospital-episode-statistics).

We also linked the patients’ primary care data to the death registration data from the Office for National Statistics, which contain date and causes of death, and to the 2010 English index of multiple deprivation data, which contain small area level measures of relative deprivation. For the latter, we obtained a proxy for sociodemographic and socioeconomic status across numerous domains, including housing, employment, income, access to services, education and skills, crime, and living environment, using practice postcode for data linkage (www.cprd.com/dataAccess/linkeddata.asp).

### Population

All patients aged 65 years or older presenting to a general practitioner (GP) with at least one diagnosis of suspected or confirmed lower UTI (recorded using a READ code indicating a clinical test or referral event) in the CPRD database, were included in the study (see supplementary table S1). Patients were excluded if they presented with asymptomatic bacteriuria or had missing data for sex. They were also excluded if they had a diagnosis of a complicated UTI, were admitted to hospital, or died on the same day as their initial UTI diagnosis.

All study participants were registered with a practice for at least 12 continuous months before their first UTI consultation (defined as the index UTI) to capture potential comorbidities and medical history.

**Fig 1:**
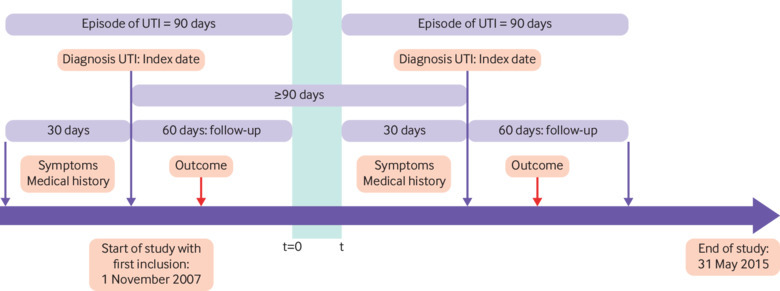
Timeline of study and criteria for differentiating independent episodes of urinary tract infection (UTI)

To distinguish distinct episodes of UTI for the same patient, we used a period of 90 days, comprising 30 days before diagnosis and 60 day follow-up after the index UTI. We considered all the GP consultations within 60 days of the initial UTI diagnosis as being related to the same UTI episode. To identify any relevant medical history for a new episode we used a 30 day buffer period before the index date of the second UTI episode (fig 1).

To ensure data quality we only included practices classified as up to standard (continuous high quality data acceptable for use in research) a year before the start of the study period. Similarly, we only included patient data if an acceptable registration status for use in research, including contiguous follow-up and valid data recording as defined by CPRD, was present at the time of the recruitment and during the follow-up period.^23^ As part of the data management process, we considered UTI observations with the same consultation date to be duplicates. All additional information present in different rows in the database on the same date were grouped together under a same GP consultation.

We further excluded patients for whom primary care medical records were not eligible for data linkage with the hospital episode statistics records and patients without a 60 day follow-up period after the index UTI consultation.

### Exposure

The main exposures were antibiotic prescribing practices after the initial diagnosis of UTI in primary care, defined as immediate antibiotics (patients prescribed an antibiotic during first UTI visit or on same day), deferred antibiotics (patients prescribed an antibiotic within seven days to allow for the natural resolution of the disease,^24^ but not on the day of the initial UTI diagnosis and in the absence of complication or hospital admission, or both), and no antibiotics (patients with no record of having been prescribed an antibiotic by the GP within seven days after the UTI diagnosis or if a complication occurred before antibiotics were prescribed). The name of the antibiotic and duration of treatment were also collected.

The deferred antibiotics group should capture the post-dated prescriptions given to patients on the index date or left behind at the reception to collect on a later date, or the prescriptions for patients who were asked to return if symptoms did not improve.

### Outcome

The primary outcomes of interest were bloodstream infection within 60 days after the initial diagnosis, captured in both hospital episode statistics and CPRD (our definition of bloodstream infection included Read codes and ICD-10 codes related to sepsis, septicaemia, and bacteraemia; see supplementary tables S1 and S2) and all cause mortality within 60 days after the initial UTI diagnosis. The secondary outcomes of interest were hospital admission within 60 days after a UTI diagnosis, length of stay for patients admitted to hospital during a UTI episode, and type of care pathway experienced by the patient.

We classified the patients into three different types of care pathways: single primary care consultation for a UTI without hospital admission or complication related to the UTI within 60 days of the initial diagnosis, multiple primary care consultations for the same UTI episode without hospital admission within 60 days of the initial diagnosis, and single or multiple primary care consultations for the same UTI episode with a hospital admission, regardless of the reason, within 60 days of the UTI diagnosis, and including any UTI related complications (see supplementary tables S1 and S2), bloodstream infection, or death.

### Covariates

A set of covariates was described and used in the models to adjust for potential sources of confounding. Covariates included age (defined as a categorical variable: 65-74, 75-84, and ≥85 years), sex, grouped regions (defined as a categorical variable: North of England and Yorkshire, Midlands and east of England, south of England, and London), area level deprivation (index of multiple deprivation) divided into fifths (first fifth being the least deprived and last fifth the most deprived), year of consultations/diagnoses (financial years from May to April to account for changes in NHS England quality premium guidance),^17^ Charlson comorbidity score (scale from 0 to 12, with higher scores indicating increased risk of death within a year) (see supplementary table S3),^25^ immunosuppression, smoking status, medical history 30 days before the index UTI (indwelling urethral catheter, hospital admission with a discharge date within the 30 days before the index case, antibiotic exposure including short course or prophylactic treatment, presenting symptoms potentially related to UTI), and a history of recurrent UTIs.

We defined a recurrent UTI as the presence of a Read code for recurrent UTI or prophylactic treatment for UTI (trimethoprim or nitrofurantoin prescribed for ≥28 days) or two or more UTIs within 12 months. Although recurrent UTI is usually defined as two or more UTIs within six months or three or more within 12 months,^26^ we adapted this definition to account for the 90 day period between each index case to allow for distinct UTI episodes.

When data were missing on binary covariates, we classified these as absence of the condition.

### Statistical analyses

We first compared patient characteristics and other covariates with different uses of antibiotics. The variables of interest were described using standard measures of central tendency and variability—that is, means and standard deviations for continuous variables and counts and percentages for categorical variables. Comparisons between groups were performed using a range of tests: χ^2^ and Fisher’s exact tests for categorical variables, analysis of variance, and the Kruskal-Wallis for continuous variables. We also compared the rates of bloodstream infection, hospital admission, and all cause mortality as well as the average length of stay for patients admitted to hospital between the three antibiotic groups. The numbers needed to harm (NNH) related to both bloodstream infection and death within 60 days were then calculated. This measure indicated the average number of patients needed to be exposed to no antibiotics and deferred antibiotics to cause harm in an average of one patient who would not otherwise have been harmed if treated with immediate antibiotics.

Differences in the proportion of cases experiencing one of the three care pathways were stratified by antibiotic use, age, and sex and were compared using the χ^2^ test.

The secondary analysis evaluated the predictors of bloodstream infection and death within 60 days after the index UTI. We first constructed the Kaplan-Meier curves for time to death within 60 days and then stratified by antibiotic use and the use of the two first line antibiotics (trimethoprim and nitrofurantoin) at the first visit to the GP. After ensuring the proportional hazard assumptions were met, we compared the curves using the log rank test to assess significance. The main predictor analysed for this study was antibiotic use.

To assess the associations between antibiotic use and bloodstream infection, we performed a multivariable logistic regression analysis, whereas to assess the association between antibiotic use and all cause mortality within 60 days after a UTI diagnosis we used a multivariable Cox regression analysis. The distinct episodes of UTI within the same patient are likely to be correlated with each other, which may affect the apparent relation between antibiotic use and outcome. Not accounting for intracluster correlation and assuming independence between episodes might lead to smaller standard errors and thus narrower confidence intervals for the variable estimates. Therefore we used the robust standard error approach in both logistic and Cox regression models to derive standard errors that allow for the clustering.

A sensitivity analysis was subsequently undertaken to assess the risks of outcomes selected with use of any antibiotic for any duration. We restricted the sensitivity analyses to antibiotic treatment with durations of fewer than 21 days and 28 days to target only curative treatment (as longer duration of antibiotic use was likely to be prescribed as prophylactic treatment).

Statistical analyses were performed using STATA version 12 (STATA Corp, College Station, TX).

### Patient and public involvement

This project was developed within a context of strong patient and public involvement already established within our research team, university, and trust. Two former patients aged 65 and older reviewed the protocol. Their input helped to refine the research question and to improve the protocol considerably. The dissemination plan targets a wide audience, including members of the public, patients, health professionals, and experts in the specialty through various channels available: written communication, events and conferences, networks and social media.

## Results

**Fig 2:**
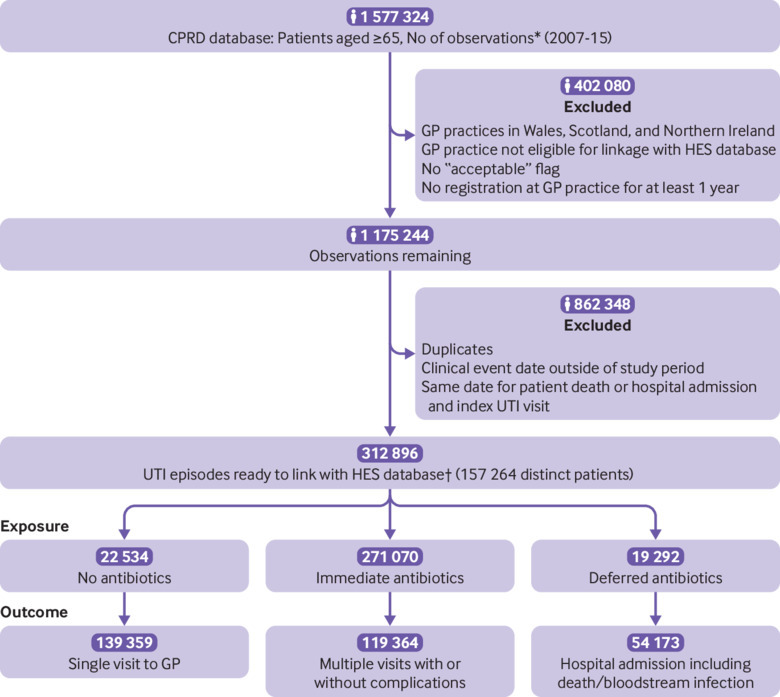
Flowchart of study cohort. *Observations define all general practitioner (GP) contacts (rows in database) in Clinical Research Datalink database (CPRD). †UTI episode contains all GP contacts that define a single event of UTI for a patient. UTI episode includes 30 day period before diagnosis and 60 day follow-up period after diagnosis. HES=hospital episodes statistics

From the CPRD database we extracted 1 577 324 observations relating to a primary care UTI consultation between 1 November 2007 and 31 May 2015 for patients aged 65 and older. After applying our exclusion criteria and removing all duplicates, our analytical sample included 312 896 distinct UTI episodes diagnosed among 157 264 unique patients. An average of two episodes of UTI for each patient were observed in this cohort (fig 2).

The mean age of the study cohort was 76.7 years (SD 9.2 years). At the time of the initial UTI diagnosis, 246 630 (78.8%) participants were women, 40.3% (n=126 215) originated from the south of England, and 28.9% (n=90 464) were from the most deprived areas (index of multiple deprivation fourth and fifth fifths). Overall, 24.2% of the participants (n=75 563) had a Charlson comorbidity index score of 1 or greater, and 22.0% (n=68 967) of the participants had recurrent UTIs (table 1).

**Table 1:** Summary of patients’ characteristics and outcomes related to each episode of urinary tract infection (UTI). Values are numbers (percentages) unless stated otherwise

Characteristics	No with UTI (n=312 896)	Immediate antibiotics (n=271 070)	Deferred antibiotics (n=19 292)	No antibiotics (n=22 534)	P value
Mean (SD) age (years)	76.9 (9.2)	76.3 (9.1)	79.1 (9.2)	79.3 (9.5)	
Age group (years):					
65-74	136 175 (43.5)	122 458 (45.2)	6402 (33.2)	7315 (32.5)	<0.001
75-84	107 485 (34.3)	92 856 (34.3)	6881 (35.7)	7748 (34.4)	
≥85	69 236 (22.1)	55 756 (20.6)	6009 (31.1)	7471 (33.1)	
Sex:					
Women	246 630 (78.8)	217 843 (80.4)	13 657 (70.8)	15 130 (67.1)	<0.001
Men	66 266 (21.2)	53 227 (19.6)	5635 (29.2)	7404 (32.9)	
Region:					
North of England and Yorkshire	65 649 (21.0)	56 744 (20.9)	4178 (21.7)	4727 (21.0)	<0.001
Midlands and east of England	89 337 (28.6)	76 695 (28.3)	5809 (30.1)	6833 (30.3)	
South of England	126 215 (40.3)	110 123 (40.6)	7457 (38.6)	8635 (38.3)	
London	31 695 (10.1)	27 508 (10.1)	1848 (9.6)	2339 (10.4)	
Index of multiple deprivation (fifths):					
1st (least deprived)	77 945 (24.6)	67 081 (24.8)	4668 (24.2)	5196 (23.1)	<0.001
2nd	75 949 (24.3)	66 084 (24.4)	4589 (23.8)	5276 (23.4)	
3rd	69 407 (22.2)	60 277 (22.2)	4193 (21.7)	4937 (21.9)	
4th	51 396 (16.4)	44 239 (16.3)	3229 (16.7)	3928 (17.4)	
5th (most deprived)	39 068 (12.5)	33 279 (12.3)	2603 (13.5)	3186 (14.1)	
Mean (SD) Charlson comorbidity index score	0.36 (0.8)	0.35 (0.7)	0.44 (0.9)	0.44 (0.9)	<0.001
Charlson comorbidity index score ≥1	75 563 (24.2)	63 694 (23.6)	5492 (28.5)	6377 (27.9)	<0.001
Immunosuppression	82 (0.03)	67 (0.02)	8 (0.04)	7 (0.03)	0.348
Renal disease	10 215 (3.3)	8,588 (3.2)	746 (3.9)	881 (3.9)	<0.001
Smoking	12 449 (4.0)	10 798 (4.0)	751 (3.9)	900 (4.0)	0.818
Recurrent UTI	68 967 (22.0)	59 456 (21.9)	6072 (31.5)	3439 (15.3)	<0.001
Indwelling urethral catheter	2627 (0.8)	1933 (0.7)	352 (1.8)	342 (1.5)	<0.001
Hospital admission within 30 days before UTI diagnosis	35 825 (11.4)	22 930 (8.5)	5252 (27.2)	7643 (33.9)	<0.001
Antibiotics exposure 30 days before UTI diagnosis	61 832 (19.8)	49 079 (18.1)	7173 (37.2)	5580 (24.8)	<0.001
Symptoms within 30 days before UTI diagnosis†:	23 502 (7.5)	19 172 (7.1)	2021 (10.5)	2309 (10.2)	
Enuresis	13 (0.01)	10 (76.9)	3 (23.1)	0	<0.001
Offensive urine	50 (0.02)	43 (86)	5 (10)	2 (4)	
Urgency	397 (0.1)	348 (87.7)	23 (5.8)	26 (6.5)	
Malaise	688 (0.2)	527 (76.6)	69 (10.0)	92 (13.4)	
Fatigue	694 (0.2)	607 (87.5)	39 (5.6)	48 (6.9)	
Confusion	1459 (0.5)	895 (61.3)	241 (16.5)	323 (22.1)	
Haematuria	2065 (0.7)	1621 (78.5)	205 (9.9)	239 (11.6)	
Incontinence	2159 (0.7)	1783 (82.6)	194 (9.0)	182 (8.4)	
Micturition frequency	3682 (1.2)	3151 (85.6)	261 (7.1)	270 (7.3)	
Dysuria	4158 (1.3)	3411 (82.0)	398 (9.6)	349 (8.4)	
Pain*	9604 (3.1)	7896 (82.2)	746 (7.8)	962 (10.0)	
Outcome:					
No (%) with bloodstream infection (95% CI)	1539 (0.5; 0.5 to 0.5)	479 (0.2; 0.1 to 0.2)	413 (2.2; 1.9 to 2.4)	647 (2.9; 2.7 to 3.1)	<0.001
No (%) admitted to hospital (95% CI)	51 261 (16.4, 16.2 to 16.5)	40 022 (14.8, 14.6 to 14.9)	5165 (26.8, 26.2 to 27.4)	6074 (27.0, 26.4 to 27.5)	<0.001
Mean (SD) length of stay (days)	7.1 (15.0)	6.3 (14.0)	7.7 (13.2)	12.1 (20.9)	<0.001
No (%) of deaths at 60 days (95% CI)	6193 (2.0, 1.9 to 2.0)	4431 (1.6, 1.6 to 1.7)	545 (2.8, 2.6 to 3.1)	1217 (5.4, 5.1 to 5.7)	<0.001

* Pain in locations linked with UTI excluding dysuria.

† Number of UTI episodes where patients reported having at least one symptom within 30 days before the UTI diagnosis. Patients could report one or more symptoms, so the number of episodes for each symptom aggregated across all symptoms differ from the total of participants who experienced one or more symptoms during an episode. In this section, all but the first column are row %, which differ from the rest of the table.

For 7.2% (n=22 534) of the UTI episodes a record of an antibiotic prescription in primary care was lacking and 6.2% (n=19 292) were related to a delay in antibiotic prescribing. For those participants prescribed antibiotics for a UTI episode, 73.8% (n=200 078) received either trimethoprim (54.7%; n=148 333) or nitrofurantoin (19.1%; n=51 745); cephalosporins or amoxicillin/clavulanic acid were prescribed for 11.5% (n=31 090) and 9.5% (n=25 616) of these episodes, respectively, whereas quinolones were prescribed in 4.4% (n=11 995). Pivmecillinam, which was only included among recommended first line treatments in PHE guidelines in October 2014, was prescribed for 0.4% (n=1084) of these episodes (table 2).27

Patients older than 85 years, living in a deprived area, with a high Charlson comorbidity index score, were mainly managed using either deferred antibiotics or a no antibiotics approach, whereas patients aged between 65 and 74 years were mainly prescribed immediate antibiotics. The female:male ratio was also much higher in the immediate antibiotics group compared with the other groups. A course of antibiotics was more often prescribed at the first visit to the GP or with a delay in patients experiencing recurrent UTIs. Patients who were prescribed antibiotics or were discharged to the hospital within 30 days before the index UTI event were more often prescribed deferred antibiotics or no antibiotics (table 1).

Overall, 7.5% (n=23 502) of the UTI episodes involved at least one of a range of specific or non-specific signs or symptoms within 30 days before the index UTI. Pain, dysuria, micturition frequency, incontinence, and haematuria were the five most frequent symptoms encountered 30 days before a UTI was diagnosed, and 90.8% (19  666/21 668) of the participants with these symptoms recorded were prescribed antibiotics. This proportion was lower for participants with non-specific signs such as confusion (77.9%; 136/1459) and malaise (86.6%; 596/688) (table 1).

**Table 2:** Distribution of antibiotics prescriptions among participants prescribed immediate treatment during their index visit for a urinary tract infection (UTI)

Antibiotics	No (%) (n=271 070)
Trimethoprim	148 333 (54.7)
Nitrofurantoin	51 745 (19.1)
Cephalosporins	31 090 (11.5)
Amoxicillin/clavulanic acid	25 616 (9.4)
Quinolones	11 995 (4.4)
Pivmecillinam	1084 (0.4)
Macrolides	747 (0.3)
Penicillinase resistant penicillins	323 (0.1)
Benzylpenicillin and phenoxymethylpenic	70 (0.03)
Aminoglycosides	27 (0.01)
Clindamycin	3 (<0.01)
Carbapenems	3 (<0.01)
Polymyxin	1 (<0.01)

Overall, 1539 episodes of bloodstream infection (0.5% of total number of UTIs) were recorded in the CPRD or hospital episode statistics, or both within 60 days after a diagnosis of UTI in older people between 2007 and 2015. The rate of bloodstream infection significantly increased when patients were not prescribed antibiotics for their UTI (2.9% *v* 0.2% for immediate antibiotics and 2.2% for deferred antibiotics, P<0.001). After adjusting for covariates, participants in the deferred antibiotics and no antibiotics groups were significantly more likely to experience a bloodstream infection within 60 days compared with participants in the immediate antibiotics group (adjusted odd ratio 7.12, 95% confidence interval 6.22 to 8.14 and 8.08, 7.12 to 9.16, respectively) (table 3).

**Table 3:** Multivariable logistic regression analysis for bloodstream infection 60 days after diagnosis of urinary tract infection (UTI)

Variables	Unadjusted odds ratio (95% CI)*	P value	Adjusted† odds ratio (95% CI)*	P value
Antibiotic exposure:				
Antibiotic at first visit	Reference		Reference	
Deferred antibiotic	12.36 (10.81 to14.13)	<0.001	7.12 (6.22 to 8.14)	<0.001
No antibiotic	16.70 (14.81 to 18.83)	<0.001	8.08 (7.12 to 9.16)	<0.001
Age group (years):				
65-74	Reference		Reference	
75-84	2.37 (2.08 to 2.71)	<0.001	1.59 (1.39 to 1.82)	<0.001
≥85	3.13 (2.73 to 3.58)	<0.001	1.67 (1.44 to 1.93)	<0.001
Sex:				
Men	Reference		Reference	
Women	0.25 (0.23 to 0.28)	<0.001	0.45 (0.40 to 0.50)	<0.001
Region:				
North of England and Yorkshire	Reference			
Midlands and East of England	1.02 (0.89 to 1.18)	0.74		
South of England	0.86 (0.75 to 0.99)	0.03		
London	0.90 (0.74 to 1.09)	0.28		
Index of multiple deprivation (fifths):				
1st (least deprived)	Reference		Reference	
2	1.00 (0.86 to 1.16)	0.98	0.97 (0.83 to 1.14)	0.74
3	1.07 (0.92 to 1.25)	0.38	1.04 (0.89 to 1.22)	0.58
4	1.35 (1.15 to 1.58)	<0.001	1.21 (1.03 to 1.42)	0.02
5th (most deprived)	1.39 (1.18 to 1.65)	<0.001	1.18 (0.99 to 1.40)	0.06
Charlson comorbidity index score (0-12)	1.35 (1.29 to 1.40)	<0.001	1.10 (1.04 to 1.16)	<0.001
Immunosuppressed	5.06 (1.26 to 20.31)	0.02		
Renal disease	1.61 (1.28 to 2.02)	<0.001		
Smoking	1.20 (0.95 to 1.52)	0.16		
Year of UTI:				
2007-08	Reference		Reference	
2008-09	0.70 (0.45 to 1.10)	0.12	0.67 (0.42 to 1.07)	0.09
2009-10	0.74 (0.48 to 1.16)	0.20	0.66 (0.42 to 1.05)	0.08
2010-11	0.97 (0.63 to 1.51)	0.90	0.86 (0.55 to 1.36)	0.53
2011-12	0.90 (0.58 to 1.40)	0.65	0.77 (0.49 to 1.22)	0.26
2012-13	1.98 (1.30 to 3.01)	0.001	1.57 (1.01 to 2.42)	0.04
2013-14	3.38 (2.24 to 5.12)	<0.001	2.72 (1.77 to 4.19)	<0.001
2014-15	4.52 (2.98 to 6.83)	<0.001	3.46 (2.25 to 5.32)	<0.001
Symptoms <30 days before UTI diagnosis	1.20 (1.01 to 1.44)	0.04		
Antibiotic prescribed <30 days before UTI diagnosis	1.26 (1.12 to 1.42)	<0.001		
Admitted to hospital 30 days before diagnosis	10.45 (9.44 to 11.57)	<0.001	3.94 (3.54 to 4.39)	<0.001
Indwelling urethral catheter	3.60 (2.66 to 4.89)	<0.001		
Recurrent UTIs	0.77 (0.67 to 0 0.88)	<0.001	0.86 (0.75 to 0.99)	0.04
Interaction antibiotic exposure and recurrence	1.27 (1.15 to 1.41)	<0.001		

* Standard errors adjusted for clustering using robust standard errors approach.

† All variables showing P<0.2 in univariate analyses (unadjusted results) were included and tested in multivariable logistic regression model (adjusted results).

The number needed to harm (NNH) estimate for bloodstream infection was lower (greater risk) with no antibiotics (NNH=37) than with deferred antibiotics (NNH=51), which means that on average for every 37 patients in the no antibiotic group and for every 51 patients in the deferred antibiotic group, one case of bloodstream infection would occur that would not have been seen with use of immediate antibiotics. No significant difference was observed between the rate of bloodstream infection for immediate trimethoprim treatment (233/148 333: 0.2%) and nitrofurantoin treatment (90/51 745: 0.2%, P=0.41).

The proportion of patients admitted to hospital after a UTI episode was nearly two times higher for those in the no antibiotics group (27.0%) and deferred antibiotics group (26.8%) compared with those in the immediate antibiotics (14.8%) group. Among cases admitted to hospital, the length of stay was significantly higher for the no antibiotics group (12.1 days *v* 6.3 days for immediate antibiotics group and 7.7 days for deferred antibiotics group) (table 1).

**Fig 3:**
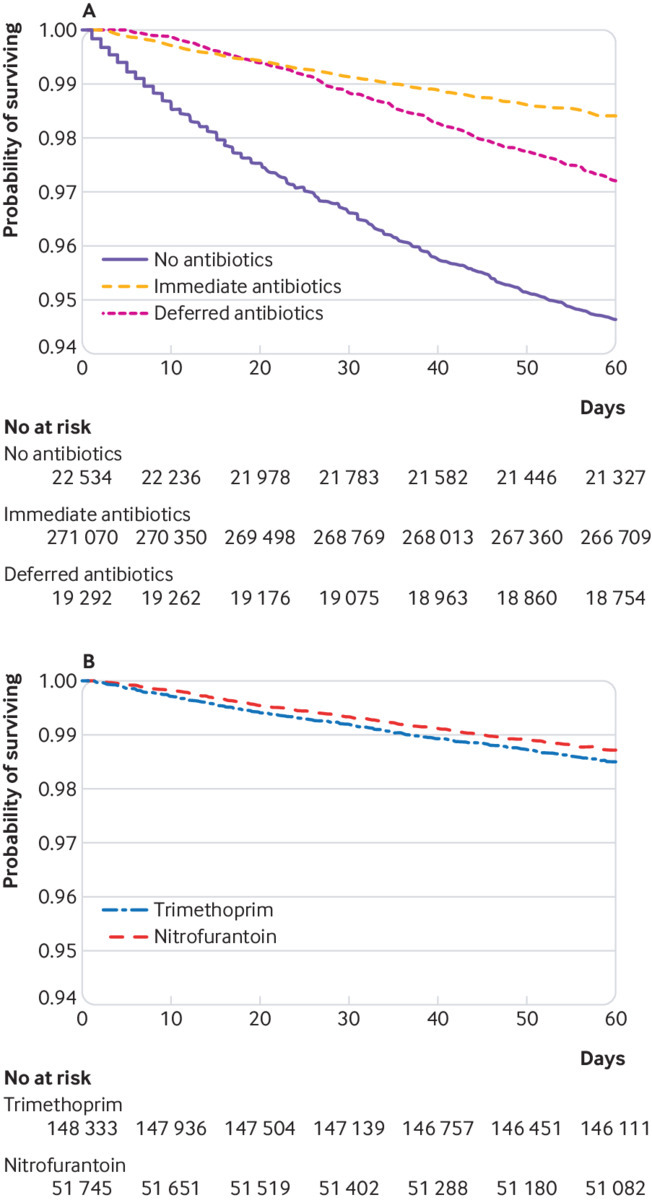
Kaplan-Meier survival curves by antibiotic management over 60 days

Finally, 2.0% (6193/312 896) of the participants older than 65 years who presented to their GP with a UTI died within 60 days; 5.4% (1217/22 534) for no antibiotics, 2.8% (545/19 292) for deferred antibiotics, and 1.6% (4431/271 070) for immediate antibiotics (table 1). The NNH estimate for death within 60 days was lower with no antibiotics (NNH=27) than with deferred antibiotics (NNH=83), with a calculated risk relative to immediate antibiotics. The Kaplan-Meier curves showed a significant reduction of the 60 day survival for older adults prescribed no antibiotics or deferred antibiotics compared with those prescribed immediate antibiotics (fig 3). Among the patients who were prescribed immediate antibiotics, a small but significant reduction of the 60 day survival was also observed for patients treated with trimethoprim (98.5%) compared with nitrofurantoin (98.7%, P<0.001) (fig 3).

The multivariable Cox regression analysis showed that compared with immediate antibiotics and after adjusting for covariates the risk of all cause mortality in older adults at any time during the 60 days of follow-up was 1.16 times higher with deferred antibiotics (adjusted hazard ratio 1.16, 95% confidence interval 1.06 to 1.27) and 2.18 times higher with no antibiotics (2.18, 2.04 to 2.33) (table 4). The sensitivity analyses excluding the antibiotic treatments with a duration of more than 21 days and 28 days showed consistent results. However, the magnitude of the associations between treatment groups were slightly higher for those prescribed deferred antibiotics (1.19, 1.14 to 1.23 and 1.36, 1.22 to 1.48, respectively) and no antibiotics (2.47, 2.28 to 2.63 and 2.61, 2.38 to 2.75) compared with those prescribed immediate antibiotics.

**Table 4:** Multivariable Cox regession analysis for 60 day all cause mortality after diagnosis of a urinary tract infection (UTI)

Variables	Unadjusted hazard ratio (95% CI)*	P value	Adjusted† hazard ratio (95% CI)*	P value
Antibiotic exposure:				
Antibiotic first visit	Reference		Reference	
Deferred antibiotic	1.73 (1.59 to 1.89)	<0.001	1.16 (1.06 to 1.27)	0.001
No antibiotics	3.38 (3.17 to 3.60)	<0.001	2.18 (2.04 to 2.33)	<0.001
Age group (years):				
65-74	Reference		Reference	
75-84	3.20 (2.94 to 3.48)	<0.001	2.79 (2.60 to 2.99)	<0.001
≥85	9.42 (8.71 to 10.19)	<0.001	7.87 (7.37 to 8.40)	<0.001
Sex:				
Men	Reference		Reference	
Women	0.42 (0.40 to 0.44)	<0.001	0.52 (0.49 to 0.55)	<0.001
Region:				
North of England and Yorkshire	Reference		Reference	
Midlands and east of England	0.98 (0.92 to 1.06)	0.67	0.97 (0.90 to 1.04)	0.39
South of England	0.93 (0.87 to 0.99)	0.025	0.93 (0.87 to 1.00)	0.05
London	0.69 (0.62 to 0.77)	<0.001	0.72 (0.65 to 0.80)	<0.001
Index of multiple deprivation (fifths):				
1st (least deprived)	Reference		Reference	
2nd	1.17 (1.08 to 1.26)	<0.001	1.16 (1.07 to 1.26)	<0.001
3rd	1.31 (1.22 to 1.42)	<0.001	1.27 (1.18 to 1.37)	<0.001
4th	1.29 (1.19 to 1.40)	<0.001	1.29 (1.19 to 1.40)	<0.001
5th (most deprived)	1.35 (1.23 to 1.47)	<0.001	1.33 (1.21 to 1.45)	<0.001
Charlson comorbidity index score (0-12)	1.50 (1.47 to 1.53)	<0.001	1.27 (1.21 to 1.33)	<0.001
Immunosuppressed	5.26 (2.60 to 10.65)	<0.001	5.09 (2.54 to 10.20)	<0.001
Renal disease	2.00 (1.81 to 2.22)	<0.001	1.81 (1.72 to 1.93)	0.002
Smoking	0.82 (0.71 to 0.94)	0.005	1.27 (1.10 to 1.46)	0.001
Year of UTI:				
2007-08	Reference		Reference	
2008-09	1.20 (1.00 to 1.45)	0.05	1.14 (0.94 to 1.37)	0.18
2009-10	1.17 (0.97 to 1.41)	0.09	1.09 (0.89 to 1.30)	0.42
2010-11	1.25 (1.04 to 1.51)	0.02	1.13 (0.92 to 1.36)	0.27
2011-12	1.29 (1.07 to 1.55)	0.008	1.09 (0.91 to 1.32)	0.36
2012-13	1.50 (1.25 to 1.81)	<0.001	1.23 (1.02 to 1.48)	0.02
2013-14	1.55 (1.29 to 1.87)	<0.001	1.24 (1.03 to 1.50)	0.02
2014-15	1.58 (1.30 to 1.91)	<0.001	1.23 (1.02 to 1.48)	0.04
Symptoms <30 days before UTI diagnosis	1.23 (1.13 to 1.34)	<0.001		
Antibiotic prescribed <30 days before UTI diagnosis	1.50 (1.42 to 1.58)	<0.001	1.31 (1.22 to 1.42)	<0.001
Admitted to hospital 30 days before diagnosis	3.24 (3.07 to 3.42)	<0.001	1.88 (1.77 to 2.03)	<0.001
Indwelling urethral catheter	2.71 (2.29 to 3.21)	<0.001		
Recurrent UTIs	0.92 (0.87 to 0.98)	0.008	0.89 (0.82 to 0.95)	<0.001
Interaction antibiotic exposure and recurrence	1.11 (1.06 to 1.17)	<0.001	1.19 (1.10 to 1.30)	<0.001

* Standard errors adjusted for clustering using robust standard errors approach.

† All variables showing P<0.2 in univariate analyses (unadjusted results) were included and tested in multivariable Cox regression model (adjusted results).

In the multivariable Cox regression model, being older, male, living in a deprived area, having a higher Charlson comorbidity index score, being a smoker, being immunosuppressed, having renal disease, and having been exposed to antibiotics and/or discharged from hospital 30 days before the UTI diagnosis, were all positively associated with 60 day all cause mortality. In contrast, living in London or the south of England compared with the north of England and Yorkshire or the east of England and the Midlands, as well as having recurrent UTIs, were significantly associated with a decrease in mortality. The interaction factor between antibiotic use and recurrent UTIs was also significant and thus included in the final multivariable model (table 4).

Finally, for the care pathway of older adults with a diagnosed UTI in primary care, 44.5% of patients with a UTI (n=139 359) presented only once to the GP without a subsequent hospital admission, whereas 38.2% (n=119 364) required multiple visits to the GP for the same UTI episode and 17.3% (n=54 173) were admitted to hospital within 60 days of their first visit for a UTI. Among patients who were not prescribed antibiotics, 29.5% (n=6637) were admitted to the hospital or died within 60 days, compared with 16.4% (n=47 536) among those who were prescribed antibiotics; 27.3% (n=18 065) of men were admitted to hospital or died within 60 days compared with 14.6% (n=36 108) of women. Older patients with UTI were more likely to be admitted to hospital compared with 65-74 year olds who were more likely to have a single visit to the GP (table 5).

**Table 5:** Care pathway of each episode of urinary tract infection (UTI) experienced by older adults with a diagnosis in primary care. Values are numbers (percentages, 95% confidence intervals) unless stated otherwise

Variables	No of participants	Single visit to GP	Multiple visits to GP	Hospital admission including death within 60 days	P value
Antibiotic exposure*:					
No antibiotics	22 534 (7.2; 7.1 to 7.3)	14 722 (65.33; 64.71 to 65.95)	1175 (5.2; 4.9 to 5.5)	6637 (29.5; 28.9 to 30.1)	<0.001
Antibiotics*	290 362 (92.8; 92.7 to 92.9)	124 637 (42.9; 42.7 to 43.1)	118 189 (40.7; 40.5 to 40.9)	47 536 (16.4; 16.2 to 16.5)	
Sex:					
Men	66 266 (21.2; 21.0 to 21.3)	24 561 (37.1; 36.7 to 37.4)	23 640 (35.7; 35.3 to 36.0)	18 065 (27.3; 26.9 to 27.6)	<0.001
Women	246 630 (78.8; 78.7 to 79.0)	114 798 (46.6; 46.4 to 46.7)	95 724 (38.8; 38.6 to 39.0)	36 108 (14.6; 14.5 to 14.8)	
Age group (years):					
65-74	136 175 (43.5; 43.4 to 43.7)	66 972 (49.2; 48.9 to 49.5)	51 336 (37.7; 37.4 to 38.0)	17 867 (13.1; 12.9 to 13.3)	<0.001
75-84	107 485 (34.4; 34.2 to 34.5)	45 909 (42.7; 42.4 to 43.0)	41 427 (38.5; 38.3 to 38.83	20 149 (18.8; 18.5 to 19.0)	
≥85	69 236 (22.1; 22.0 to 22.3)	26 478 (38.2; 37.9 to 38.6)	26 601 (38.4; 38.1 to 38.8)	16 157 (23.3; 23.02 to 23.7)	
Total	312 896	139 359 (44.5; 44.4 to 44.7)	119 364 (38.2; 38.0 to 38.3)	54 173 (17.3; 17.2 to 17.5)	

* Includes deferred and immediate antibiotic approaches.

## Discussion

This study has shown that patients aged older than 65 years with a diagnosis of urinary tract infection (UTI) in the community are at significantly increased risk of bloodstream infection and death within 60 days when antibiotic treatment was either not prescribed or deferred.

The odds of developing a bloodstream infection within 60 days was sevenfold and eightfold higher in the deferred antibiotic and no antibiotics groups, respectively, compared with the immediate antibiotics group. The number needed to harm (NNH) for bloodstream infection was lower with no antibiotics (NNH=37) than with deferred antibiotics (NNH=51), when both were compared with immediate antibiotics. Patients in the no antibiotics group were also more than twice as likely to die, whereas patients in the deferred antibiotics group were 1.16 as likely to die during the 60 days after a UTI compared with those in the immediate antibiotic group. The NNH estimate for death was lower with no antibiotics (NNH=27) than with deferred antibiotics (NNH=83).

These findings were adjusted for potential confounding factors and changes over time to account for updates to national guidelines. The risk of bloodstream infection and all cause mortality also increased for male and older patients, especially those older than 85 years and those living in more deprived areas. Among patients who were prescribed immediate antibiotics for an episode of UTI, a small but significant increase of the 60 day survival was observed for those treated with nitrofurantoin compared with trimethoprim. This increase could reflect either higher levels of resistance to trimethoprim^16^ or a healthier population treated with nitrofurantoin; the latest being not recommended for patients with poor kidney function.^28^ These results are consistent with a recent cohort study using data from Clinical Practice Research Datalink (CPRD), where nitrofurantoin was associated with the smallest odds of death within 14 days of antibiotic initiation for UTI of all the antibiotics investigated.^29^

### Strengths and limitations of this study

A major strength of this study is the use of individual patient level data for adults older than 65 years extracted from a large nationwide general practice records database and linked to hospital and mortality records. This provided the opportunity to track the care pathways of a vulnerable population with a diagnosis of UTI in the community with a 60 day follow-up. The linkage with mortality data from the Office for National Statistics minimised possible bias in the risk estimates of all cause mortality among older adults treated in a routine care setting.

The large sample size of about 160 000 patients with more than 300 000 distinct UTI episodes substantially increased the power of the analyses, especially for rare severe adverse events in older adults (ie, bloodstream infection, mortality). As the base population is representative of the English general population, our results are generalisable to the entire English population of elderly patients.

In addition, records were routinely collected by GPs in normal care settings providing an unbiased selection of both the exposed and the control cohorts and reducing the opportunity for information bias (as exposure and outcomes were prospectively collected independently). This study not only helped us to understand the management of UTI in an older population in real life but also enabled us to assess the no antibiotic treatment approach for UTI. This option would have been challenging in a prospective trial because of ethical restraints. Finally, we had access to detailed patient information, including patient diagnoses, comorbidities, prescribed drugs, and procedures, allowing us to control for the effects of several potential confounders in the multivariable regression models.

The main limitations of our study are common to observational studies using routinely collected electronic health record data, and include unmeasured and residual confounders, missing data and potential biases, such as confounding by indication, misclassification biases, or inconsistencies in coding within and between practices and over time.

Patients were identified and included in our study based on a clinical diagnosis recorded using a coding system. Therefore, most of the cases were suspected UTIs, with only a minority based on a laboratory confirmed diagnosis. Separate microbiology data with UTI confirmation and drug sensitivities were unavailable. We used a pragmatic approach to include all the possible descriptors a GP might use for infectious disease of the urinary tract. Further research using a more specific list of codes for UTI could be worth exploring.

The uncertainties around the UTI diagnosis in elderly patients as a result of uncommon presentations might have biased the selection of our initial cohort of patients with UTI. A variety of acute infectious or non-infectious causes leading to those uncertainties might have driven the adverse outcomes. CPRD only reports the symptoms documented by GPs and does not always include a structured assessment of the illness with information on symptom severity and onset, for example, which made the control for confounding by indication difficult. However, we may have observed that the protective effect of immediate antibiotics would exceed the effect of confounding by indication as described by Little et al.^30^

There were potential classification biases for the exposure variable associated with the lack of information on treatment compliance by the patients and on delayed prescriptions issued by GPs at the index visit in CPRD. The database did not define whether the antibiotics prescribed on the date of the initial UTI diagnosis had to be taken immediately or several days later in the context of ongoing symptoms. This common delayed prescription strategy to reduce inappropriate antibiotic prescribing, as well as patients who did not consume the antibiotics prescribed by the GPs, may have incorrectly classified some patients as belonging to the immediate antibiotics group. Conversely, the database did not identify patients who had already accessed antibiotics (rescue pack or previous prescription). This might partly explain the observation (see table 1) that more patients in the deferred and no antibiotics groups had received antibiotics or had been admitted to the hospital in the previous month compared with the immediate antibiotics group. We also did not consider the number of days between the date of the initial UTI diagnosis and the date of deferred antibiotics when a prescription was not issued at the index visit, which may have an impact on the adverse outcomes.

We cannot exclude an alternative non-urinary source for the bloodstream infections. The origin of the bloodstream infections is not often specified in hospital episode statistics or CPRD. In the context of the cohort of patients in this study initially having a diagnosis of a UTI in primary care, most of the bloodstream infections recorded should have a urinary source. Reverse causality was unlikely in this study as we have tried to make sure that the date of the exposure (antibiotic management) was before the outcomes (bloodstream infections or mortality, or both).

Finally, the complexity of the coding system in electronic health record databases, the variability in recording information, as well as missing data, might have also prevented us from capturing a comprehensive list of the complications related to UTI and the confounders associated with increased risk of bloodstream infection and all cause mortality. For example, some nursing homes may have a wait and see policy for antibiotic prescribing to prevent *Clostridium difficile* outbreaks. It is also possible that patients with cognitive impairment lack insight into the severity of their illness and were not prescribed antibiotics while they were needed. By adjusting our outcomes on existing comorbidities using the Charlson comorbidity index, we have tried to minimise the presence of some residual confounders.

### Comparison with existing literature

This study comprised a large sample size population, assessing the real life care management, including no antibiotics and deferred antibiotics, as well as the outcomes and care pathway of older adults with a diagnosis of UTI in primary care. Limited evidence is available to support the choice of no antibiotics or of deferred antibiotics for the management of UTI in primary care, as ethical concerns have prevented placebo controlled studies for UTI.^31^

A systematic review of randomised controlled trials showed that antibiotic treatment is more effective at achieving faster symptom relief, microbiological clearance, and lower reinfection rates than placebo for uncomplicated cystitis in women aged 15 to 84 years.^32^ However, potential unintended adverse events have not been explored (eg, admission to hospital, bloodstream infection, or death) as a large sample size would be needed to capture these rare serious adverse events. Another randomised controlled trial, which evaluated the efficacy of initial symptomatic treatment with ibuprofen versus immediate antibiotic treatment in uncomplicated UTI for women younger than 65 years has shown an increase in the total burden of symptoms and pyelonephritis cases in the ibuprofen arm.^33^

In the context of randomised controlled trials, strict exclusion criteria particularly related to age have been applied that prevent the results being generalised to older adult populations who may require a different approach in the management of UTI. In contrast, our study specifically looked at the group of older patients (>65 years) who are more susceptible to complications and are often neglected in UTI related research. We showed that antibiotics prescribed at the time of UTI diagnosis may benefit this vulnerable population by significantly reducing the risk of all cause mortality and the rate of bloodstream infection and hospital admission.

Recent guidance from the National Institute of Health and Care Excellence has proposed no antibiotic or delayed antibiotic prescriptions when infection is likely to be self limiting in an effort to reduce inappropriate prescribing.^15^ ^34^ Evidence and recommendations, however, refer mainly to upper respiratory tract infections.^30^ ^35^ Evidence is nonetheless emerging that delayed prescribing in the treatment of UTI is becoming more acceptable in practice.^36^ ^37^

A randomised controlled trial evaluated various antibiotic management strategies, including empirical delayed (by 48 hours) antibiotics and immediate antibiotics strategies for UTI in women younger than 70 years. No significant differences in symptom duration, severity, or frequency of symptoms between the strategies were reported.^38^ In our study, deferred antibiotics were associated with less severe adverse outcomes than no antibiotics for older adults but still showed a significantly higher risk of mortality compared with immediate antibiotics.

The question remains as to why a significant proportion (about 7%) of vulnerable older patients had a diagnosis of UTI but were not prescribed antibiotics. It could be patient or doctor choice, but it is also possible that antimicrobial stewardship programmes and quality premium payments are encouraging a culture of more judicious antibiotic use. Public Health England recently reported a 13.2% reduction in antibiotic prescribing in primary care between 2013 and 2017.^16^

There is also a major concern about the risk of *C difficile* infection in elderly people associated with antibiotic use, which also includes trimethoprim.^39^ ^40^

Other circumstances, such as the presence of mild urinary symptoms, may encourage clinicians to withhold antibiotics in the context of a working diagnosis of UTI. Nevertheless, if this explanation holds true, patients with disease not severe enough to prompt antibiotic treatment are at risk of severe consequences.

### Clinical, policy, and research implications

Our findings suggest that GPs consider early prescription of antibiotics for this vulnerable group of older adults in view of their increased susceptibility to sepsis after UTI and despite a growing pressure to reduce inappropriate antibiotic use. Particular care is needed for the management of older men and those in deprived communities. For researchers, there is a need to improve the understanding of the effects of deferred antibiotic prescribing in routine practice. New medical record or retrievable codes should therefore be in place to record when primary care clinicians advise patients to delay antibiotic consumption.

### Conclusion

Results from this large population based cohort study suggest a significant increase in the risk of bloodstream infection and all cause mortality and the rate of hospital admission associated with no antibiotics and deferred antibiotics compared with immediate antibiotics in older adults with a diagnosis of UTI in primary care. Our study suggests the early initiation of antibiotics for UTI in older high risk adult populations (especially men aged >85 years) should be recommended to prevent serious complications.

**What is already known on this topic**

About half of *Escherichia coli* bloodstream infections are caused by an underlying urinary tract infection (UTI), with higher risk seen in elderly people
While “no antibiotic” or “delayed or deferred antibiotic” treatment is often not associated with severe adverse outcomes for some self limiting illnesses (eg, upper respiratory tract infections), a slight increase in symptom duration and complication rate have been reported for UTI in young women
The generalisability of these studies, however, is limited because of sample size and study population demographics

**What this study adds**

After adjustment for key covariates, no antibiotics and deferred antibiotic approaches for the management of UTI in older adults in primary care appears to be associated with a significant increased risk of bloodstream infection and all cause mortality compared with an immediate antibiotics approach

## Web extra

Extra material supplied by authors

## Notes

Contributors: MG is first author. MG, HL, RG, PA, MM, and APJ designed the study. MG, HL, and RG wrote the protocol. MG, JHD, HL,RG, and MM carried out the data management and the code list identification. MG and JHD carried out the statistical analysis and wrote the first draft. All the authors contributed substantially to drafts and critical revision and approved the final manuscript. PA is the guarantor. The corresponding author attests that all listed authors meet authorship criteria and that no others meeting the criteria have been omitted.

Ethical approval: The protocol for research using the Clinical Practice Research Datalink (CPRD) was approved by the Independent Scientific Advisory Committee (CPRD No 16_026R).

Data sharing: CPRD data were provided under a licence that does not permit sharing. Data are, however, obtainable from CPRD under standard conditions.

Transparency: The manuscript’s guarantor (PA) affirms that the manuscript is an honest, accurate, and transparent account of the study being reported; that no important aspects of the study have been omitted; and that any discrepancies from the study as planned (and, if relevant, registered) have been explained.
