# Supplementary material for: Antibiotic management of urinary tract infection in elderly patients in primary care and its association with bloodstream infections and all cause mortality: population based cohort study
Source: BMJ. 2019 Feb 27;364:l525. doi: 10.1136/bmj.l525 (PMC6391656; doi:10.1136/bmj.l525)
Supplement: Supplementary file 1 — Supplementary information: tables S1-S3 [file gham045885.wt1.pdf]

Table S1: Read codes used to identify patients with UTI, complications of UTI and BSI in CPRD

**Read codes for identifying patients with a UTI at the cohort selection stage**	
1AG..00	Recurrent urinary tract infections
1J4..00	Suspected UTI
K15..00	Cystitis
K150.00	Acute cystitis
K15z.00	Cystitis NOS
K190.00	Urinary tract infection, site not specified
K190.11	Recurrent urinary tract infection
K190100	Pyuria, site not specified
K190200	Post-operative urinary tract infection
K190300	Recurrent urinary tract infection
K190311	Recurrent UTI
K190400	Chronic urinary tract infection
K190500	Urinary tract infection
K190z00	Urinary tract infection, site not specified NOS
SP07700	Infect+inflam react due pros dev, implt+graft in urinary syst
SP07Q00	Catheter-associated urinary tract infection
SP07Q11	CAUTI - catheter-associated urinary tract infection
K15y.00	Other specified cystitis
K152z00	Other chronic cystitis NOS
K152.00	Other chronic cystitis
K152y00	Chronic cystitis unspecified
K155.00	Recurrent cystitis
K15yz00	Other cystitis NOS
1AZ6000	Mild lower urinary tract symptoms
7N51.00	[SO]Lower urinary tract
1AZ6100	Moderate lower urinary tract symptoms
1AZ6.00	Lower urinary tract symptoms
Kyu5100	[X]Other cystitis
14D4.00	H/O: recurrent cystitis
**Read codes for identifying upper UTIs or complications of UTIs in CPRD data**	
K100.00	Chronic pyelonephritis
K100000	Chronic pyelonephritis without medullary necrosis
K100100	Chronic pyelonephritis with medullary necrosis
K100400	Nonobstructive reflux-associated chronic pyelonephritis
K100500	Chronic obstructive pyelonephritis
K100600	Calculous pyelonephritis
K100z00	Chronic pyelonephritis NOS
K101.00	Acute pyelonephritis
K101000	Acute pyelonephritis without medullary necrosis
K101z00	Acute pyelonephritis NOS
K104.00	Xanthogranulomatous pyelonephritis

K10y.00	Pyelonephritis and pyonephrosis unspecified
K10y000	Pyelonephritis unspecified
K10y300	Pyelonephritis in diseases EC
K10yz00	Unspecified pyelonephritis NOS
K100200	Chronic pyelitis
K10y400	Pyelitis in diseases EC
K101200	Acute pyelitis
K10y100	Pyelitis unspecified
K102000	Renal abscess
K102.00	Renal and perinephric abscess
K102100	Perinephric abscess
K102z00	Renal and perinephric abscess NOS
K10..00	Infections of kidney
K10z.00	Infection of kidney NOS
K10..11	Renal infections
K10..00	Infections of kidney
K10z.00	Infection of kidney NOS
K21..11	Prostatitis and other inflammatory diseases of prostate
K210.00	Acute prostatitis
K211.00	Chronic prostatitis
K214.00	Prostatitis in diseases EC
K214z00	Prostatitis in diseases EC NOS
K21z.00	Prostatitis NOS
K213.00	Prostatocystitis
K212.00	Abscess of prostate
K10y200	Pyonephrosis unspecified
K105.00	Chronic infective interstitial nephritis
A160200	Tuberculous pyelonephritis
A160100	Tuberculous pyelitis
**Read codes for identifying bloodstream infection/sepsis in CPRD data**	
A38z.11	Sepsis
A38..00	Septicaemia
A3C..00	Sepsis
K190600	Urosepsis
A38z.00	Septicaemia NOS
A381.00	Staphylococcal septicaemia
R106.00	[D]Unspecified bacteraemia
A380.00	Streptococcal septicaemia
A382.00	Pneumococcal septicaemia
A384200	Escherichia coli septicaemia
A384211	E.coli septicaemia
A384.00	Septicaemia due to other gram negative organisms
A3Cz.00	Sepsis NOS
A3Cy.00	Other specified sepsis

A381000	Septicaemia due to Staphylococcus aureus
A38y.00	Other specified septicaemias
A380100	Septicaemia due to streptococcus, group B
A384300	Pseudomonas septicaemia
A384000	Gram negative septicaemia NOS
A380300	Septicaemia due to streptococcus pneumoniae
A384100	Haemophilus influenzae septicaemia
A380400	Septicaemia due to enterococcus
A380000	Septicaemia due to streptococcus, group A
Ayu3J00	[X]Septicaemia, unspecified
AB2y300	Candidal septicaemia
A270100	Listeria septicaemia
A383.00	Septicaemia due to anaerobes
A3C3.00	Sepsis due to Gram negative bacteria
A381100	Septicaemia due to coagulase-negative staphylococcus
A3C0100	Sepsis due to Streptococcus group B
A380500	Vancomycin resistant enterococcal septicaemia
A3C1.00	Sepsis due to Staphylococcus
A384400	Serratia septicaemia
A3C2.11	Sepsis due to anaerobes
AB2y500	Candidal sepsis
A3C1000	Sepsis due to Staphylococcus aureus
A3C0300	Sepsis due to Streptococcus pneumoniae
A3C0000	Sepsis due to Streptococcus group A
A3C0.00	Sepsis due to Streptococcus
A270611	Listerial sepsis
A384z00	Other gram negative septicaemia NOS
A3C0z00	Streptococcal sepsis, unspecified
A3C0y00	Other streptococcal sepsis
Ayu3F00	[X]Streptococcal septicaemia, unspecified
A396.00	Sepsis due to Actinomyces
A3C2.00	Sepsis due to anaerobic bacteria
Ayu3E00	[X]Other streptococcal septicaemia
A271100	Erysipelothrix septicaemia
Ayu3G00	[X]Septicaemia due to other gram-negative organisms
A3C3.11	Sepsis due to Gram negative organisms
AB2y511	Sepsis due to Candida
A3C3y00	Sepsis due to other Gram negative organisms
A270600	Sepsis due to Listeria monocytogenes
Ayu3H00	[X]Other specified septicaemia

Table S2: ICD-10 codes used to identify patients with a complication following an episode of UTI and with a BSI in HES

ICD-10 codes for identifying upper UTI/advanced UTI in HES data (pyelonephritis, pyelitis, perinephric abscess, renal abscesses, and prostatitis)	
N10	Acute tubulo-interstitial nephritis
N11	Chronic tubulo-interstitial nephritis
N11.0	Nonobstructive reflux-associated chronic pyelonephritis
N11.1	Chronic obstructive pyelonephritis
N11.8	Other chronic tubulo-interstitial nephritis
N11.9	Chronic tubulo-interstitial nephritis, unspecified
N12	Tubulo-interstitial nephritis, not specified as acute or chronic
N13	Obstructive and reflux uropathy
N13.2	Hydronephrosis with renal and ureteral calculous obstruction
N20	Calculus of kidney and ureter
N20.9	Urinary calculus, unspecified
N28	Other disorders of kidney and ureter, not elsewhere classified
N28.8	Other specified disorders of kidney and ureter
N16.0*	Renal tubulo-interstitial disorders in infectious and parasitic diseases classified elsewhere
N15	Other renal tubulo-interstitial diseases
N15.1	Renal and perinephric abscess
N41	Inflammatory diseases of prostate
N41.0	Acute prostatitis
N41.1	Chronic prostatitis
N41.3	Prostatocystitis
N41.8	Other inflammatory diseases of prostate
N41.9	Inflammatory disease of prostate, unspecified
N28.84	Pyelitis cystica
N28.85	Pyeloureteritis cystica
N28.86	Ureteritis cystica
N34	Urethral Abscess
N139	Obstructive and reflux uropathy, unspecified
**ICD-10 codes for identifying bloodstream infection/sepsis in HES data**	
A41.5	Sepsis due to other Gram-negative organisms
A41.51	Sepsis due to Escherichia coli [E. coli]
A41.52	Sepsis due to Pseudomonas
A41.53	Sepsis due to Serratia
A41.59	Other Gram-negative sepsis
A41.8	Other specified sepsis
A41.81	Sepsis due to Enterococcus
A41.9	Sepsis, unspecified
R57.2	Septic shock
R65.1	Systemic Inflammatory Response Syndrome of infectious origin with organ failure
R65.0	Systemic Inflammatory Response Syndrome of infectious origin without organ failure
A40	Streptococcal sepsis
A41	Other septicaemia
R78.81	Bacteremia
R65.20	Severe sepsis without septic shock
R65.21	Severe sepsis with septic shock

Table S3: Read codes used to construct the Charlson Comorbidity Index (CCI) in CPRD

Classification	Read_code	Description
cancer	1O0..00	Cancer confirmed
cancer	4C53.00	Bone marrow: myeloma cells
cancer	4C54.00	Bone marrow: tumour cells
cancer	4D56.00	Pleural fluid: malignant cells
cancer	4E33.00	Sputum: malignant cells
cancer	4F32.00	Ascitic fluid: malignant cells
cancer	4K24.00	Cerv.smear: severe dyskaryosis
cancer	4K24.11	CIN III - severe dyskaryosis
cancer	4K2L.00	Cervical smear - high grade dyskaryosis (severe)
cancer	4K2M.00	invasive squamous carcinoma
cancer	4M...00	Tumour staging
cancer	4M0..00	Gleason grading of prostate cancer
cancer	4M00.00	Gleason prostate grade 2-4 (low)
cancer	4M01.00	Gleason prostate grade 5-7 (medium)
cancer	4M02.00	Gleason prostate grade 8-10 (high)
cancer	4M1..00	Dukes staging system
cancer	4M10.00	Dukes stage A
cancer	4M11.00	Dukes stage B
cancer	4M12.00	Dukes stage C1
cancer	4M13.00	Dukes stage C2
cancer	4M14.00	Dukes stage D
cancer	4M2..00	Lymphoma staging system
cancer	4M20.00	Lymphoma stage I
cancer	4M21.00	Lymphoma stage II
cancer	4M22.00	Lymphoma stage III
cancer	4M23.00	Lymphoma stage IV
cancer	4M3..00	Breslow depth staging for melanoma
cancer	4M4..00	FIGO staging of gynaecological malignancy
cancer	4M5..00	TNM tumour staging
cancer	4M6..00	Recurrence of tumour
cancer	4M7..00	Clark staging levels
cancer	4M70.00	Clark melanoma level 1
cancer	4M71.00	Clark melanoma level 2
cancer	4M72.00	Clark melanoma level 3
cancer	4M73.00	Clark melanoma level 4
cancer	4M74.00	Clark melanoma level 5
cancer	68W2400	Bowel scope (flexible sigmoidoscopy) screen: cancer
cancer	9Ow1.00	Bowel cancer detected by national screening programme
cancer	A788W00	HIV dis resulting in unspecified malignant neoplasm

cancer	A789600	HIV dis resulting in Burkitt's lymphoma
cancer	A789700	HIV dis resulting oth types of non-Hodgkin's lymphoma
cancer	A789800	HIV dis resulting in multiple malignant neoplasms
cancer	A789X00	HIV reslt/oth mal neopl/lymph,hematopoetc+reltd tissu
cancer	AyuC600	[X]HIV dis resulting in other non-Hodgkin's lymphoma
cancer	B....11	Cancers
cancer	B0...00	Malignant neoplasm of lip, oral cavity and pharynx
cancer	B0...11	Carcinoma of lip, oral cavity and pharynx
cancer	B00..00	Malignant neoplasm of lip
cancer	B00..11	Carcinoma of lip
cancer	B000.00	Malignant neoplasm of upper lip, vermilion border
cancer	B000000	Malignant neoplasm of upper lip, external
cancer	B000100	Malignant neoplasm of upper lip, lipstick area
cancer	B000z00	Malignant neoplasm of upper lip, vermilion border NOS
cancer	B001.00	Malignant neoplasm of lower lip, vermilion border
cancer	B001000	Malignant neoplasm of lower lip, external
cancer	B001100	Malignant neoplasm of lower lip, lipstick area
cancer	B001z00	Malignant neoplasm of lower lip, vermilion border NOS
cancer	B002.00	Malignant neoplasm of upper lip, inner aspect
cancer	B002100	Malignant neoplasm of upper lip, frenulum
cancer	B002200	Malignant neoplasm of upper lip, mucosa
cancer	B002300	Malignant neoplasm of upper lip, oral aspect
cancer	B002z00	Malignant neoplasm of upper lip, inner aspect NOS
cancer	B003.00	Malignant neoplasm of lower lip, inner aspect
cancer	B003000	Malignant neoplasm of lower lip, buccal aspect
cancer	B003100	Malignant neoplasm of lower lip, frenulum
cancer	B003200	Malignant neoplasm of lower lip, mucosa
cancer	B003300	Malignant neoplasm of lower lip, oral aspect
cancer	B003z00	Malignant neoplasm of lower lip, inner aspect NOS
cancer	B004.00	Malignant neoplasm of lip unspecified, inner aspect
cancer	B004000	Malignant neoplasm of lip unspecified, buccal aspect
cancer	B004200	Malignant neoplasm of lip unspecified, mucosa
cancer	B004300	Malignant neoplasm of lip, oral aspect
cancer	B005.00	Malignant neoplasm of commissure of lip
cancer	B006.00	Malignant neoplasm of overlapping lesion of lip
cancer	B007.00	Malignant neoplasm of lip, unspecified
cancer	B00z000	Malignant neoplasm of lip, unspecified, external
cancer	B00z100	Malignant neoplasm of lip, unspecified, lipstick area
cancer	B00zz00	Malignant neoplasm of lip, vermilion border NOS
cancer	B01..00	Malignant neoplasm of tongue
cancer	B010.00	Malignant neoplasm of base of tongue
cancer	B010.11	Malignant neoplasm of posterior third of tongue
cancer	B010000	Malignant neoplasm of base of tongue dorsal surface
cancer	B010z00	Malignant neoplasm of fixed part of tongue NOS

cancer	B011.00	Malignant neoplasm of dorsal surface of tongue
cancer	B011100	Malignant neoplasm of midline of tongue
cancer	B011z00	Malignant neoplasm of dorsum of tongue NOS
cancer	B012.00	Malignant neoplasm of tongue, tip and lateral border
cancer	B013.00	Malignant neoplasm of ventral surface of tongue
cancer	B013000	Malignant neoplasm of anterior 2/3 of tongue ventral
cancer	B013100	Malignant neoplasm of frenulum linguae
cancer	B013z00	Malignant neoplasm of ventral tongue surface NOS
cancer	B014.00	Malignant neoplasm of anterior 2/3 of tongue unspecified
cancer	B015.00	Malignant neoplasm of tongue, junctional zone
cancer	B016.00	Malignant neoplasm of lingual tonsil
cancer	B017.00	Malignant overlapping lesion of tongue
cancer	B01y.00	Malignant neoplasm of other sites of tongue
cancer	B01z.00	Malignant neoplasm of tongue NOS
cancer	B02..00	Malignant neoplasm of major salivary glands
cancer	B020.00	Malignant neoplasm of parotid gland
cancer	B021.00	Malignant neoplasm of submandibular gland
cancer	B022.00	Malignant neoplasm of sublingual gland
cancer	B02y.00	Malignant neoplasm of other major salivary glands
cancer	B02z.00	Malignant neoplasm of major salivary gland NOS
cancer	B03..00	Malignant neoplasm of gum
cancer	B030.00	Malignant neoplasm of upper gum
cancer	B031.00	Malignant neoplasm of lower gum
cancer	B03y.00	Malignant neoplasm of other sites of gum
cancer	B03z.00	Malignant neoplasm of gum NOS
cancer	B04..00	Malignant neoplasm of floor of mouth
cancer	B040.00	Malignant neoplasm of anterior portion of floor of mouth
cancer	B041.00	Malignant neoplasm of lateral portion of floor of mouth
cancer	B042.00	Malignant neoplasm, overlapping lesion of floor of mouth
cancer	B04y.00	Malignant neoplasm of other sites of floor of mouth
cancer	B04z.00	Malignant neoplasm of floor of mouth NOS
cancer	B05..00	Malignant neoplasm of other/unspecified parts of mouth
cancer	B050.00	Malignant neoplasm of cheek mucosa
cancer	B050.11	Malignant neoplasm of buccal mucosa
cancer	B051.00	Malignant neoplasm of vestibule of mouth
cancer	B051000	Malignant neoplasm of upper buccal sulcus
cancer	B051100	Malignant neoplasm of lower buccal sulcus
cancer	B052.00	Malignant neoplasm of hard palate
cancer	B053.00	Malignant neoplasm of soft palate
cancer	B054.00	Malignant neoplasm of uvula
cancer	B055.00	Malignant neoplasm of palate unspecified
cancer	B055000	Malignant neoplasm of junction of hard and soft palate
cancer	B055100	Malignant neoplasm of roof of mouth
cancer	B055z00	Malignant neoplasm of palate NOS

cancer	B056.00	Malignant neoplasm of retromolar area
cancer	B05y.00	Malignant neoplasm of other specified mouth parts
cancer	B05z.00	Malignant neoplasm of mouth NOS
cancer	B05z000	Kaposi's sarcoma of palate
cancer	B06..00	Malignant neoplasm of oropharynx
cancer	B060.00	Malignant neoplasm of tonsil
cancer	B060000	Malignant neoplasm of faucial tonsil
cancer	B060100	Malignant neoplasm of palatine tonsil
cancer	B060200	Malignant neoplasm of overlapping lesion of tonsil
cancer	B060z00	Malignant neoplasm tonsil NOS
cancer	B061.00	Malignant neoplasm of tonsillar fossa
cancer	B062.00	Malignant neoplasm of tonsillar pillar
cancer	B062000	Malignant neoplasm of faucial pillar
cancer	B062100	Malignant neoplasm of glossopalatine fold
cancer	B062200	Malignant neoplasm of palatoglossal arch
cancer	B062300	Malignant neoplasm of palatopharyngeal arch
cancer	B062z00	Malignant neoplasm of tonsillar fossa NOS
cancer	B063.00	Malignant neoplasm of vallecula
cancer	B064.00	Malignant neoplasm of anterior epiglottis
cancer	B064000	Malignant neoplasm of epiglottis, free border
cancer	B064100	Malignant neoplasm of glossoepiglottic fold
cancer	B064z00	Malignant neoplasm of anterior epiglottis NOS
cancer	B065.00	Malignant neoplasm of junctional region of epiglottis
cancer	B066.00	Malignant neoplasm of lateral wall of oropharynx
cancer	B067.00	Malignant neoplasm of posterior wall of oropharynx
cancer	B06y.00	Malignant neoplasm of oropharynx, other specified sites
cancer	B06yz00	Malignant neoplasm of other specified site of oropharynx
cancer	B06z.00	Malignant neoplasm of oropharynx NOS
cancer	B07..00	Malignant neoplasm of nasopharynx
cancer	B070.00	Malignant neoplasm of roof of nasopharynx
cancer	B071.00	Malignant neoplasm of posterior wall of nasopharynx
cancer	B071000	Malignant neoplasm of adenoid
cancer	B071100	Malignant neoplasm of pharyngeal tonsil
cancer	B071z00	Malignant neoplasm of posterior wall of nasopharynx
cancer	B072.00	Malignant neoplasm of lateral wall of nasopharynx
cancer	B072000	Malignant neoplasm of pharyngeal recess
cancer	B072z00	Malignant neoplasm of lateral wall of nasopharynx NOS
cancer	B073.00	Malignant neoplasm of anterior wall of nasopharynx
cancer	B073100	Malignant neoplasm of nasopharyngeal soft palate
cancer	B073200	Malignant neoplasm poste margin nasal septum/ choanae
cancer	B073z00	Malignant neoplasm of anterior wall of nasopharynx NOS
cancer	B074.00	Malignant neoplasm, overlapping lesion of nasopharynx
cancer	B07y.00	Malignant neoplasm of other specified site nasopharynx
cancer	B07z.00	Malignant neoplasm of nasopharynx NOS

cancer	B08..00	Malignant neoplasm of hypopharynx
cancer	B080.00	Malignant neoplasm of postcricoid region
cancer	B081.00	Malignant neoplasm of pyriform sinus
cancer	B082.00	Malignant neoplasm aryepiglottic fold, hypopharyngeal
cancer	B083.00	Malignant neoplasm of posterior pharynx
cancer	B08y.00	Malignant neoplasm of other specified hypopharyngeal
cancer	B08z.00	Malignant neoplasm of hypopharynx NOS
cancer	B0z..00	Malig neop other/ill-defined sites lip, oral cavity, pharynx
cancer	B0z0.00	Malignant neoplasm of pharynx unspecified
cancer	B0z1.00	Malignant neoplasm of Waldeyer's ring
cancer	B0z2.00	Malignant neoplasm of laryngopharynx
cancer	B0zy.00	Malignant neoplasm of other sites lip, oral cavity, pharynx
cancer	B0zz.00	Malignant neoplasm of lip, oral cavity and pharynx NOS
cancer	B1...00	Malignant neoplasm of digestive organs and peritoneum
cancer	B1...11	Carcinoma of digestive organs and peritoneum
cancer	B10..00	Malignant neoplasm of oesophagus
cancer	B100.00	Malignant neoplasm of cervical oesophagus
cancer	B101.00	Malignant neoplasm of thoracic oesophagus
cancer	B102.00	Malignant neoplasm of abdominal oesophagus
cancer	B103.00	Malignant neoplasm of upper third of oesophagus
cancer	B104.00	Malignant neoplasm of middle third of oesophagus
cancer	B105.00	Malignant neoplasm of lower third of oesophagus
cancer	B106.00	Malignant neoplasm, overlapping lesion of oesophagus
cancer	B107.00	Siewert type I adenocarcinoma
cancer	B10y.00	Malignant neoplasm of other specified part oesophagus
cancer	B10z.00	Malignant neoplasm of oesophagus NOS
cancer	B10z.11	Oesophageal cancer
cancer	B11..00	Malignant neoplasm of stomach
cancer	B110.00	Malignant neoplasm of cardia of stomach
cancer	B110000	Malignant neoplasm of cardiac orifice of stomach
cancer	B110100	Malignant neoplasm of cardiooesophageal junct stomach
cancer	B110111	Malignant neoplasm of gastro-oesophageal junction
cancer	B110z00	Malignant neoplasm of cardia of stomach NOS
cancer	B111.00	Malignant neoplasm of pylorus of stomach
cancer	B111000	Malignant neoplasm of prepylorus of stomach
cancer	B111100	Malignant neoplasm of pyloric canal of stomach
cancer	B111z00	Malignant neoplasm of pylorus of stomach NOS
cancer	B112.00	Malignant neoplasm of pyloric antrum of stomach
cancer	B113.00	Malignant neoplasm of fundus of stomach
cancer	B114.00	Malignant neoplasm of body of stomach
cancer	B115.00	Malignant neoplasm of lesser curve stomach unspecified
cancer	B116.00	Malignant neoplasm of greater curve stomach unspecified
cancer	B117.00	Malignant neoplasm, overlapping lesion of stomach
cancer	B118.00	Siewert type II adenocarcinoma

cancer	B119.00	Siewert type III adenocarcinoma
cancer	B11y.00	Malignant neoplasm of other specified site of stomach
cancer	B11y000	Malignant neoplasm of anterior wall of stomach NEC
cancer	B11y100	Malignant neoplasm of posterior wall of stomach NEC
cancer	B11yz00	Malignant neoplasm of other specified site of stomach
cancer	B11z.00	Malignant neoplasm of stomach NOS
cancer	B12..00	Malignant neoplasm of small intestine and duodenum
cancer	B120.00	Malignant neoplasm of duodenum
cancer	B121.00	Malignant neoplasm of jejunum
cancer	B122.00	Malignant neoplasm of ileum
cancer	B123.00	Malignant neoplasm of Meckel's diverticulum
cancer	B124.00	Malignant neoplasm, overlapping lesion of small intestine
cancer	B12y.00	Malignant neoplasm of other specified site small intestine
cancer	B12z.00	Malignant neoplasm of small intestine NOS
cancer	B13..00	Malignant neoplasm of colon
cancer	B130.00	Malignant neoplasm of hepatic flexure of colon
cancer	B131.00	Malignant neoplasm of transverse colon
cancer	B132.00	Malignant neoplasm of descending colon
cancer	B133.00	Malignant neoplasm of sigmoid colon
cancer	B134.00	Malignant neoplasm of caecum
cancer	B134.11	Carcinoma of caecum
cancer	B135.00	Malignant neoplasm of appendix
cancer	B136.00	Malignant neoplasm of ascending colon
cancer	B137.00	Malignant neoplasm of splenic flexure of colon
cancer	B138.00	Malignant neoplasm, overlapping lesion of colon
cancer	B139.00	Hereditary nonpolyposis colon cancer
cancer	B13y.00	Malignant neoplasm of other specified sites of colon
cancer	B13z.00	Malignant neoplasm of colon NOS
cancer	B13z.11	Colonic cancer
cancer	B14..00	Malignant neoplasm rectum/rectosigmoid junction/ anus
cancer	B140.00	Malignant neoplasm of rectosigmoid junction
cancer	B141.00	Malignant neoplasm of rectum
cancer	B141.11	Carcinoma of rectum
cancer	B141.12	Rectal carcinoma
cancer	B142.00	Malignant neoplasm of anal canal
cancer	B142.11	Anal carcinoma
cancer	B142000	Malignant neoplasm of cloacogenic zone
cancer	B143.00	Malignant neoplasm of anus unspecified
cancer	B14y.00	Malig neop other site rectum/rectosigmoid junction/ anus
cancer	B15..00	Malignant neoplasm of liver and intrahepatic bile ducts
cancer	B150.00	Primary malignant neoplasm of liver
cancer	B150000	Primary carcinoma of liver
cancer	B150100	Hepatoblastoma of liver
cancer	B150200	Primary angiosarcoma of liver

cancer	B150300	Hepatocellular carcinoma
cancer	B150z00	Primary malignant neoplasm of liver NOS
cancer	B151.00	Malignant neoplasm of intrahepatic bile ducts
cancer	B151000	Malignant neoplasm of interlobular bile ducts
cancer	B151200	Malignant neoplasm of intrahepatic biliary passages
cancer	B151400	Malignant neoplasm of intrahepatic gall duct
cancer	B151z00	Malignant neoplasm of intrahepatic bile ducts NOS
cancer	B152.00	Malignant neoplasm of liver unspecified
cancer	B15z.00	Malignant neoplasm of liver and intrahepatic bile ducts
cancer	B16..00	Malignant neoplasm gallbladder/extrahepatic bile ducts
cancer	B160.00	Malignant neoplasm of gallbladder
cancer	B160.11	Carcinoma gallbladder
cancer	B161.00	Malignant neoplasm of extrahepatic bile ducts
cancer	B161000	Malignant neoplasm of cystic duct
cancer	B161100	Malignant neoplasm of hepatic duct
cancer	B161200	Malignant neoplasm of common bile duct
cancer	B161211	Carcinoma common bile duct
cancer	B161300	Malignant neoplasm of sphincter of Oddi
cancer	B161z00	Malignant neoplasm of extrahepatic bile ducts NOS
cancer	B162.00	Malignant neoplasm of ampulla of Vater
cancer	B163.00	Malignant neoplasm, overlapping lesion of biliary tract
cancer	B16y.00	Malignant neopl other gallbladder/extrahepatic bile duct
cancer	B16z.00	Malignant neoplasm gallbladder/extrahepatic bile ducts
cancer	B17..00	Malignant neoplasm of pancreas
cancer	B170.00	Malignant neoplasm of head of pancreas
cancer	B171.00	Malignant neoplasm of body of pancreas
cancer	B172.00	Malignant neoplasm of tail of pancreas
cancer	B173.00	Malignant neoplasm of pancreatic duct
cancer	B174.00	Malignant neoplasm of Islets of Langerhans
cancer	B175.00	Malignant neoplasm, overlapping lesion of pancreas
cancer	B17y.00	Malignant neoplasm of other specified sites of pancreas
cancer	B17y000	Malignant neoplasm of ectopic pancreatic tissue
cancer	B17yz00	Malignant neoplasm of specified site of pancreas NOS
cancer	B17z.00	Malignant neoplasm of pancreas NOS
cancer	B18..00	Malignant neoplasm of retroperitoneum and peritoneum
cancer	B180.00	Malignant neoplasm of retroperitoneum
cancer	B180100	Malignant neoplasm of perinephric tissue
cancer	B180200	Malignant neoplasm of retrocaecal tissue
cancer	B180z00	Malignant neoplasm of retroperitoneum NOS
cancer	B181.00	Mesothelioma of peritoneum
cancer	B182.00	Overlapping malign lesion retroperitoneum/ peritoneum
cancer	B18y.00	Malignant neoplasm of specified parts of peritoneum
cancer	B18y100	Malignant neoplasm of mesocaecum
cancer	B18y200	Malignant neoplasm of mesorectum

cancer	B18y300	Malignant neoplasm of omentum
cancer	B18y400	Malignant neoplasm of parietal peritoneum
cancer	B18y500	Malignant neoplasm of pelvic peritoneum
cancer	B18y600	Malignant neoplasm of the pouch of Douglas
cancer	B18y700	Malignant neoplasm of mesentery
cancer	B18yz00	Malignant neoplasm of specified parts of peritoneum NOS
cancer	B18z.00	Malignant neoplasm of retroperitoneum and peritoneum
cancer	B1z..00	Malig neop oth/illdefined sites digestive tract/peritoneum
cancer	B1z0.00	Malignant neoplasm of intestinal tract, part unspecified
cancer	B1z0.11	Cancer of bowel
cancer	B1z1.00	Malignant neoplasm of spleen NEC
cancer	B1z1000	Angiosarcoma of spleen
cancer	B1z1100	Fibrosarcoma of spleen
cancer	B1z1z00	Malignant neoplasm of spleen NOS
cancer	B1z2.00	Malignant neoplasm, overlapping lesion of digestive syst
cancer	B1zy.00	Malignant neoplasm other spec digest tract/ peritoneum
cancer	B1zz.00	Malignant neoplasm of digestive tract and peritoneum
cancer	B2...00	Malig neop of respiratory tract and intrathoracic organs
cancer	B2...11	Carcinoma of respiratory tract and intrathoracic organs
cancer	B20..00	Malig neop nasal cavities, middle ear/accessory sinuses
cancer	B200.00	Malignant neoplasm of nasal cavities
cancer	B200000	Malignant neoplasm of cartilage of nose
cancer	B200100	Malignant neoplasm of nasal conchae
cancer	B200200	Malignant neoplasm of septum of nose
cancer	B200300	Malignant neoplasm of vestibule of nose
cancer	B200z00	Malignant neoplasm of nasal cavities NOS
cancer	B201.00	Malig neop auditory tube, middle ear and mastoid air cells
cancer	B201000	Malignant neoplasm of auditory (Eustachian) tube
cancer	B201100	Malignant neoplasm of tympanic cavity
cancer	B201200	Malignant neoplasm of tympanic antrum
cancer	B201300	Malignant neoplasm of mastoid air cells
cancer	B201z00	Malig neop auditory tube, middle ear, mastoid air cells
cancer	B202.00	Malignant neoplasm of maxillary sinus
cancer	B203.00	Malignant neoplasm of ethmoid sinus
cancer	B204.00	Malignant neoplasm of frontal sinus
cancer	B205.00	Malignant neoplasm of sphenoidal sinus
cancer	B206.00	Malignant neopl, overlapping lesion of accessory sinuses
cancer	B20y.00	Malig neop other site nasal cavity, middle ear and sinuses
cancer	B20z.00	Malignant neoplasm of accessory sinus NOS
cancer	B21..00	Malignant neoplasm of larynx
cancer	B210.00	Malignant neoplasm of glottis
cancer	B211.00	Malignant neoplasm of supraglottis
cancer	B212.00	Malignant neoplasm of subglottis
cancer	B213.00	Malignant neoplasm of laryngeal cartilage

cancer	B213000	Malignant neoplasm of arytenoid cartilage
cancer	B213100	Malignant neoplasm of cricoid cartilage
cancer	B213200	Malignant neoplasm of cuneiform cartilage
cancer	B213300	Malignant neoplasm of thyroid cartilage
cancer	B213z00	Malignant neoplasm of laryngeal cartilage NOS
cancer	B214.00	Malignant neoplasm, overlapping lesion of larynx
cancer	B215.00	Malignant neoplasm of epiglottis NOS
cancer	B21y.00	Malignant neoplasm of larynx, other specified site
cancer	B21z.00	Malignant neoplasm of larynx NOS
cancer	B22..00	Malignant neoplasm of trachea, bronchus and lung
cancer	B220.00	Malignant neoplasm of trachea
cancer	B220100	Malignant neoplasm of mucosa of trachea
cancer	B220z00	Malignant neoplasm of trachea NOS
cancer	B221.00	Malignant neoplasm of main bronchus
cancer	B221000	Malignant neoplasm of carina of bronchus
cancer	B221100	Malignant neoplasm of hilus of lung
cancer	B221z00	Malignant neoplasm of main bronchus NOS
cancer	B222.00	Malignant neoplasm of upper lobe, bronchus or lung
cancer	B222.11	Pancoast's synde
cancer	B222000	Malignant neoplasm of upper lobe bronchus
cancer	B222100	Malignant neoplasm of upper lobe of lung
cancer	B222z00	Malignant neoplasm of upper lobe, bronchus or lung NOS
cancer	B223.00	Malignant neoplasm of middle lobe, bronchus or lung
cancer	B223000	Malignant neoplasm of middle lobe bronchus
cancer	B223100	Malignant neoplasm of middle lobe of lung
cancer	B223z00	Malignant neoplasm of middle lobe, bronchus or lung
cancer	B224.00	Malignant neoplasm of lower lobe, bronchus or lung
cancer	B224000	Malignant neoplasm of lower lobe bronchus
cancer	B224100	Malignant neoplasm of lower lobe of lung
cancer	B224z00	Malignant neoplasm of lower lobe, bronchus or lung NOS
cancer	B225.00	Malignant neopl of overlapping lesion of bronchus/lung
cancer	B226.00	Mesothelioma
cancer	B22y.00	Malignant neoplasm of other sites of bronchus or lung
cancer	B22z.00	Malignant neoplasm of bronchus or lung NOS
cancer	B22z.11	Lung cancer
cancer	B23..00	Malignant neoplasm of pleura
cancer	B230.00	Malignant neoplasm of parietal pleura
cancer	B231.00	Malignant neoplasm of visceral pleura
cancer	B232.00	Mesothelioma of pleura
cancer	B23y.00	Malignant neoplasm of other specified pleura
cancer	B23z.00	Malignant neoplasm of pleura NOS
cancer	B24..00	Malignant neoplasm of thymus, heart and mediastinum
cancer	B240.00	Malignant neoplasm of thymus
cancer	B241.00	Malignant neoplasm of heart

cancer	B241000	Malignant neoplasm of endocardium
cancer	B241200	Malignant neoplasm of myocardium
cancer	B241300	Malignant neoplasm of pericardium
cancer	B241400	Mesothelioma of pericardium
cancer	B241z00	Malignant neoplasm of heart NOS
cancer	B242.00	Malignant neoplasm of anterior mediastinum
cancer	B243.00	Malignant neoplasm of posterior mediastinum
cancer	B24X.00	Malignant neoplasm of mediastinum, part unspecified
cancer	B24y.00	Malig neop of other site of heart, thymus/mediastinum
cancer	B24z.00	Malignant neoplasm of heart, thymus and mediastinum
cancer	B25..00	Malig neo, overlapp lesion of heart, mediastinum/ pleura
cancer	B26..00	Malignant neoplasm, overlap lesion of resp/intrathor orgs
cancer	B2z..00	Malig neop /illdefined sites resp/intrathoracic organs
cancer	B2z0.00	Malig neop of upper respiratory tract, part unspecified
cancer	B2zy.00	Malignant neoplasm of other site of respiratory tract
cancer	B2zz.00	Malignant neoplasm of respiratory tract NOS
cancer	B3...00	Malig neop of bone, connective tissue, skin and breast
cancer	B3...11	Carcinoma of bone, connective tissue, skin and breast
cancer	B3...12	Sarcoma of bone and connective tissue
cancer	B30..00	Malignant neoplasm of bone and articular cartilage
cancer	B300.00	Malignant neoplasm of bones of skull and face
cancer	B300000	Malignant neoplasm of ethmoid bone
cancer	B300100	Malignant neoplasm of frontal bone
cancer	B300200	Malignant neoplasm of malar bone
cancer	B300300	Malignant neoplasm of nasal bone
cancer	B300400	Malignant neoplasm of occipital bone
cancer	B300500	Malignant neoplasm of orbital bone
cancer	B300600	Malignant neoplasm of parietal bone
cancer	B300700	Malignant neoplasm of sphenoid bone
cancer	B300800	Malignant neoplasm of temporal bone
cancer	B300900	Malignant neoplasm of zygomatic bone
cancer	B300A00	Malignant neoplasm of maxilla
cancer	B300B00	Malignant neoplasm of turbinate
cancer	B300C00	Malignant neoplasm of vomer
cancer	B300z00	Malignant neoplasm of bones of skull and face NOS
cancer	B301.00	Malignant neoplasm of mandible
cancer	B302.00	Malignant neoplasm of vertebral column
cancer	B302000	Malignant neoplasm of cervical vertebra
cancer	B302100	Malignant neoplasm of thoracic vertebra
cancer	B302200	Malignant neoplasm of lumbar vertebra
cancer	B302z00	Malignant neoplasm of vertebral column NOS
cancer	B303.00	Malignant neoplasm of ribs, sternum and clavicle
cancer	B303000	Malignant neoplasm of rib
cancer	B303100	Malignant neoplasm of sternum

cancer	B303200	Malignant neoplasm of clavicle
cancer	B303300	Malignant neoplasm of costal cartilage
cancer	B303400	Malignant neoplasm of costo-vertebral joint
cancer	B303500	Malignant neoplasm of xiphoid process
cancer	B303z00	Malignant neoplasm of rib, sternum and clavicle NOS
cancer	B304.00	Malignant neoplasm of scapula and long bones upper arm
cancer	B304000	Malignant neoplasm of scapula
cancer	B304100	Malignant neoplasm of acromion
cancer	B304200	Malignant neoplasm of humerus
cancer	B304300	Malignant neoplasm of radius
cancer	B304400	Malignant neoplasm of ulna
cancer	B304z00	Malig neop of scapula and long bones of upper arm NOS
cancer	B305.00	Malignant neoplasm of hand bones
cancer	B305.11	Malignant neoplasm of carpal bones
cancer	B305.12	Malignant neoplasm of metacarpal bones
cancer	B305000	Malignant neoplasm of carpal bone - scaphoid
cancer	B305100	Malignant neoplasm of carpal bone - lunate
cancer	B305A00	Malignant neoplasm of third metacarpal bone
cancer	B305C00	Malignant neoplasm of fifth metacarpal bone
cancer	B305D00	Malignant neoplasm of phalanges of hand
cancer	B305z00	Malignant neoplasm of hand bones NOS
cancer	B306.00	Malignant neoplasm of pelvic bones, sacrum and coccyx
cancer	B306000	Malignant neoplasm of ilium
cancer	B306100	Malignant neoplasm of ischium
cancer	B306200	Malignant neoplasm of pubis
cancer	B306300	Malignant neoplasm of sacral vertebra
cancer	B306400	Malignant neoplasm of coccygeal vertebra
cancer	B306500	Malignant sacral teratoma
cancer	B306z00	Malignant neoplasm of pelvis, sacrum or coccyx NOS
cancer	B307.00	Malignant neoplasm of long bones of leg
cancer	B307000	Malignant neoplasm of femur
cancer	B307100	Malignant neoplasm of fibula
cancer	B307200	Malignant neoplasm of tibia
cancer	B307z00	Malignant neoplasm of long bones of leg NOS
cancer	B308.00	Malignant neoplasm of short bones of leg
cancer	B308100	Malignant neoplasm of talus
cancer	B308200	Malignant neoplasm of calcaneum
cancer	B308300	Malignant neoplasm of medial cuneiform
cancer	B308800	Malignant neoplasm of first metatarsal bone
cancer	B308B00	Malignant neoplasm of fourth metatarsal bone
cancer	B308D00	Malignant neoplasm of phalanges of foot
cancer	B308z00	Malignant neoplasm of short bones of leg NOS
cancer	B30W.00	Malignant neopl/overlap lesion/bone+articulr cartilage
cancer	B30X.00	Malignant neopl/bones+articular cartilage/limb,unspfd

cancer	B30z.00	Malignant neoplasm of bone and articular cartilage NOS
cancer	B30z000	Osteosarcoma
cancer	B31..00	Malignant neoplasm of connective and other soft tissue
cancer	B310.00	Malig neop of connective and soft tissue head, face/ neck
cancer	B310000	Malignant neoplasm of soft tissue of head
cancer	B310100	Malignant neoplasm of soft tissue of face
cancer	B310200	Malignant neoplasm of soft tissue of neck
cancer	B310300	Malignant neoplasm of cartilage of ear
cancer	B310400	Malignant neoplasm of tarsus of eyelid
cancer	B310500	Malignant neoplasm soft tissues of cervical spine
cancer	B310z00	Malig neop connective and soft tissue head, face, neck
cancer	B311.00	Malig neop connective soft tissue upper limb/shoulder
cancer	B311000	Malignant neoplasm of connective soft tissue of shoulder
cancer	B311100	Malignant neoplasm of connective soft tissue, upper arm
cancer	B311200	Malignant neoplasm of connective soft tissue of fore-arm
cancer	B311300	Malignant neoplasm of connective and soft tissue of hand
cancer	B311400	Malignant neoplasm of connective soft tissue of finger
cancer	B311500	Malignant neoplasm of connective soft tissue of thumb
cancer	B311z00	Malig neop connective soft tissue upper limb/shoulder
cancer	B312.00	Malig neop of connective and soft tissue of hip and leg
cancer	B312000	Malignant neoplasm of connective and soft tissue of hip
cancer	B312100	Malig neop of connective and soft tissue thigh upper leg
cancer	B312200	Malig neop connective and soft tissue of popliteal space
cancer	B312300	Malig neop of connective and soft tissue of lower leg
cancer	B312400	Malignant neoplasm of connective and soft tissue of foot
cancer	B312500	Malignant neoplasm of connective and soft tissue of toe
cancer	B312z00	Malig neop connective and soft tissue hip and leg NOS
cancer	B313.00	Malignant neoplasm of connective and soft tissue of thorax
cancer	B313000	Malignant neoplasm of connective and soft tissue of axilla
cancer	B313100	Malignant neoplasm of diaphragm
cancer	B313200	Malignant neoplasm of great vessels
cancer	B313300	Malig neoplasm of connective soft tissues of thor spine
cancer	B313z00	Malig neop of connective and soft tissue of thorax NOS
cancer	B314.00	Malignant neoplasm of connective soft tissue of abdomen
cancer	B314000	Malig neop of connective soft tissue of abdominal wall
cancer	B314100	Malig neoplasm of connective soft tissues of lumb spine
cancer	B314z00	Malig neop of connective and soft tissue of abdomen NOS
cancer	B315.00	Malignant neoplasm of connective soft tissue of pelvis
cancer	B315000	Malignant neoplasm of connective soft tissue of buttock
cancer	B315100	Malig neop of connective soft tissue of inguinal region
cancer	B315200	Malignant neoplasm of connective soft tissue of perineum
cancer	B315z00	Malig neop of connective and soft tissue of pelvis NOS
cancer	B316.00	Malig neop of connective and soft tissue trunk unspfied
cancer	B31y.00	Malig neop connective and soft tissue other specified site

cancer	B31z.00	Malignant neoplasm of connective and soft tissue, site
cancer	B31z000	Kaposi's sarcoma of soft tissue
cancer	B32..00	Malignant melanoma of skin
cancer	B320.00	Malignant melanoma of lip
cancer	B321.00	Malignant melanoma of eyelid including canthus
cancer	B322.00	Malignant melanoma of ear and external auricular canal
cancer	B322000	Malignant melanoma of auricle (ear)
cancer	B322100	Malignant melanoma of external auditory meatus
cancer	B322z00	Malignant melanoma of ear and external auricular canal
cancer	B323.00	Malignant melanoma of other unspecified parts of face
cancer	B323000	Malignant melanoma of external surface of cheek
cancer	B323100	Malignant melanoma of chin
cancer	B323200	Malignant melanoma of eyebrow
cancer	B323300	Malignant melanoma of forehead
cancer	B323400	Malignant melanoma of external surface of nose
cancer	B323500	Malignant melanoma of temple
cancer	B323z00	Malignant melanoma of face NOS
cancer	B324.00	Malignant melanoma of scalp and neck
cancer	B324000	Malignant melanoma of scalp
cancer	B324100	Malignant melanoma of neck
cancer	B324z00	Malignant melanoma of scalp and neck NOS
cancer	B325.00	Malignant melanoma of trunk (excluding scrotum)
cancer	B325000	Malignant melanoma of axilla
cancer	B325100	Malignant melanoma of breast
cancer	B325200	Malignant melanoma of buttock
cancer	B325300	Malignant melanoma of groin
cancer	B325400	Malignant melanoma of perianal skin
cancer	B325500	Malignant melanoma of perineum
cancer	B325600	Malignant melanoma of umbilicus
cancer	B325700	Malignant melanoma of back
cancer	B325800	Malignant melanoma of chest wall
cancer	B325z00	Malignant melanoma of trunk, excluding scrotum, NOS
cancer	B326.00	Malignant melanoma of upper limb and shoulder
cancer	B326000	Malignant melanoma of shoulder
cancer	B326100	Malignant melanoma of upper arm
cancer	B326200	Malignant melanoma of fore-arm
cancer	B326300	Malignant melanoma of hand
cancer	B326400	Malignant melanoma of finger
cancer	B326500	Malignant melanoma of thumb
cancer	B326z00	Malignant melanoma of upper limb or shoulder NOS
cancer	B327.00	Malignant melanoma of lower limb and hip
cancer	B327000	Malignant melanoma of hip
cancer	B327100	Malignant melanoma of thigh
cancer	B327200	Malignant melanoma of knee

cancer	B327300	Malignant melanoma of popliteal fossa area
cancer	B327400	Malignant melanoma of lower leg
cancer	B327500	Malignant melanoma of ankle
cancer	B327600	Malignant melanoma of heel
cancer	B327700	Malignant melanoma of foot
cancer	B327800	Malignant melanoma of toe
cancer	B327900	Malignant melanoma of great toe
cancer	B327z00	Malignant melanoma of lower limb or hip NOS
cancer	B32y.00	Malignant melanoma of other specified skin site
cancer	B32y000	Overlapping malignant melanoma of skin
cancer	B32z.00	Malignant melanoma of skin NOS
cancer	B339.00	Dermatofibrosarcoma protuberans
cancer	B34..00	Malignant neoplasm of female breast
cancer	B34..11	Ca female breast
cancer	B340.00	Malignant neoplasm of nipple and areola of female breast
cancer	B340000	Malignant neoplasm of nipple of female breast
cancer	B340100	Malignant neoplasm of areola of female breast
cancer	B340z00	Malignant neoplasm of nipple or areola of female breast
cancer	B341.00	Malignant neoplasm of central part of female breast
cancer	B342.00	Malignant neoplasm upperinner quadrant female breast
cancer	B343.00	Malignant neoplasm lower-inner quadrant female breast
cancer	B344.00	Malignant neopl of upperouter quadrant female breast
cancer	B345.00	Malignant neopl of lower-outer quadrant female breast
cancer	B346.00	Malignant neoplasm of axillary tail of female breast
cancer	B347.00	Malignant neoplasm, overlapping lesion of breast
cancer	B34y.00	Malignant neoplasm of other site of female breast
cancer	B34y000	Malignant neoplasm of ectopic site of female breast
cancer	B34yz00	Malignant neoplasm of other site of female breast NOS
cancer	B34z.00	Malignant neoplasm of female breast NOS
cancer	B35..00	Malignant neoplasm of male breast
cancer	B350.00	Malignant neoplasm of nipple and areola of male breast
cancer	B350000	Malignant neoplasm of nipple of male breast
cancer	B350100	Malignant neoplasm of areola of male breast
cancer	B35z.00	Malignant neoplasm of other site of male breast
cancer	B35z000	Malignant neoplasm of ectopic site of male breast
cancer	B35zz00	Malignant neoplasm of male breast NOS
cancer	B36..00	Local recurrence of malignant tumour of breast
cancer	B3y..00	Malig neop of bone, connective tissue, skin and breast OS
cancer	B3z..00	Malig neop of bone, connective tissue, skin and breast
cancer	B4...00	Malignant neoplasm of genitourinary organ
cancer	B4...11	Carcinoma of genitourinary organ
cancer	B40..00	Malignant neoplasm of uterus, part unspecified
cancer	B41..00	Malignant neoplasm of cervix uteri
cancer	B41..11	Cervical carcinoma (uterus)

cancer	B410.00	Malignant neoplasm of endocervix
cancer	B410000	Malignant neoplasm of endocervical canal
cancer	B410100	Malignant neoplasm of endocervical gland
cancer	B410z00	Malignant neoplasm of endocervix NOS
cancer	B411.00	Malignant neoplasm of exocervix
cancer	B412.00	Malignant neoplasm, overlapping lesion of cervix uteri
cancer	B41y.00	Malignant neoplasm of other site of cervix
cancer	B41y000	Malignant neoplasm of cervical stump
cancer	B41y100	Malignant neoplasm of squamocolumnar junction cervix
cancer	B41yz00	Malignant neoplasm of other site of cervix NOS
cancer	B41z.00	Malignant neoplasm of cervix uteri NOS
cancer	B42..00	Malignant neoplasm of placenta
cancer	B420.00	Choriocarcinoma
cancer	B43..00	Malignant neoplasm of body of uterus
cancer	B430.00	Malignant neoplasm of corpus uteri, excluding isthmus
cancer	B430000	Malignant neoplasm of cornu of corpus uteri
cancer	B430100	Malignant neoplasm of fundus of corpus uteri
cancer	B430200	Malignant neoplasm of endometrium of corpus uteri
cancer	B430211	Malignant neoplasm of endometrium
cancer	B430300	Malignant neoplasm of myometrium of corpus uteri
cancer	B430z00	Malignant neoplasm of corpus uteri NOS
cancer	B431.00	Malignant neoplasm of isthmus of uterine body
cancer	B431000	Malignant neoplasm of lower uterine segment
cancer	B431z00	Malignant neoplasm of isthmus of uterine body NOS
cancer	B432.00	Malignant neoplasm of overlapping lesion of corpus uteri
cancer	B43y.00	Malignant neoplasm of other site of uterine body
cancer	B43z.00	Malignant neoplasm of body of uterus NOS
cancer	B44..00	Malignant neoplasm of ovary and other uterine adnexa
cancer	B440.00	Malignant neoplasm of ovary
cancer	B440.11	Cancer of ovary
cancer	B441.00	Malignant neoplasm of fallopian tube
cancer	B442.00	Malignant neoplasm of broad ligament
cancer	B443.00	Malignant neoplasm of parametrium
cancer	B44y.00	Malignant neoplasm of other site of uterine adnexa
cancer	B44z.00	Malignant neoplasm of uterine adnexa NOS
cancer	B45..00	Malig neop of other unspecified female genital organs
cancer	B450.00	Malignant neoplasm of vagina
cancer	B450100	Malignant neoplasm of vaginal vault
cancer	B450z00	Malignant neoplasm of vagina NOS
cancer	B451.00	Malignant neoplasm of labia majora
cancer	B451000	Malignant neoplasm greater vestibular (Bartholin's) gland
cancer	B451z00	Malignant neoplasm of labia majora NOS
cancer	B452.00	Malignant neoplasm of labia minora
cancer	B453.00	Malignant neoplasm of clitoris

cancer	B454.00	Malignant neoplasm of vulva unspecified
cancer	B454.11	Primary vulval cancer
cancer	B45X.00	Malignant neopl/overlapping lesion/feml genital organs
cancer	B45y.00	Malignant neoplasm other specified female genital organ
cancer	B45y000	Malignant neoplasm of overlapping lesion of vulva
cancer	B45z.00	Malignant neoplasm of female genital organ NOS
cancer	B46..00	Malignant neoplasm of prostate
cancer	B47..00	Malignant neoplasm of testis
cancer	B470.00	Malignant neoplasm of undescended testis
cancer	B470200	Seminoma of undescended testis
cancer	B470300	Teratoma of undescended testis
cancer	B470z00	Malignant neoplasm of undescended testis NOS
cancer	B471.00	Malignant neoplasm of descended testis
cancer	B471000	Seminoma of descended testis
cancer	B471100	Teratoma of descended testis
cancer	B471z00	Malignant neoplasm of descended testis NOS
cancer	B47z.00	Malignant neoplasm of testis NOS
cancer	B47z.11	Seminoma of testis
cancer	B47z.12	Teratoma of testis
cancer	B48..00	Malignant neoplasm of penis other male genital organs
cancer	B480.00	Malignant neoplasm of prepuce (foreskin)
cancer	B481.00	Malignant neoplasm of glans penis
cancer	B482.00	Malignant neoplasm of body of penis
cancer	B483.00	Malignant neoplasm of penis, part unspecified
cancer	B484.00	Malignant neoplasm of epididymis
cancer	B485.00	Malignant neoplasm of spermatic cord
cancer	B486.00	Malignant neoplasm of scrotum
cancer	B487.00	Malignant neoplasm, overlapping lesion of penis
cancer	B48y.00	Malignant neoplasm of other male genital organ
cancer	B48y000	Malignant neoplasm of seminal vesicle
cancer	B48y100	Malignant neoplasm of tunica vaginalis
cancer	B48y200	Malignant neoplasm, overlapping lesion male genital orgs
cancer	B48yz00	Malignant neoplasm of other male genital organ NOS
cancer	B48z.00	Malignant neoplasm of penis other male genital organ
cancer	B49..00	Malignant neoplasm of urinary bladder
cancer	B490.00	Malignant neoplasm of trigone of urinary bladder
cancer	B491.00	Malignant neoplasm of dome of urinary bladder
cancer	B492.00	Malignant neoplasm of lateral wall of urinary bladder
cancer	B493.00	Malignant neoplasm of anterior wall of urinary bladder
cancer	B494.00	Malignant neoplasm of posterior wall of urinary bladder
cancer	B495.00	Malignant neoplasm of bladder neck
cancer	B496.00	Malignant neoplasm of ureteric orifice
cancer	B497.00	Malignant neoplasm of urachus
cancer	B498.00	Local recurrence of malignant tumour of urinary bladder

cancer	B49y.00	Malignant neoplasm of other site of urinary bladder
cancer	B49y000	Malignant neoplasm, overlapping lesion of bladder
cancer	B49z.00	Malignant neoplasm of urinary bladder NOS
cancer	B4A..00	Malig neop of kidney other unspecified urinary organs
cancer	B4A..11	Renal malignant neoplasm
cancer	B4A0.00	Malignant neoplasm of kidney parenchyma
cancer	B4A0000	Hypernephroma
cancer	B4A1.00	Malignant neoplasm of renal pelvis
cancer	B4A1000	Malignant neoplasm of renal calyces
cancer	B4A1100	Malignant neoplasm of ureteropelvic junction
cancer	B4A1z00	Malignant neoplasm of renal pelvis NOS
cancer	B4A2.00	Malignant neoplasm of ureter
cancer	B4A3.00	Malignant neoplasm of urethra
cancer	B4A4.00	Malignant neoplasm of paraurethral glands
cancer	B4Ay.00	Malignant neoplasm of other urinary organs
cancer	B4Ay000	Malignant neoplasm overlapping lesion of urinary organs
cancer	B4Az.00	Malignant neoplasm of kidney or urinary organs NOS
cancer	B4y..00	Malignant neoplasm of genitourinary organ OS
cancer	B4z..00	Malignant neoplasm of genitourinary organ NOS
cancer	B5...00	Malignant neoplasm of other and unspecified sites
cancer	B5...11	Carcinoma of other and unspecified sites
cancer	B50..00	Malignant neoplasm of eye
cancer	B500.00	Malig neop eyeball exc conjctiva, cornea, retina, choroid
cancer	B500000	Malignant neoplasm of ciliary body
cancer	B500100	Malignant neoplasm of iris
cancer	B500200	Malignant neoplasm of crystalline lens
cancer	B500z00	Malignant neoplasm of eyeball NOS
cancer	B501.00	Malignant neoplasm of orbit
cancer	B501000	Malignant neoplasm of connective tissue of orbit
cancer	B501z00	Malignant neoplasm of orbit NOS
cancer	B502.00	Malignant neoplasm of lacrimal gland
cancer	B503.00	Malignant neoplasm of conjunctiva
cancer	B504.00	Malignant neoplasm of cornea
cancer	B505.00	Malignant neoplasm of retina
cancer	B506.00	Malignant neoplasm of choroid
cancer	B507.00	Malignant neoplasm of lacrimal duct
cancer	B507000	Malignant neoplasm of lacrimal sac
cancer	B507100	Malignant neoplasm of nasolacrimal duct
cancer	B508.00	Malignant neoplasm, overlapping lesion of eye adnexa
cancer	B509.00	Malignant melanoma of eye
cancer	B50y.00	Malignant neoplasm of other specified site of eye
cancer	B50z.00	Malignant neoplasm of eye NOS
cancer	B51..00	Malignant neoplasm of brain
cancer	B51..11	Cerebral tumour - malignant

cancer	B510.00	Malignant neoplasm cerebrum (excl lobes, ventricles)
cancer	B510000	Malignant neoplasm of basal ganglia
cancer	B510100	Malignant neoplasm of cerebral cortex
cancer	B510300	Malignant neoplasm of globus pallidus
cancer	B510400	Malignant neoplasm of hypothalamus
cancer	B510500	Malignant neoplasm of thalamus
cancer	B510z00	Malignant neoplasm of cerebrum NOS
cancer	B511.00	Malignant neoplasm of frontal lobe
cancer	B512.00	Malignant neoplasm of temporal lobe
cancer	B512000	Malignant neoplasm of hippocampus
cancer	B512z00	Malignant neoplasm of temporal lobe NOS
cancer	B513.00	Malignant neoplasm of parietal lobe
cancer	B514.00	Malignant neoplasm of occipital lobe
cancer	B515.00	Malignant neoplasm of cerebral ventricles
cancer	B515000	Malignant neoplasm of choroid plexus
cancer	B516.00	Malignant neoplasm of cerebellum
cancer	B517.00	Malignant neoplasm of brain stem
cancer	B517000	Malignant neoplasm of cerebral peduncle
cancer	B517100	Malignant neoplasm of medulla oblongata
cancer	B517200	Malignant neoplasm of midbrain
cancer	B517300	Malignant neoplasm of pons
cancer	B517z00	Malignant neoplasm of brain stem NOS
cancer	B51y.00	Malignant neoplasm of other parts of brain
cancer	B51y000	Malignant neoplasm of corpus callosum
cancer	B51y200	Malignant neoplasm, overlapping lesion of brain
cancer	B51yz00	Malignant neoplasm of other part of brain NOS
cancer	B51z.00	Malignant neoplasm of brain NOS
cancer	B52..00	Malig neop of other unspecified parts of nervous system
cancer	B520.00	Malignant neoplasm of cranial nerves
cancer	B520000	Malignant neoplasm of olfactory bulb
cancer	B520100	Malignant neoplasm of optic nerve
cancer	B520200	Malignant neoplasm of acoustic nerve
cancer	B520z00	Malignant neoplasm of cranial nerves NOS
cancer	B521.00	Malignant neoplasm of cerebral meninges
cancer	B521z00	Malignant neoplasm of cerebral meninges NOS
cancer	B522.00	Malignant neoplasm of spinal cord
cancer	B523.00	Malignant neoplasm of spinal meninges
cancer	B523z00	Malignant neoplasm of spinal meninges NOS
cancer	B524.00	Malig neopl peripheral nerves autonomic nervous system
cancer	B524000	Malignant neoplasm peripheral nerves of head, face neck
cancer	B524100	Malign neoplasm peripheral nerve,upp limb,incl should
cancer	B524200	Malign neoplasm of peripheral nerve of low limb, incl hip
cancer	B524300	Malignant neoplasm of peripheral nerve of thorax
cancer	B524400	Malignant neoplasm of peripheral nerve of abdomen

cancer	B524500	Malignant neoplasm of peripheral nerve of pelvis
cancer	B524600	Malignant neoplasm,overlap lesion periph nerve/auton ns
cancer	B524W00	Mal neoplasm/periph nerves+autonomic nervous system
cancer	B525.00	Malignant neoplasm of cauda equina
cancer	B52W.00	Malig neopl, overlap lesion brain & other part of CNS
cancer	B52X.00	Malignant neoplasm of meninges, unspecified
cancer	B52y.00	Malignant neoplasm of other specified part of nervous sys
cancer	B52z.00	Malignant neoplasm of nervous system NOS
cancer	B53..00	Malignant neoplasm of thyroid gland
cancer	B54..00	Malig neop of other endocrine glands/related structures
cancer	B540.00	Malignant neoplasm of adrenal gland
cancer	B540.11	Phaeochromocytoma
cancer	B540000	Malignant neoplasm of adrenal cortex
cancer	B540100	Malignant neoplasm of adrenal medulla
cancer	B540z00	Malignant neoplasm of adrenal gland NOS
cancer	B541.00	Malignant neoplasm of parathyroid gland
cancer	B542.00	Malignant neopl pituitary gland/craniopharyngeal duct
cancer	B542000	Malignant neoplasm of pituitary gland
cancer	B542100	Malignant neoplasm of craniopharyngeal duct
cancer	B542z00	Malig neop pituitary gland or craniopharyngeal duct NOS
cancer	B543.00	Malignant neoplasm of pineal gland
cancer	B544.00	Malignant neoplasm of carotid body
cancer	B545.00	Malignant neoplasm of aortic body and other paraganglia
cancer	B545000	Malignant neoplasm of glomus jugulare
cancer	B545100	Malignant neoplasm of aortic body
cancer	B545200	Malignant neoplasm of coccygeal body
cancer	B545z00	Malignant neoplasm of aortic body or paraganglia NOS
cancer	B546.00	Neuroblastoma
cancer	B54X.00	Malignant neoplasm-pluriglandular involvement,unspecified
cancer	B54y.00	Malignant neoplasm of other specified endocrine gland
cancer	B54z.00	Malig neop of endocrine gland or related structure NOS
cancer	B55..00	Malignant neoplasm of other and ill-defined sites
cancer	B550.00	Malignant neoplasm of head, neck and face
cancer	B550000	Malignant neoplasm of head NOS
cancer	B550100	Malignant neoplasm of cheek NOS
cancer	B550200	Malignant neoplasm of nose NOS
cancer	B550300	Malignant neoplasm of jaw NOS
cancer	B550400	Malignant neoplasm of neck NOS
cancer	B550500	Malignant neoplasm of supraclavicular fossa NOS
cancer	B550z00	Malignant neoplasm of head, neck and face NOS
cancer	B551.00	Malignant neoplasm of thorax
cancer	B551000	Malignant neoplasm of axilla NOS
cancer	B551100	Malignant neoplasm of chest wall NOS
cancer	B551200	Malignant neoplasm of intrathoracic site NOS

cancer	B551z00	Malignant neoplasm of thorax NOS
cancer	B552.00	Malignant neoplasm of abdomen
cancer	B553.00	Malignant neoplasm of pelvis
cancer	B553000	Malignant neoplasm of inguinal region NOS
cancer	B553100	Malignant neoplasm of presacral region
cancer	B553200	Malignant neoplasm of sacrococcygeal region
cancer	B553z00	Malignant neoplasm of pelvis NOS
cancer	B554.00	Malignant neoplasm of upper limb NOS
cancer	B555.00	Malignant neoplasm of lower limb NOS
cancer	B55y.00	Malignant neoplasm of other specified sites
cancer	B55y000	Malignant neoplasm of back NOS
cancer	B55y100	Malignant neoplasm of trunk NOS
cancer	B55y200	Malignant neoplasm of flank NOS
cancer	B55yz00	Malignant neoplasm of specified site NOS
cancer	B55z.00	Malignant neoplasm of other and ill defined site NOS
cancer	B59..00	Malignant neoplasm of unspecified site
cancer	B590.00	Disseminated malignancy NOS
cancer	B590.11	Carcinomatosis
cancer	B591.00	Other malignant neoplasm NOS
cancer	B592.00	Malignant neopl of independent (primary) multiple sites
cancer	B592X00	Kaposi's sarcoma of multiple organs
cancer	B593.00	Primary malignant neoplasm of unknown site
cancer	B595.00	Malignant tumour of unknown origin
cancer	B59z.00	Malignant neoplasm of unspecified site NOS
cancer	B59zX00	Kaposi's sarcoma, unspecified
cancer	B5y..00	Malignant neoplasm of other and unspecified site OS
cancer	B5z..00	Malignant neoplasm of other and unspecified site NOS
cancer	B6...00	Malignant neoplasm of lymphatic/haemopoietic tissue
cancer	B6...11	Malignant neoplasm of histiocytic tissue
cancer	B60..00	Lymphosarcoma and reticulosarcoma
cancer	B600.00	Reticulosarcoma
cancer	B600000	Reticulosarcoma of unspecified site
cancer	B600100	Reticulosarcoma of lymph nodes of head, face and neck
cancer	B600300	Reticulosarcoma of intra-abdominal lymph nodes
cancer	B600700	Reticulosarcoma of spleen
cancer	B600z00	Reticulosarcoma NOS
cancer	B601.00	Lymphosarcoma
cancer	B601000	Lymphosarcoma of unspecified site
cancer	B601100	Lymphosarcoma of lymph nodes of head, face and neck
cancer	B601200	Lymphosarcoma of intrathoracic lymph nodes
cancer	B601300	Lymphosarcoma of intra-abdominal lymph nodes
cancer	B601500	Lymphosarcoma of lymph nodes inguinal region and leg
cancer	B601700	Lymphosarcoma of spleen
cancer	B601800	Lymphosarcoma of lymph nodes of multiple sites

cancer	B601z00	Lymphosarcoma NOS
cancer	B602.00	Burkitt's lymphoma
cancer	B602100	Burkitt's lymphoma lymph nodes of head, face and neck
cancer	B602200	Burkitt's lymphoma of intrathoracic lymph nodes
cancer	B602300	Burkitt's lymphoma of intra-abdominal lymph nodes
cancer	B602500	Burkitt's lymphoma lymph nodes of inguinal region/leg
cancer	B602z00	Burkitt's lymphoma NOS
cancer	B60y.00	Other specified reticulosarcoma or lymphosarcoma
cancer	B60z.00	Reticulosarcoma or lymphosarcoma NOS
cancer	B61..00	Hodgkin's dis
cancer	B61..11	Hodgkin lymphoma
cancer	B610.00	Hodgkin's paragranuloma
cancer	B610100	Hodgkin's paragranuloma lymph nodes head, face, neck
cancer	B610300	Hodgkin's paragranuloma of intra-abdominal lymph nodes
cancer	B611.00	Hodgkin's granuloma
cancer	B611100	Hodgkin's granuloma of lymph nodes head, face and neck
cancer	B612.00	Hodgkin's sarcoma
cancer	B612400	Hodgkin's sarcoma of lymph nodes axilla and upper limb
cancer	B613.00	Hodgkin's dis, lymphocytic-histiocytic predominance
cancer	B613000	Hodgkin's, lymphocytichistiocytic pred unspec site
cancer	B613100	Hodgkin's, lymphocytichistiocytic pred of head, face, neck
cancer	B613200	Hodgkin's, lymphocytichistiocytic pred intrathora nodes
cancer	B613300	Hodgkin's, lymphocytichistiocytic pred intraabdo node
cancer	B613500	Hodgkin's, lymphocytic-histiocytic pred inguinal and leg
cancer	B613600	Hodgkin's, lymphocytic-histiocytic pred intrapelvic nodes
cancer	B613700	Hodgkin's, lymphocytic-histiocytic predominance spleen
cancer	B613800	Hodgkin's, lymphocytic-histiocytic pred of multiple sites
cancer	B613z00	Hodgkin's, lymphocytic-histiocytic predominance NOS
cancer	B614.00	Hodgkin's dis, nodular sclerosis
cancer	B614000	Hodgkin's dis, nodular sclerosis of unspecified site
cancer	B614100	Hodgkin's nodular sclerosis of head, face and neck
cancer	B614200	Hodgkin's nodular sclerosis of intrathoracic lymph nodes
cancer	B614300	Hodgkin's nodular sclerosis intraabdominal lymph nodes
cancer	B614400	Hodgkin's nodular sclerosis lymph nodes of axilla and arm
cancer	B614700	Hodgkin's dis, nodular sclerosis of spleen
cancer	B614800	Hodgkin's nodular sclerosis lymph nodes of multiple sites
cancer	B614z00	Hodgkin's dis, nodular sclerosis NOS
cancer	B615.00	Hodgkin's dis, mixed cellularity
cancer	B615000	Hodgkin's dis, mixed cellularity of unspecified site
cancer	B615100	Hodgkin's mixed cellularity lymph nodes head, face, neck
cancer	B615200	Hodgkin's mixed cellularity of intrathoracic lymph nodes
cancer	B615500	Hodgkin's mixed cellularity of lymph nodes inguinal/ leg
cancer	B615z00	Hodgkin's dis, mixed cellularity NOS
cancer	B616.00	Hodgkin's dis, lymphocytic depletion

cancer	B616000	Hodgkin's lymphocytic depletion of unspecified site
cancer	B616400	Hodgkin's lymphocytic depletion lymph nodes axilla/ arm
cancer	B616700	Hodgkin's dis, lymphocytic depletion of spleen
cancer	B616800	Hodgkin's lymphocytic depletion lymph nodes mult sites
cancer	B616z00	Hodgkin's dis, lymphocytic depletion NOS
cancer	B617.00	Nodular lymphocyte predominant Hodgkin lymphoma
cancer	B618.00	Nodular sclerosis classical Hodgkin lymphoma
cancer	B619.00	Mixed cellularity classical Hodgkin lymphoma
cancer	B61B.00	Lymphocyte-rich classical Hodgkin lymphoma
cancer	B61C.00	Other classical Hodgkin lymphoma
cancer	B61z.00	Hodgkin's dis NOS
cancer	B61z.11	Hodgkin lymphoma NOS
cancer	B61z000	Hodgkin's dis NOS, unspecified site
cancer	B61z100	Hodgkin's dis NOS of lymph nodes of head, face, neck
cancer	B61z200	Hodgkin's dis NOS of intrathoracic lymph nodes
cancer	B61z300	Hodgkin's dis NOS of intra-abdominal lymph nodes
cancer	B61z400	Hodgkin's dis NOS of lymph nodes of axilla and arm
cancer	B61z500	Hodgkin's dis NOS of lymph nodes inguinal region, leg
cancer	B61z700	Hodgkin's dis NOS of spleen
cancer	B61z800	Hodgkin's dis NOS of lymph nodes of multiple sites
cancer	B61zz00	Hodgkin's dis NOS
cancer	B62..00	Other malig neoplasm of lymphoid and histiocytic tissue
cancer	B620.00	Nodular lymphoma (Brill - Symmers dis)
cancer	B620000	Nodular lymphoma of unspecified site
cancer	B620100	Nodular lymphoma of lymph nodes of head, face, neck
cancer	B620200	Nodular lymphoma of intrathoracic lymph nodes
cancer	B620300	Nodular lymphoma of intra-abdominal lymph nodes
cancer	B620500	Nodular lymphoma of lymph nodes of inguinal region, leg
cancer	B620800	Nodular lymphoma of lymph nodes of multiple sites
cancer	B620z00	Nodular lymphoma NOS
cancer	B621.00	Mycosis fungoides
cancer	B621000	Mycosis fungoides of unspecified site
cancer	B621300	Mycosis fungoides of intra-abdominal lymph nodes
cancer	B621400	Mycosis fungoides of lymph nodes of axilla, upper limb
cancer	B621500	Mycosis fungoides of lymph nodes of inguinal region leg
cancer	B621800	Mycosis fungoides of lymph nodes of multiple sites
cancer	B621z00	Mycosis fungoides NOS
cancer	B622.00	Sezary's dis
cancer	B622z00	Sezary's dis NOS
cancer	B623.00	Malignant histiocytosis
cancer	B623000	Malignant histiocytosis of unspecified site
cancer	B623100	Malignant histiocytosis of lymph nodes head, face neck
cancer	B623300	Malignant histiocytosis of intra-abdominal lymph nodes
cancer	B623z00	Malignant histiocytosis NOS

cancer	B624.00	Leukaemic reticuloendotheliosis
cancer	B624.11	Leukaemic reticuloendotheliosis
cancer	B624.12	Hairy cell leukaemia
cancer	B624000	Leukaemic reticuloendotheliosis of unspecified sites
cancer	B624300	Leukaemic reticuloend of intra-abdominal lymph nodes
cancer	B624z00	Leukaemic reticuloendotheliosis NOS
cancer	B625.00	Letterer-Siwe dis
cancer	B625.11	Histiocytosis X (acute, progressive)
cancer	B625000	Letterer-Siwe dis of unspecified sites
cancer	B625200	Letterer-Siwe dis of intrathoracic lymph nodes
cancer	B625800	Letterer-Siwe dis of lymph nodes of multiple sites
cancer	B625z00	Letterer-Siwe dis NOS
cancer	B626.00	Malignant mast cell tumours
cancer	B626000	Mast cell malignancy of unspecified site
cancer	B626500	Mast cell malignancy of lymph nodes inguinal region leg
cancer	B626800	Mast cell malignancy of lymph nodes of multiple sites
cancer	B626z00	Malignant mast cell tumour NOS
cancer	B627.00	Non - Hodgkin's lymphoma
cancer	B627.11	Non-Hodgkin lymphoma
cancer	B627000	Follicular non-Hodgkin's small cleaved cell lymphoma
cancer	B627100	Follicular non-Hodg mixed sml cleavd & lge cell lymphoma
cancer	B627200	Follicular non-Hodgkin's large cell lymphoma
cancer	B627300	Diffuse non-Hodgkin's small cell (diffuse) lymphoma
cancer	B627400	Diffuse non-Hodgkin's small cleaved cell (diffuse) lymph
cancer	B627500	Diffuse non-Hodgkin mixed sml & lge cell (diffuse) lymph
cancer	B627600	Diffuse non-Hodgkin's immunoblastic (diffuse) lymphoma
cancer	B627700	Diffuse non-Hodgkin's lymphoblastic (diffuse) lymphoma
cancer	B627800	Diffuse non-Hodgkin's lymphoma undiff (diffuse)
cancer	B627900	Mucosa-associated lymphoma
cancer	B627911	Maltoma
cancer	B627A00	Diffuse non-Hodgkin's large cell lymphoma
cancer	B627B00	Other types of follicular non-Hodgkin's lymphoma
cancer	B627C00	Follicular non-Hodgkin's lymphoma
cancer	B627C11	Follicular lymphoma NOS
cancer	B627D00	Diffuse non-Hodgkin's centroblastic lymphoma
cancer	B627E00	Diffuse large B-cell lymphoma
cancer	B627F00	Extranod marg zone Bcell lymph mucosa-assoc lymph tiss
cancer	B627G00	Mediastinal (thymic) large B-cell lymphoma
cancer	B627W00	Unspecified B-cell non-Hodgkin's lymphoma
cancer	B627X00	Diffuse non-Hodgkin's lymphoma, unspecified
cancer	B628.00	Follicular lymphoma
cancer	B628000	Follicular lymphoma grade 1
cancer	B628100	Follicular lymphoma grade 2
cancer	B628200	Follicular lymphoma grade 3

cancer	B628300	Follicular lymphoma grade 3a
cancer	B628400	Follicular lymphoma grade 3b
cancer	B628500	Diffuse follicle centre lymphoma
cancer	B628600	Cutaneous follicle centre lymphoma
cancer	B628700	Other types of follicular lymphoma
cancer	B629.00	Multifocal multisyste dissem Langerhans-cell histiocytosi
cancer	B62A.00	Sarcoma of dendritic cells
cancer	B62C.00	Unifocal Langerhans-cell histiocytosis
cancer	B62D.00	Histiocytic sarcoma
cancer	B62E.00	T/NK-cell lymphoma
cancer	B62E100	Anaplastic large cell lymphoma, ALK-positive
cancer	B62E200	Anaplastic large cell lymphoma, ALK-negative
cancer	B62E300	Cutaneous T-cell lymphoma
cancer	B62E500	Hepatosplenic T-cell lymphoma
cancer	B62E600	Enteropathy-associated T-cell lymphoma
cancer	B62E700	Subcutaneous panniculitic T-cell lymphoma
cancer	B62E800	Blastic NK-cell lymphoma
cancer	B62E900	Angioimmunoblastic T-cell lymphoma
cancer	B62EA00	Primary cutaneous CD30-positive T-cell proliferations
cancer	B62Ew00	Other mature T/NK-cell lymphoma
cancer	B62F.00	Nonfollicular lymphoma
cancer	B62F.11	Non-follicular lymphoma
cancer	B62F000	Small cell B-cell lymphoma
cancer	B62F100	Mantle cell lymphoma
cancer	B62F200	Lymphoblastic (diffuse) lymphoma
cancer	B62x.00	Malignant lymphoma otherwise specified
cancer	B62x000	T-zone lymphoma
cancer	B62x100	Lymphoepithelioid lymphoma
cancer	B62x200	Peripheral T-cell lymphoma
cancer	B62x400	Malignant reticulosis
cancer	B62x500	Malignant immunoproliferative small intestinal dis
cancer	B62x600	True histiocytic lymphoma
cancer	B62xX00	Oth and unspecif peripheral cutaneous T-cell lymphomas
cancer	B62y.00	Malignant lymphoma NOS
cancer	B62y000	Malignant lymphoma NOS of unspecified site
cancer	B62y100	Malig lymphoma NOS of lymph nodes of head, face, neck
cancer	B62y200	Malignant lymphoma NOS of intrathoracic lymph nodes
cancer	B62y300	Malig lymphoma NOS of intra-abdominal lymph nodes
cancer	B62y400	Malignant lymphoma NOS of lymph nodes of axilla arm
cancer	B62y500	Malig lymphoma NOS of lymph node inguinal region leg
cancer	B62y600	Malignant lymphoma NOS of intrapelvic lymph nodes
cancer	B62y700	Malignant lymphoma NOS of spleen
cancer	B62y800	Malignant lymphoma NOS of lymph nodes multiple sites
cancer	B62yz00	Malignant lymphoma NOS

cancer	B62z.00	Malignant neoplasms of lymphoid and histiocytic tissue
cancer	B62z000	Unspec malig neop lymphoid/histiocytic of unspec site
cancer	B62z100	Unspec malig neop lymphoid/hist lymph node head/neck
cancer	B62z200	Unspec malig neop lymphoid/histioc of intrathoracic node
cancer	B62z300	Unspec malig neop lymphoid/histioc intraabdom nodes
cancer	B62z400	Unspec malig neop lymph/histioclymph node axilla/arm
cancer	B62z500	Unspec malig neop lymph/histiocytic nodes inguinal/leg
cancer	B62z800	Unspec malig neop lymphoid/histiocytic of multiple sites
cancer	B62zz00	Lymphoid and histiocytic malignancy NOS
cancer	B62zz11	Immunoproliferative neoplasm
cancer	B63..00	Multiple myeloma and immunoproliferative neoplasms
cancer	B630.00	Multiple myeloma
cancer	B630.11	Kahler's dis
cancer	B630.12	Myelomatosis
cancer	B630000	Malig plasma cell neoplasm, extramedullary plasmacyto
cancer	B630100	Solitary myeloma
cancer	B630200	Plasmacytoma NOS
cancer	B630300	Lambda light chain myeloma
cancer	B630400	Solitary plasmacytoma
cancer	B631.00	Plasma cell leukaemia
cancer	B63y.00	Other immunoproliferative neoplasms
cancer	B63z.00	Immunoproliferative neoplasm or myeloma NOS
cancer	B64..00	Lymphoid leukaemia
cancer	B64..11	Lymphatic leukaemia
cancer	B640.00	Acute lymphoid leukaemia
cancer	B640000	B-cell acute lymphoblastic leukaemia
cancer	B641.00	Chronic lymphoid leukaemia
cancer	B641.11	Chronic lymphatic leukaemia
cancer	B641000	B-cell chronic lymphocytic leukaemia
cancer	B641011	Chronic lymphocytic leukaemia of B-cell type
cancer	B641100	Clinical stage A chronic lymphocytic leukaemia
cancer	B641200	Clinical stage B chronic lymphocytic leukaemia
cancer	B641300	Clinical stage C chronic lymphocytic leukaemia
cancer	B642.00	Subacute lymphoid leukaemia
cancer	B64y.00	Other lymphoid leukaemia
cancer	B64y100	Prolymphocytic leukaemia
cancer	B64y200	Adult T-cell leukaemia
cancer	B64y300	B-cell prolymphocytic leukaemia
cancer	B64y400	T-cell prolymphocytic leukaemia
cancer	B64y500	Adult T-cell lymphoma/leukaemia (HTLV-1-associated)
cancer	B64yz00	Other lymphoid leukaemia NOS
cancer	B64z.00	Lymphoid leukaemia NOS
cancer	B65..00	Myeloid leukaemia
cancer	B650.00	Acute myeloid leukaemia

cancer	B651.00	Chronic myeloid leukaemia
cancer	B651.11	Chronic granulocytic leukaemia
cancer	B651000	Chronic eosinophilic leukaemia
cancer	B651100	Chronic myeloid leukaemia, BCR/ABL positive
cancer	B651200	Chronic neutrophilic leukaemia
cancer	B651300	Atypical chronic myeloid leukaemia, BCR/ABL negative
cancer	B651z00	Chronic myeloid leukaemia NOS
cancer	B652.00	Subacute myeloid leukaemia
cancer	B653.00	Myeloid sarcoma
cancer	B653000	Chloroma
cancer	B653100	Granulocytic sarcoma
cancer	B654.00	Acute myeloblastic leukaemia
cancer	B65y100	Acute promyelocytic leukaemia
cancer	B65yz00	Other myeloid leukaemia NOS
cancer	B65z.00	Myeloid leukaemia NOS
cancer	B66..00	Monocytic leukaemia
cancer	B66..11	Histiocytic leukaemia
cancer	B66..12	Monoblastic leukaemia
cancer	B660.00	Acute monocytic leukaemia
cancer	B661.00	Chronic monocytic leukaemia
cancer	B662.00	Subacute monocytic leukaemia
cancer	B663.00	Acute monoblastic leukaemia
cancer	B66y.00	Other monocytic leukaemia
cancer	B66yz00	Other monocytic leukaemia NOS
cancer	B66z.00	Monocytic leukaemia NOS
cancer	B67..00	Other specified leukaemia
cancer	B670.00	Acute erythraemia and erythroleukaemia
cancer	B670.11	Di Guglielmo's dis
cancer	B671.00	Chronic erythraemia
cancer	B671.11	Heilmeyer - Schoner dis
cancer	B672.00	Megakaryocytic leukaemia
cancer	B672.11	Thrombocytic leukaemia
cancer	B673.00	Mast cell leukaemia
cancer	B674.00	Acute panmyelosis
cancer	B675.00	Acute myelofibrosis
cancer	B677.00	Myelodysplastic and myeloproliferative dis
cancer	B67y.00	Other and unspecified leukaemia
cancer	B67y000	Lymphosarcoma cell leukaemia
cancer	B67yz00	Other and unspecified leukaemia NOS
cancer	B67z.00	Other specified leukaemia NOS
cancer	B68..00	Leukaemia of unspecified cell type
cancer	B680.00	Acute leukaemia NOS
cancer	B681.00	Chronic leukaemia NOS
cancer	B682.00	Subacute leukaemia NOS

cancer	B68y.00	Other leukaemia of unspecified cell type
cancer	B68z.00	Leukaemia NOS
cancer	B69..00	Myelomonocytic leukaemia
cancer	B690.00	Acute myelomonocytic leukaemia
cancer	B691.00	Chronic myelomonocytic leukaemia
cancer	B692.00	Subacute myelomonocytic leukaemia
cancer	B693.00	Juvenile myelomonocytic leukaemia
cancer	B6y..00	Malignant neoplasm lymphatic or haematopoietic tissue
cancer	B6y0.00	Myeloproliferative disorder
cancer	B6y0.11	Myeloproliferative dis
cancer	B6y1.00	Myelosclerosis with myeloid metaplasia
cancer	B6z..00	Malignant neoplasm lymphatic or haematopoietic tissue
cancer	B6z0.00	Kaposi's sarcoma of lymph nodes
cancer	B8...00	Carcinoma in situ
cancer	B80..00	Carcinoma in situ of digestive organs
cancer	B80..11	Ca-in-situ of G.I. tract
cancer	B800.00	Carcinoma in situ of lip, oral cavity and pharynx
cancer	B800.11	Carcinoma in situ of oral cavity
cancer	B800.12	Carcinoma in situ of pharynx
cancer	B800000	Carcinoma in situ of lip
cancer	B800100	Carcinoma in situ of tongue
cancer	B800200	Carcinoma in situ of salivary glands
cancer	B800300	Carcinoma in situ of gums
cancer	B800400	Carcinoma in situ of floor of mouth
cancer	B800500	Carcinoma in situ of cheek
cancer	B800600	Carcinoma in situ of palate
cancer	B800700	Carcinoma in situ of nasopharynx
cancer	B800800	Carcinoma in situ of oropharynx
cancer	B800900	Carcinoma in situ of hypopharynx
cancer	B800z00	Carcinoma in situ of lip, oral cavity and pharynx NOS
cancer	B801.00	Carcinoma in situ of oesophagus
cancer	B801000	Carcinoma in situ of upper 1/3 oesophagus
cancer	B801100	Carcinoma in situ of middle 1/3 oesophagus
cancer	B801200	Carcinoma in situ of lower 1/3 oesophagus
cancer	B801z00	Carcinoma in situ of oesophagus NOS
cancer	B802.00	Carcinoma in situ of stomach
cancer	B802000	Carcinoma in situ of cardia of stomach
cancer	B802100	Carcinoma in situ of fundus of stomach
cancer	B802200	Carcinoma in situ of body of stomach
cancer	B802300	Carcinoma in situ of pyloric antrum
cancer	B802400	Carcinoma in situ of pyloric canal
cancer	B802z00	Carcinoma in situ of stomach NOS
cancer	B803.00	Carcinoma in situ of colon
cancer	B803000	Carcinoma in situ of hepatic flexure of colon

cancer	B803100	Carcinoma in situ of transverse colon
cancer	B803200	Carcinoma in situ of descending colon
cancer	B803300	Carcinoma in situ of sigmoid colon
cancer	B803400	Carcinoma in situ of caecum
cancer	B803500	Carcinoma in situ of appendix
cancer	B803600	Carcinoma in situ of ascending colon
cancer	B803700	Carcinoma in situ of splenic flexure of colon
cancer	B803800	High grade dysplasia of colon
cancer	B803z00	Carcinoma in situ of colon NOS
cancer	B804.00	Carcinoma in situ of rectum and rectosigmoid junction
cancer	B804000	Carcinoma in situ of rectosigmoid junction
cancer	B804100	Carcinoma in situ of rectum
cancer	B804z00	Carcinoma in situ of rectum or rectosigmoid junction NOS
cancer	B805.00	Carcinoma in situ of anal canal
cancer	B805000	Anal intraepithelial neoplasia grade III
cancer	B806.00	Carcinoma in situ of anus NOS
cancer	B807.00	Carcinoma in situ of other and unspecified small intestine
cancer	B807000	Carcinoma in situ of duodenum
cancer	B807100	Carcinoma in situ of jejunum
cancer	B807200	Carcinoma in situ of ileum
cancer	B807300	Carcinoma in situ of Meckel's diverticulum
cancer	B807z00	Carcinoma in situ other and unspecified small intestine
cancer	B808.00	Carcinoma in situ of liver and biliary system
cancer	B808.11	Carcinoma in situ of biliary system
cancer	B808000	Carcinoma in situ of liver
cancer	B808100	Carcinoma in situ of intrahepatic bile ducts
cancer	B808200	Carcinoma in situ of hepatic duct
cancer	B808300	Carcinoma in situ of gall bladder
cancer	B808400	Carcinoma in situ of cystic duct
cancer	B808500	Carcinoma in situ of common bile duct
cancer	B808600	Carcinoma in situ of ampulla of Vater
cancer	B808z00	Carcinoma in situ of liver or biliary system NOS
cancer	B80z.00	Carcinoma in situ of other and unspecified digest organs
cancer	B80z000	Carcinoma in situ of pancreas
cancer	B80z100	Carcinoma in situ of spleen
cancer	B81..00	Carcinoma in situ of respiratory system
cancer	B810.00	Carcinoma in situ of larynx
cancer	B810000	Carcinoma in situ of thyroid cartilage
cancer	B810100	Carcinoma in situ of cricoid cartilage
cancer	B810200	Carcinoma in situ of epiglottis
cancer	B810300	Carcinoma in situ of arytenoid cartilage
cancer	B810600	Carcinoma in situ of aryepiglottic fold
cancer	B810700	Carcinoma in situ of vestibular fold
cancer	B810800	Carcinoma in situ of vocal fold - glottis

cancer	B810811	Carcinoma in situ of glottis
cancer	B810z00	Carcinoma in situ of larynx NOS
cancer	B811.00	Carcinoma in situ of trachea
cancer	B812.00	Carcinoma in situ of bronchus and lung
cancer	B812000	Carcinoma in situ of carina of bronchus
cancer	B812100	Carcinoma in situ of main bronchus
cancer	B812200	Carcinoma in situ of upper lobe bronchus and lung
cancer	B812300	Carcinoma in situ of middle lobe bronchus and lung
cancer	B812400	Carcinoma in situ of lower lobe bronchus and lung
cancer	B812z00	Carcinoma in situ of bronchus or lung NOS
cancer	B81y.00	Carcinoma in situ other specified part respiratory system
cancer	B81y.11	Carcinoma in situ of nasal sinuses
cancer	B81y000	Carcinoma in situ of pleura
cancer	B81y100	Carcinoma in situ of nasal cavity
cancer	B81y400	Carcinoma in situ of Eustachian tube
cancer	B81y500	Carcinoma in situ of mastoid air cells
cancer	B81y600	Carcinoma in situ of maxillary sinus
cancer	B81y700	Carcinoma in situ of ethmoidal sinus
cancer	B81y900	Carcinoma in situ of sphenoidal sinus
cancer	B81yz00	Carcinoma in situ of specified parts respiratory system
cancer	B81z.00	Carcinoma in situ of respiratory organ NOS
cancer	B828.00	Melanoma in situ of skin
cancer	B828000	Melanoma in situ of lip
cancer	B828100	Melanoma in situ of eyelid, including canthus
cancer	B828200	Melanoma in situ of ear and external auricular canal
cancer	B828300	Melanoma in situ of scalp and neck
cancer	B828400	Melanoma in situ of trunk
cancer	B828500	Melanoma in situ of upper limb, including shoulder
cancer	B828600	Melanoma in situ of lower limb, including hip
cancer	B828700	Melanoma in situ of scalp
cancer	B828800	Melanoma in situ of back of hand
cancer	B828900	Melanoma in situ of back
cancer	B828W00	Melanoma in situ, unspecified
cancer	B828X00	Melanoma in situ of other and unspecified parts of face
cancer	B83..00	Carcinoma in situ of breast and genitourinary system
cancer	B830.00	Carcinoma in situ of breast
cancer	B830000	Lobular carcinoma in situ of breast
cancer	B830100	Intraductal carcinoma in situ of breast
cancer	B831.00	Carcinoma in situ of cervix uteri
cancer	B831.11	CIN III - carcinoma in situ of cervix
cancer	B831.12	Cervical intraepithelial neoplasia
cancer	B831.13	Cervical intraepithelial neoplasia grade III
cancer	B831000	Carcinoma in situ of endocervix
cancer	B831100	Carcinoma in situ of exocervix

cancer	B832.00	Carcinoma in situ of other and unspecified parts of uterus
cancer	B832.11	Carcinoma in situ of body of uterus
cancer	B832000	Carcinoma in situ of endometrium
cancer	B833.00	Carcinoma in situ other unspecified female genital organ
cancer	B833000	Carcinoma in situ of ovary
cancer	B833100	Carcinoma in situ of fallopian tube
cancer	B833200	Carcinoma in situ of vagina
cancer	B833300	Carcinoma in situ of vulva
cancer	B833311	Vulval intraepithelial neoplasia
cancer	B833400	Vulval intraepithelial neoplasia grade 1
cancer	B833500	Vulval intraepithelial neoplasia grade 2
cancer	B833600	Vulval intraepithelial neoplasia grade 3
cancer	B833700	Vaginal intraepithelial neoplasia grade 1
cancer	B833800	Vaginal intraepithelial neoplasia grade 2
cancer	B833900	Vaginal intraepithelial neoplasia grade 3
cancer	B833z00	Carcinoma in situ of female genital organs NOS
cancer	B834.00	Carcinoma in situ of prostate
cancer	B834000	High grade prostatic intraepithelial neoplasia
cancer	B834100	Prostatic intraepithelial neoplasia
cancer	B835.00	Carcinoma in situ of penis
cancer	B836.00	Carcinoma in situ other unspecified male genital organs
cancer	B836000	Carcinoma in situ of testis
cancer	B836300	Carcinoma in situ of scrotum
cancer	B837.00	Carcinoma in situ of bladder
cancer	B83z.00	Carcinoma in situ of urinary organs NOS
cancer	B8y..00	Carcinoma in situ of other and unspecified sites
cancer	B8y0.00	Carcinoma in situ of eye
cancer	B8yy.00	Carcinoma in situ of other specified site
cancer	B8yy000	Carcinoma in situ of thyroid gland
cancer	B8yy100	Carcinoma in situ of adrenal gland
cancer	B8yy200	Carcinoma in situ of parathyroid gland
cancer	B8yy300	Carcinoma in situ of pituitary gland
cancer	B8yyz00	Carcinoma in situ of other specified site NOS
cancer	B8z..00	Carcinoma in situ NOS
cancer	B911000	Malignant hydatidiform mole
cancer	B911012	Invasive mole - placenta
cancer	B911013	Choriocarcinoma
cancer	B934.00	Polycythaemia vera
cancer	B934.11	Polycythaemia rubra vera
cancer	B934.12	Primary polycythaemia
cancer	B937.00	Neop uncertain behaviour lymphatic/haematopoietic tiss
cancer	B937.11	Neoplasm of uncertain behaviour of blood
cancer	B937.12	Idiopathic thrombocythaemia
cancer	B937.14	Myelodysplasia

cancer	B937000	Refractory anaemia without sideroblasts, so stated
cancer	B937100	Refractory anaemia with sideroblasts
cancer	B937200	Refractory anaemia with excess of blasts
cancer	B937300	Refractory anaemia excess of blasts with transformation
cancer	B937400	Essential (haemorrhagic) thrombocythaemia
cancer	B937411	Primary thrombocythaemia
cancer	B937500	Idiopathic thrombocythaemia
cancer	B937600	Refractory anaemia without ring sideroblasts
cancer	B937700	Refractory anaemia with ring sideroblasts
cancer	B937800	Refractory anaemia with multilineage dysplasia
cancer	B937900	5Q minus synde
cancer	B937911	Myelodysplastic synd isolated del(5q) chromos abnorm
cancer	B937W00	Myelodysplastic synde, unspecified
cancer	B937W11	Myelodysplasia
cancer	B937X00	Refractory anaemia, unspecified
cancer	BB02.00	[M]Neoplasm, malignant
cancer	BB03.12	[M]Tumour embolus
cancer	BB03.13	[M]Tumour embolism
cancer	BB04.00	[M]Neopl, malig, uncertain whether primary/ metastatic
cancer	BB07.00	[M]Tumour cells, malignant
cancer	BB08.00	[M]Malignant tumour, small cell type
cancer	BB09.00	[M]Malignant tumour, giant cell type
cancer	BB0A.00	[M]Malignant tumour, fusiform cell type
cancer	BB11.00	[M]Carcinoma in situ NOS
cancer	BB11.11	[M]Intraepithelial carcinoma NOS
cancer	BB12.00	[M]Carcinoma NOS
cancer	BB14.00	[M]Carcinomatosis
cancer	BB16.00	[M]Epithelioma, malignant
cancer	BB17.00	[M]Large cell carcinoma NOS
cancer	BB18.00	[M]Carcinoma, undifferentiated type, NOS
cancer	BB19.00	[M]Carcinoma, anaplastic type, NOS
cancer	BB1A.00	[M]Pleomorphic carcinoma
cancer	BB1B.00	[M]Giant cell and spindle cell carcinoma
cancer	BB1C.00	[M]Giant cell carcinoma
cancer	BB1D.00	[M]Spindle cell carcinoma
cancer	BB1E.00	[M]Pseudosarcomatous carcinoma
cancer	BB1F.00	[M]Polygonal cell carcinoma
cancer	BB1G.00	[M]Spheroidal cell carcinoma
cancer	BB1J.00	[M]Small cell carcinoma NOS
cancer	BB1J.12	[M]Round cell carcinoma
cancer	BB1K.00	[M]Oat cell carcinoma
cancer	BB1L.00	[M]Small cell carcinoma, fusiform cell type
cancer	BB1M.00	[M]Small cell carcinoma, intermediate cell
cancer	BB1N.00	[M]Small cell-large cell carcinoma

cancer	BB1P.00	[M]Non-small cell carcinoma
cancer	BB21.00	[M]Papillary carcinoma in situ
cancer	BB22.00	[M]Papillary carcinoma NOS
cancer	BB24.00	[M]Verrucous carcinoma NOS
cancer	BB24.11	[M]Verrucous epidermoid carcinoma
cancer	BB26.00	[M]Papillary squamous cell carcinoma
cancer	BB26.11	[M]Papillary epidermoid carcinoma
cancer	BB29.00	[M]Squamous cell carcinoma in situ NOS
cancer	BB29.13	[M]Intraepithelial squamous cell carcinoma
cancer	BB2A.00	[M]Squamous cell carcinoma NOS
cancer	BB2B.00	[M]Squamous cell carcinoma, metastatic NOS
cancer	BB2D.00	[M]Squamous cell carcinoma, large cell, non-keratinising
cancer	BB2E.00	[M]Squamous cell carcinoma, small cell, non-keratinising
cancer	BB2G.00	[M]Adenoid squamous cell carcinoma
cancer	BB2H.00	[M]Squamous cell cain-situ, questionable stromal invasion
cancer	BB2J.00	[M]Squamous cell carcinoma, microinvasive
cancer	BB2M.00	[M]Lymphoepithelial carcinoma
cancer	BB2N.00	[M]Intraepit neop,grade III,of cervix, vulva and vagina
cancer	BB42.00	[M]Transitional cell carcinoma in situ
cancer	BB43.00	[M]Transitional cell carcinoma NOS
cancer	BB43.11	[M]Urothelial carcinoma
cancer	BB46.00	[M]Schneiderian carcinoma
cancer	BB47.00	[M]Transitional cell carcinoma, spindle cell type
cancer	BB48.00	[M]Basaloid carcinoma
cancer	BB49.00	[M]Cloacogenic carcinoma
cancer	BB4A.00	[M]Papillary transitional cell carcinoma
cancer	BB4B.00	[M]Grade 1 (Stage pTa) papillary urothelial/transit cell ca
cancer	BB4C.00	[M]Grade 2 (Stage pTa) papillary urothelial/transit cell ca
cancer	BB4D.00	[M]Grade 3 (Stage pTa) papillary urothelial/transit cell ca
cancer	BB4z.00	[M]Transitional cell papilloma or carcinoma NOS
cancer	BB5..00	[M]Adenomas and adenocarcinomas
cancer	BB5..11	[M]Adenocarcinomas
cancer	BB51.00	[M]Adenocarcinoma in situ
cancer	BB51000	[M]Adenocarcinoma in situ in villous adenoma
cancer	BB51100	[M]Adenocarcinoma in situ in tubulovillous adenoma
cancer	BB52.00	[M]Adenocarcinoma NOS
cancer	BB52000	[M]Adenocarcinoma in tubulovillous adenoma
cancer	BB53.00	[M]Adenocarcinoma, metastatic, NOS
cancer	BB54.00	[M]Scirrhous adenocarcinoma
cancer	BB55.00	[M]Linitis plastica
cancer	BB56.00	[M]Superficial spreading adenocarcinoma
cancer	BB57.00	[M]Adenocarcinoma, intestinal type
cancer	BB58.00	[M]Carcinoma, diffuse type
cancer	BB5a.00	[M]Renal adenoma and carcinoma

cancer	BB5a000	[M]Renal cell carcinoma
cancer	BB5a011	[M]Grawitz tumour
cancer	BB5a012	[M]Hypernephroma
cancer	BB5b.00	[M]Granular cell carcinoma
cancer	BB5B.00	[M]Pancreatic adenomas and carcinomas
cancer	BB5B011	[M]Nesidioblastoma
cancer	BB5B100	[M]Islet cell carcinoma
cancer	BB5B300	[M]Insulinoma, malignant
cancer	BB5B500	[M]Glucagonoma, malignant
cancer	BB5B600	[M]Mixed islet cell and exocrine adenocarcinoma
cancer	BB5c.00	[M]Parathyroid adenomas and adenocarcinomas
cancer	BB5C.00	[M]Gastrinoma and carcinomas
cancer	BB5C000	[M]Gastrinoma NOS
cancer	BB5C100	[M]Gastrinoma, malignant
cancer	BB5D.00	[M]Hepatobiliary tract adenomas and carcinomas
cancer	BB5D.11	[M]Biliary tract adenomas and adenocarcinomas
cancer	BB5D100	[M]Cholangiocarcinoma
cancer	BB5D111	[M]Bile duct carcinoma
cancer	BB5D300	[M]Bile duct cystadenocarcinoma
cancer	BB5D500	[M]Hepatocellular carcinoma NOS
cancer	BB5D512	[M]Hepatoma, malignant
cancer	BB5D513	[M]Liver cell carcinoma
cancer	BB5D700	[M]Combined hepatocell carcinoma/ cholangiocarcinoma
cancer	BB5D800	[M]Hepatocellular carcinoma, fibrolamellar
cancer	BB5F.00	[M]Trabecular adenocarcinoma
cancer	BB5f.00	[M]Thyroid adenoma and adenocarcinoma
cancer	BB5f100	[M]Follicular adenocarcinoma NOS
cancer	BB5f111	[M]Follicular carcinoma
cancer	BB5f200	[M]Follicular adenocarcinoma, well differentiated type
cancer	BB5f300	[M]Follicular adenocarcinoma, trabecular type
cancer	BB5f600	[M]Papillary and follicular adenocarcinoma
cancer	BB5f700	[M]Nonencapsulated sclerosing carcinoma
cancer	BB5h100	[M]Adrenal cortical carcinoma
cancer	BB5J.00	[M]Adenoid cystic carcinoma
cancer	BB5j.00	[M]Endometrioid adenomas and carcinomas
cancer	BB5J.11	[M]Cylindroid adenocarcinoma
cancer	BB5j100	[M]Endometrioid adenoma, borderline malignancy
cancer	BB5j200	[M]Endometrioid carcinoma
cancer	BB5j400	[M]Endometrioid adenofibroma, borderline malignancy
cancer	BB5j500	[M]Endometrioid adenofibroma, malignant
cancer	BB5K.00	[M]Cribriform carcinoma
cancer	BB5L.00	[M]Adenomatous and adenocarcinomatous polyps
cancer	BB5L100	[M]Adenocarcinoma in adenomatous polyp
cancer	BB5L200	[M]Adenocarcinoma in situ in adenomatous polyp

cancer	BB5L300	[M]Adenocarcinoma in multiple adenomatous polyps
cancer	BB5M.00	[M]Tubular adenomas and adenocarcinomas
cancer	BB5M100	[M]Tubular adenocarcinoma
cancer	BB5N.00	[M]Adenomatous adenocarcinomatous polyps of colon
cancer	BB5N100	[M]Adenocarcinoma in adenomatous polposis coli
cancer	BB5P.00	[M]Solid carcinoma NOS
cancer	BB5R100	[M]Carcinoid tumour, malignant
cancer	BB5R500	[M]Carcinoid tumour, nonargentaffin, malignant
cancer	BB5R600	[M]Mucocarcinoid tumour, malignant
cancer	BB5R611	[M]Goblet cell tumour
cancer	BB5R800	[M]Adenocarcinoid tumour
cancer	BB5R900	[M]Neuroendocrine carcinoma
cancer	BB5S.00	[M]Respiratory tract adenomas and adenocarcinomas
cancer	BB5S200	[M]Bronchiolo-alveolar adenocarcinoma
cancer	BB5S211	[M]Alveolar cell carcinoma
cancer	BB5S212	[M]Bronchiolar carcinoma
cancer	BB5S400	[M]Alveolar adenocarcinoma
cancer	BB5T.00	[M]Papillary adenomas and adenocarcinomas
cancer	BB5T100	[M]Papillary adenocarcinoma NOS
cancer	BB5U.00	[M]Villous adenomas and adenocarcinomas
cancer	BB5U100	[M]Adenocarcinoma in villous adenoma
cancer	BB5U200	[M]Villous adenocarcinoma
cancer	BB5V.00	[M]Pituitary adenomas and carcinomas
cancer	BB5V100	[M]Chromophobe carcinoma
cancer	BB5V311	[M]Eosinophil carcinoma
cancer	BB5V700	[M]Basophil carcinoma
cancer	BB5V711	[M]Mucoid cell carcinoma
cancer	BB5W.00	[M]Oxyphilic adenomas and adenocarcinomas
cancer	BB5W100	[M]Oxyphilic adenocarcinoma
cancer	BB5W111	[M]Hurthle cell adenocarcinoma
cancer	BB5W112	[M]Oncytic adenocarcinoma
cancer	BB5X.00	[M]Clear cell adenomas and adenocarcinomas
cancer	BB5X100	[M]Clear cell adenocarcinoma NOS
cancer	BB5Y.00	[M]Hypernephroid tumour
cancer	BB5y.00	[M]Adenoma and adenocarcinoms OS
cancer	BB5y100	[M]Vipoma
cancer	BB5y200	[M]Klatskin's tumour
cancer	BB71.00	[M]Mucoepidermoid carcinoma
cancer	BB80.00	[M]Cystadenoma and carcinoma
cancer	BB80100	[M]Cystadenocarcinoma NOS
cancer	BB80200	[M]Borderline mucinous cystadenoma of the ovary
cancer	BB81100	[M]Serous cystadenoma, borderline malignancy
cancer	BB81200	[M]Serous cystadenocarcinoma, NOS
cancer	BB81400	[M]Papillary cystadenoma, borderline malignancy

cancer	BB81500	[M]Papillary cystadenocarcinoma, NOS
cancer	BB81800	[M]Papillary serous cystadenocarcinoma
cancer	BB81A00	[M]Serous surface papilloma, borderline malignancy
cancer	BB81B00	[M]Serous surface papillary carcinoma
cancer	BB81D00	[M]Mucinous cystadenoma, borderline malignancy
cancer	BB81E00	[M]Mucinous cystadenocarcinoma NOS
cancer	BB81E11	[M]Pseudomucinous adenocarcinoma
cancer	BB81H00	[M]Papillary mucinous cystadenocarcinoma
cancer	BB81J00	[M]Serous cystadenoma, borderline malignancy
cancer	BB81K00	[M]Papillary cystadenoma, borderline malignancy
cancer	BB81M00	[M]Papillary serous cystadenoma, borderline malignancy
cancer	BB82.00	[M]Mucinous adenoma and adenocarcinoma
cancer	BB82100	[M]Mucinous adenocarcinoma
cancer	BB82111	[M]Colloid adenocarcinoma
cancer	BB82112	[M]Gelatinous adenocarcinoma
cancer	BB82113	[M]Mucoid adenocarcionoma
cancer	BB82114	[M]Mucous adenocarcinoma
cancer	BB84.00	[M]Mucin-producing adenocarcinoma
cancer	BB85.00	[M]Signet ring carcinoma
cancer	BB85000	[M]Signet ring cell carcinoma
cancer	BB85100	[M]Metastatic signet ring cell carcinoma
cancer	BB85111	[M]Krukenberg tumour
cancer	BB85z00	[M]Signet ring carcinoma NOS
cancer	BB90.00	[M]Intraductal carcinoma, noninfiltrating NOS
cancer	BB91.00	[M]Infiltrating duct carcinoma
cancer	BB91.11	[M]Duct carcinoma NOS
cancer	BB91000	[M]Intraductal papillary adenocarcinoma with invasion
cancer	BB91100	[M]Infiltrating duct and lobular carcinoma
cancer	BB92.00	[M]Comedocarcinoma, noninfiltrating
cancer	BB93.00	[M]Comedocarcinoma NOS
cancer	BB94.00	[M]Juvenile breast carcinoma
cancer	BB94.11	[M]Secretory breast carcinoma
cancer	BB96.00	[M]Noninfiltrating intraductal papillary adenocarcinoma
cancer	BB9B.00	[M]Medullary carcinoma NOS
cancer	BB9B.11	[M]C cell carcinoma
cancer	BB9C.00	[M]Medullary carcinoma with amyloid stroma
cancer	BB9D.00	[M]Medullary carcinoma with lymphoid stroma
cancer	BB9E.00	[M]Lobular carcinoma in situ
cancer	BB9E000	[M]Intraductal carcinoma and lobular carcinoma in situ
cancer	BB9F.00	[M]Lobular carcinoma NOS
cancer	BB9G.00	[M]Infiltrating ductular carcinoma
cancer	BB9H.00	[M]Inflammatory carcinoma
cancer	BB9J.00	[M]Paget's dis, mammary
cancer	BB9J.11	[M]Paget's dis, breast

cancer	BB9K.00	[M]Paget's dis and infiltrating breast duct carcinoma
cancer	BB9K000	[M]Paget's dis and intraductal carcinoma of breast
cancer	BB9L.00	[M]Paget's dis, extramammary, exc Paget's dis bone
cancer	BB9M.00	[M]Intracystic carcinoma NOS
cancer	BBa0.00	[M]Craniopharyngioma
cancer	BBa0.11	[M]Rathke's pouch tumour
cancer	BBA2.00	[M]Acinar cell carcinoma
cancer	BBa3.00	[M]Pineoblastoma
cancer	BBb..00	[M]Gliomas
cancer	BBb0.00	[M]Glioma, malignant
cancer	BBB0.00	[M]Adenosquamous carcinoma
cancer	BBb0.11	[M]Glioma NOS
cancer	BBb0.12	[M]Gliosarcoma
cancer	BBb1.00	[M]Gliomatosis cerebri
cancer	BBb2.00	[M]Mixed glioma
cancer	BBB2.00	[M]Adenocarcinoma with squamous metaplasia
cancer	BBb2.11	[M]Mixed glioma
cancer	BBb3.00	[M]Subependymal glioma
cancer	BBB3.00	[M]Adenocarcinoma with cartilaginous osseous metaplasi
cancer	BBb3.11	[M]Subependymal astrocytoma NOS
cancer	BBb3.12	[M]Subependymal astrocytoma NOS
cancer	BBb3.13	[M]Subependymoma
cancer	BBb4.00	[M]Subependymal giant cell astrocytoma
cancer	BBB4.00	[M]Adenocarcinoma with spindle cell metaplasia
cancer	BBB5.00	[M]Adenocarcinoma with apocrine metaplasia
cancer	BBB6100	[M]Thymoma, malignant
cancer	BBb7.00	[M]Ependymoma NOS
cancer	BBB7.00	[M]Epithelial-myoepithelial carcinoma
cancer	BBb8.00	[M]Ependymoma, anaplastic type
cancer	BBb8.11	[M]Ependymoblastoma
cancer	BBb9.00	[M]Papillary ependymoma
cancer	BBbA.00	[M]Myxopapillary ependymoma
cancer	BBba000	[M]Peripheral neuroectodermal tumour
cancer	BBbB.00	[M]Astrocytoma NOS
cancer	BBbB.11	[M]Astrocytic glioma
cancer	BBbC.00	[M]Astrocytoma, anaplastic type
cancer	BBbE.00	[M]Gemistocytic astrocytoma
cancer	BBbF.00	[M]Fibrillary astrocytoma
cancer	BBbG.00	[M]Pilocytic astrocytoma
cancer	BBbG.11	[M]Juvenile astrocytoma
cancer	BBbG.12	[M]Piloid astrocytoma
cancer	BBbH.00	[M]Spongioblastoma NOS
cancer	BBbK.00	[M]Astroblastoma
cancer	BBbL.00	[M]Glioblastoma NOS

cancer	BBbL.11	[M]Glioblastoma multiforme
cancer	BBbM.00	[M]Giant cell glioblastoma
cancer	BBbQ.00	[M]Oligodendroglioma NOS
cancer	BBbR.00	[M]Oligodendroglioma, anaplastic type
cancer	BBbS.00	[M]Oligodendroblastoma
cancer	BBbT.00	[M]Medulloblastoma NOS
cancer	BBbU.00	[M]Desmoplastic medulloblastoma
cancer	BBbV.00	[M]Medullomyoblastoma
cancer	BBbW.00	[M]Cerebellar sarcoma NOS
cancer	BBbz.00	[M]Glioma NOS
cancer	BBbZ.00	[M]Pleomorphic xanthoastrocytoma
cancer	BBc3.00	[M]Teratoid medulloepithelioma
cancer	BBC4.00	[M]Granulosa cell tumour, malignant
cancer	BBc7.11	[M]Neuroastrocytoma
cancer	BBc9.00	[M]Retinoblastomas
cancer	BBc9100	[M]Retinoblastoma, undifferentiated type
cancer	BBc9z00	[M]Retinoblastoma NOS
cancer	BBCA.00	[M]Sertoli cell carcinoma
cancer	BBcC.00	[M]Aesthesioneuroblastoma
cancer	BBcC.11	[M]Olfactory neuroblastoma
cancer	BBCC100	[M]Leydig cell tumour, malignant
cancer	BBD1.00	[M]Paraganglioma, malignant
cancer	BBd2.00	[M]Meningioma, malignant
cancer	BBd2.11	[M]Leptomeningeal sarcoma
cancer	BBd2.12	[M]Meningothelial sarcoma
cancer	BBDA.00	[M]Phaeochromocytoma, malignant
cancer	BBdB.00	[M]Meningeal sarcomatosis
cancer	BBDB.00	[M]Glomangiosarcoma
cancer	BBDB.11	[M]Glomoid sarcoma
cancer	BBE..00	[M]Naevi and melanomas
cancer	BBE1.00	[M]Malignant melanoma NOS
cancer	BBE1.11	[M]Melanocarcinoma
cancer	BBE1.12	[M]Melanoma NOS
cancer	BBE1.13	[M]Melanosarcoma NOS
cancer	BBE1.14	[M]Naevocarcinoma
cancer	BBE1000	[M]Malignant melanoma, regressing
cancer	BBE1100	[M]Desmoplastic melanoma, malignant
cancer	BBE2.00	[M]Nodular melanoma
cancer	BBe2.00	[M]Neurofibrosarcoma
cancer	BBE4.00	[M]Balloon cell melanoma
cancer	BBe7.00	[M]Neurilemmoma, malignant
cancer	BBe7.11	[M]Schwannoma, malignant
cancer	BBE8.11	[M]Melanocytoma of eyeball
cancer	BBe9.00	[M]Triton tumour, malignant

cancer	BBEA.00	[M]Amelanotic melanoma
cancer	BBEC.00	[M]Malignant melanoma in junctional naevus
cancer	BBED.00	[M]Precancerous melanosis NOS
cancer	BBEF.00	[M]Hutchinson's melanotic freckle
cancer	BBEF.11	[M]Lentigo maligna
cancer	BBEG.00	[M]Malign melanoma in Hutchinson's melanotic freckle
cancer	BBEG.11	[M]Lentigo maligna melanoma
cancer	BBEG000	[M]Acral lentiginous melanoma, malignant
cancer	BBEH.00	[M]Superficial spreading melanoma
cancer	BBEM.00	[M]Malignant melanoma in giant pigmented naevus
cancer	BBEP.00	[M]Epithelioid cell melanoma
cancer	BBEQ.00	[M]Spindle cell melanoma NOS
cancer	BBES.00	[M]Spindle cell melanoma, type B
cancer	BBET.00	[M]Mixed epithelioid and spindle melanoma
cancer	BBEV.00	[M]Blue naevus, malignant
cancer	BBEX.00	[M]Melanoma in situ
cancer	BBf..00	[M]Granular cell tumours and alveolar soft part sarcoma
cancer	BBF..00	[M]Soft tissue tumours and sarcomas NOS
cancer	BBF1.00	[M]Sarcoma NOS
cancer	BBF2.00	[M]Sarcomatosis NOS
cancer	BBf2.00	[M]Alveolar soft part sarcoma
cancer	BBF3.00	[M]Spindle cell sarcoma
cancer	BBF4.00	[M]Giant cell sarcoma (except of bone)
cancer	BBF4.11	[M]Pleomorphic cell sarcoma
cancer	BBF5.00	[M]Small cell sarcoma
cancer	BBF5.11	[M]Round cell sarcoma
cancer	BBF6.00	[M]Epithelioid cell sarcoma
cancer	BBFz.00	[M]Soft tissue tumour or sarcoma NOS
cancer	BBg..00	[M]Lymphomas, NOS or diffuse
cancer	BBg1.00	[M]Malignant lymphoma NOS
cancer	BBG1.00	[M]Fibrosarcoma NOS
cancer	BBg1.11	[M]Lymphoma NOS
cancer	BBg1000	[M]Malignant lymphoma, diffuse NOS
cancer	BBg2.00	[M]Malignant lymphoma, non Hodgkin's type
cancer	BBg2.11	[M]Non Hodgkins lymphoma
cancer	BBg3.00	[M]Malignant lymphoma, undifferentiated cell type NOS
cancer	BBG3.00	[M]Fibromyxosarcoma
cancer	BBg4.00	[M]Malignant lymphoma, stem cell type
cancer	BBg5.00	[M]Malignant lymphoma, convoluted cell type NOS
cancer	BBg6.00	[M]Lymphosarcoma NOS
cancer	BBg7.00	[M]Malignant lymphoma, lymphoplasmacytoid type
cancer	BBg8.00	[M]Malignant lymphoma, immunoblastic type
cancer	BBG8.00	[M]Infantile fibrosarcoma
cancer	BBG8.11	[M]Congenital fibrosarcoma

cancer	BBgA.00	[M]Malignant lymphoma, centroblastic-centrocytic, diff
cancer	BBgB.00	[M]Malignant lymphoma, follicular centre cell NOS
cancer	BBgC.00	[M]Malignant lymphoma, lymphocytic, well differentiated
cancer	BBgC.11	[M]Lymphocytic lymphoma NOS
cancer	BBgC.12	[M]Lymphocytic lymphosarcoma NOS
cancer	BBgD.00	[M]Malig lymphoma, lymphocytic, intermediate different
cancer	BBgE.00	[M]Malignant lymphoma, centrocytic
cancer	BBGF.00	[M]Fibrous histiocytoma, malignant
cancer	BBgG.00	[M]Malignant lymphoma, lymphocytic, poorly different
cancer	BBgG.11	[M]Lymphoblastic lymphosarcoma NOS
cancer	BBgG.12	[M]Lymphoblastic lymphoma NOS
cancer	BBgG.13	[M]Lymphoblastoma NOS
cancer	BBgH.00	[M]Prolymphocytic lymphosarcoma
cancer	BBgJ.00	[M]Malignant lymphoma, centroblastic type NOS
cancer	BBGJ.00	[M]Fibroxanthoma, malignant
cancer	BBgJ.11	[M]Germinoblastic sarcoma NOS
cancer	BBGJ.11	[M]Fibroxanthosarcoma
cancer	BBgK.00	[M]Malig lymphoma, follicular centre cell, non-cleaved
cancer	BBgL.00	[M]Malignant lymphoma, small lymphocytic NOS
cancer	BBgM.00	[M]Malignant lymphoma, small cleaved cell, diffuse
cancer	BBGM.00	[M]Dermatofibrosarcoma NOS
cancer	BBgN.00	[M]Malign lymph,lymphocy,intermediate differn, diffuse
cancer	BBgP.00	[M]Malignant lymphoma, mixed small large cell, diffuse
cancer	BBGP.00	[M]Pigmented dermatofibrosarcoma protuberans
cancer	BBgQ.00	[M]Malignant lymphomatous polyposis
cancer	BBgR.00	[M]Malignant lymphoma, large cell, diffuse NOS
cancer	BBgS.00	[M]Malignant lymphoma, large cell, cleaved, diffuse
cancer	BBgT.00	[M]Malignant lymphoma, large cell, noncleaved, diffuse
cancer	BBgV.00	[M]Malignant lymphoma, small cell, noncleaved, diffuse
cancer	BBgz.00	[M]Lymphoma, diffuse or NOS
cancer	BBh..00	[M]Reticulosarcomas
cancer	BBh0.00	[M]Reticulosarcoma NOS
cancer	BBh0.11	[M]Reticulum cell sarcoma NOS
cancer	BBH1.00	[M]Myxosarcoma
cancer	BBh2.00	[M]Reticulosarcoma, nodular
cancer	BBj..00	[M]Hodgkin's dis
cancer	BBj0.00	[M]Hodgkin's dis NOS
cancer	BBj0.11	[M]Lymphogranuloma, malignant
cancer	BBj1.00	[M]Hodgkin's dis, lymphocytic predominance
cancer	BBJ1.00	[M]Liposarcoma NOS
cancer	BBJ1.11	[M]Fibroliposarcoma
cancer	BBj1000	[M]Hodgkin,s dis, lymphocytic predominance, diffuse
cancer	BBj1100	[M]Hodgkin,s dis, lymphocytic pred, nodular
cancer	BBj2.00	[M]Hodgkin's dis, mixed cellularity

cancer	BBJ3.00	[M]Liposarcoma, well differentiated type
cancer	BBj4.00	[M]Hodgkin's dis,lymphoc depletion,diffuse fibrosis
cancer	BBJ5.00	[M]Myxoid liposarcoma
cancer	BBJ5.12	[M]Myxoliposarcoma
cancer	BBj6.00	[M]Hodgkin's dis, nodular sclerosis NOS
cancer	BBJ6.00	[M]Round cell liposarcoma
cancer	BBj6000	[M]Hodgkin,s dis, nodular sclerosis, lymphoc predom
cancer	BBj6100	[M]Hodgkin,s dis, nodular sclerosis, mixed cellularity
cancer	BBj6200	[M]Hodgkin,s dis, nodular sclerosis, lymphoc deplet
cancer	BBj7.00	[M]Hodgkin's dis, nodular sclerosis, cellular phase
cancer	BBJ7.00	[M]Pleomorphic liposarcoma
cancer	BBJ8.00	[M]Mixed type liposarcoma
cancer	BBj9.00	[M]Hodgkin's granuloma
cancer	BBJH.00	[M]Dedifferentiated liposarcoma
cancer	BBjz.00	[M]Hodgkin's dis NOS
cancer	BBk..00	[M]Lymphomas, nodular or follicular
cancer	BBk0.00	[M]Malignant lymphoma, nodular NOS
cancer	BBk0.11	[M]Brill - Symmers' dis
cancer	BBk0.12	[M]Follicular lymphosarcoma NOS
cancer	BBk0.13	[M]Giant follicular lymphoma
cancer	BBK0200	[M]Leiomyosarcoma NOS
cancer	BBK0311	[M]Leiomyoblastoma
cancer	BBK0400	[M]Epithelioid leiomyosarcoma
cancer	BBK0700	[M]Myxoid leiomyosarcoma
cancer	BBK1100	[M]Angiomyosarcoma
cancer	BBk2.00	[M]Malignant lymph, centroblastic-centrocytic, follicular
cancer	BBK2.00	[M]Myoma and myosarcoma
cancer	BBK2100	[M]Myosarcoma
cancer	BBk3.00	[M]Malig lymph, lymphoc, well differentiated ,nodular
cancer	BBK3100	[M]Rhabdomyosarcoma NOS
cancer	BBK3200	[M]Pleomorphic rhabdomyosarcoma
cancer	BBK3300	[M]Mixed cell rhabdomyosarcoma
cancer	BBK3600	[M]Embryonal rhabdomyosarcoma
cancer	BBK3611	[M]Sarcoma botryoides
cancer	BBK3700	[M]Alveolar rhabdomyosarcoma
cancer	BBk5.00	[M]Malig lymp, follicular centre cell, cleaved, follicular
cancer	BBk7.00	[M]Malignant lymphoma, centroblastic type, follicular
cancer	BBk8.00	[M]Malig lymp,follicular centre cell,noncleaved,follicular
cancer	BBkz.00	[M]Lymphoma, nodular or follicular NOS
cancer	BBl..00	[M]Mycosis fungoides
cancer	BBl0.00	[M]Mycosis fungoides
cancer	BBL0.00	[M]Endometrial stromal sarcoma
cancer	BBl1.00	[M]Sezary's dis
cancer	BBL4.00	[M]Mixed tumour, malignant, NOS

cancer	BBL7.00	[M]Mixed and stromal renal neoplasms
cancer	BBL7.11	[M]Nephromas and nephroblastomas
cancer	BBL7000	[M]Mesoblastic nephroma
cancer	BBL7100	[M]Nephroblastoma NOS
cancer	BBL7111	[M]Adenosarcoma
cancer	BBL7112	[M]Wilms' tumour
cancer	BBL7200	[M]Epithelial nephroblastoma
cancer	BBL7300	[M]Mesenchymal nephroblastoma
cancer	BBL8.00	[M]Hepatoblastoma
cancer	BBL8.11	[M]Embryonal hepatoma
cancer	BBL9.00	[M]Carcinosarcoma NOS
cancer	BBLA.00	[M]Carcinosarcoma, embryonal type
cancer	BBLA.11	[M]Pneumoblastoma
cancer	BBLC100	[M]Mesenchymoma, malignant
cancer	BBLD.00	[M]Embryonal sarcoma
cancer	BBLE.00	[M]Adenosarcoma
cancer	BBLG.00	[M]Carcinoma in pleomorphic adenoma
cancer	BBLH.00	[M]Rhabdoid sarcoma
cancer	BBLJ.00	[M]Clear cell sarcoma of kidney
cancer	BBLM.00	[M]Pulmonary blastoma
cancer	BBlz.00	[M]Mycosis fungoides NOS
cancer	BBm0.00	[M]Microglioma
cancer	BBM0000	[M]Brenner tumour, borderline malignancy
cancer	BBM0100	[M]Brenner tumour, malignant
cancer	BBm1.00	[M]Malignant histiocytosis
cancer	BBm1.11	[M]Malignant reticulosis
cancer	BBm3.00	[M]Letterer - Siwe dis
cancer	BBm3.12	[M]Acute progressive histiocytosis X
cancer	BBm4.00	[M]True histiocytic lymphoma
cancer	BBm5.00	[M] Peripheral T-cell lymphoma NOS
cancer	BBM8.00	[M]Cystosarcoma phyllodes NOS
cancer	BBm9.00	[M] Monocytoid B-cell lymphoma
cancer	BBM9.00	[M]Cystosarcoma phyllodes, malignant
cancer	BBmA.00	[M] Refractory anaemia with sideroblasts
cancer	BBmB.00	[M]Refract anaemia+excess of blasts with transformation
cancer	BBmC.00	[M] T-gamma lymphoproliferative dis
cancer	BBmD.00	[M] Cutaneous lymphoma
cancer	BBmE.00	[M] Gamma heavy chain dis
cancer	BBmF.00	[M] Angiocentric immunoproliferative lesion
cancer	BBmH.00	[M] Large cell lymphoma
cancer	BBmJ.00	[M] Angioendotheliomatosis
cancer	BBmK.00	[M]Waldenstrom's macroglobulinaemia
cancer	BBmL.00	[M] Refractory anaemia with excess of blasts
cancer	BBn0.00	[M]Plasma cell myeloma

cancer	BBn0.11	[M]Multiple myeloma
cancer	BBn0.12	[M]Myeloma NOS
cancer	BBn0.13	[M]Myelomatosis
cancer	BBn0.14	[M]Plasmacytic myeloma
cancer	BBN1.00	[M]Synovial sarcoma NOS
cancer	BBN2.00	[M]Synovial sarcoma, spindle cell type
cancer	BBn3.00	[M]Plasma cell tumour, malignant
cancer	BBN4.00	[M]Synovial sarcoma, biphasic type
cancer	BBN5.00	[M]Clear cell sarcoma of tendons and aponeuroses
cancer	BBp1.00	[M]Mast cell sarcoma
cancer	BBP1.00	[M]Mesothelioma, malignant
cancer	BBp2.00	[M]Malignant mastocytosis
cancer	BBP3.11	[M]Sarcomatoid mesothelioma
cancer	BBP5.00	[M]Epithelioid mesothelioma, malignant
cancer	BBP7.00	[M]Mesothelioma, biphasic type, malignant
cancer	BBPX.00	[M]Mesothelioma, unspecified
cancer	BBQ1.00	[M]Seminomas
cancer	BBQ1000	[M]Seminoma, anaplastic type
cancer	BBQ1100	[M]Spermatocytic seminoma
cancer	BBQ1z00	[M]Seminoma NOS
cancer	BBQ3.00	[M]Embryonal carcinoma NOS
cancer	BBQ4.00	[M]Endodermal sinus tumour
cancer	BBQ4.11	[M]Infantile embryonal carcinoma
cancer	BBQ4.12	[M]Orchioblastoma
cancer	BBQ4.14	[M]Yolk sac tumour
cancer	BBQ6.00	[M]Gonadoblastoma
cancer	BBQ7200	[M]Teratoma, malignant, NOS
cancer	BBQ7211	[M]Embryonal teratoma
cancer	BBQ7212	[M]Immature teratoma
cancer	BBQ7213	[M]Teratoblastoma, malignant
cancer	BBQ7300	[M]Teratocarcinoma
cancer	BBQ7400	[M]Malignant teratoma, undifferentiated type
cancer	BBQ7500	[M]Malignant teratoma, intermediate type
cancer	BBQ9.00	[M]Dermoid cyst with malignant transformation
cancer	BBQA100	[M]Struma ovarii, malignant
cancer	BBr..00	[M]Leukaemias
cancer	BBr0.00	[M]Leukaemias unspecified
cancer	BBr0000	[M]Leukaemia NOS
cancer	BBr0100	[M]Acute leukaemia NOS
cancer	BBr0111	[M]Blast cell leukaemia
cancer	BBr0112	[M]Blastic leukaemia
cancer	BBr0113	[M]Stem cell leukaemia
cancer	BBr0200	[M]Subacute leukaemia NOS
cancer	BBr0300	[M]Chronic leukaemia NOS

cancer	BBr0400	[M]Aleukaemic leukaemia NOS
cancer	BBr0z00	[M]Leukaemia unspecified, NOS
cancer	BBR1.00	[M]Invasive hydatidiform mole
cancer	BBR1.11	[M]Chorioadenoma
cancer	BBR1.12	[M]Chorioadenoma destruens
cancer	BBR1.13	[M]Invasive mole NOS
cancer	BBr2.00	[M]Lymphoid leukaemias
cancer	BBR2.00	[M]Choriocarcinoma
cancer	BBr2000	[M]Lymphoid leukaemia NOS
cancer	BBr2011	[M]Lymphatic leukaemia
cancer	BBr2100	[M]Acute lymphoid leukaemia
cancer	BBr2300	[M]Chronic lymphoid leukaemia
cancer	BBr2500	[M]Prolymphocytic leukaemia
cancer	BBr2600	[M]Burkitt's cell leukaemia
cancer	BBr2700	[M]Adult T-cell leukaemia/lymphoma
cancer	BBr3.00	[M]Plasma cell leukaemias
cancer	BBR3.00	[M]Choriocarcinoma combined with teratoma
cancer	BBr4.00	[M]Erythroleukaemias
cancer	BBR4.00	[M]Malignant teratoma, trophoblastic
cancer	BBr4000	[M]Erythroleukaemia
cancer	BBr4z00	[M]Erythroleukaemia NOS
cancer	BBR5.00	[M]Partial hydatidiform mole
cancer	BBr6.00	[M]Myeloid leukaemias
cancer	BBR6.00	[M]Placental site trophoblastic tumour
cancer	BBr6000	[M]Myeloid leukaemia NOS
cancer	BBr6011	[M]Granulocytic leukaemia NOS
cancer	BBr6100	[M]Acute myeloid leukaemia
cancer	BBr6200	[M]Subacute myeloid leukaemia
cancer	BBr6300	[M]Chronic myeloid leukaemia
cancer	BBr6311	[M]Naegeli-type monocytic leukaemia
cancer	BBr6600	[M]Acute promyelocytic leukaemia
cancer	BBr6700	[M]Acute myelomonocytic leukaemia
cancer	BBr6800	[M]Chronic myelomonocytic leukaemia
cancer	BBr6900	[M]Juvenile myelomonocytic leukaemia
cancer	BBr6z00	[M]Other myeloid leukaemia NOS
cancer	BBR7.00	[M]Classical hydatidiform mole
cancer	BBr7000	[M]Basophilic leukaemia
cancer	BBr8.00	[M]Eosinophilic leukaemias
cancer	BBR8.00	[M]Complete hydatidiform mole
cancer	BBr8000	[M]Eosinophilic leukaemia
cancer	BBr8z00	[M]Eosinophilic leukaemia NOS
cancer	BBr9000	[M]Monocytic leukaemia NOS
cancer	BBrA.00	[M]Miscellaneous leukaemias
cancer	BBrA100	[M]Megakaryocytic leukaemia

cancer	BBrA111	[M]Thrombocytic leukaemia
cancer	BBrA300	[M]Myeloid sarcoma
cancer	BBrA311	[M]Chloroma
cancer	BBrA312	[M]Granulocytic sarcoma
cancer	BBrA400	[M]Hairy cell leukaemia
cancer	BBrA500	[M]Acute megakaryoblastic leukaemia
cancer	BBrA600	[M]Acute panmyelosis
cancer	BBrA700	[M]Acute myelofibrosis
cancer	BBrAz00	[M]Miscellaneous leukaemia NOS
cancer	BBrz.00	[M]Leukaemia NOS
cancer	BBRz.00	[M]Trophoblastic neoplasm NOS
cancer	BBS..00	[M]Mesonephromas
cancer	BBs..00	[M]Misc myeloproliferative/lymphoproliferative disorders
cancer	BBs0.00	[M]Polycythaemia vera
cancer	BBs0.11	[M]Polycythaemia rubra vera
cancer	BBs1.00	[M]Acute panmyelosis
cancer	BBs2.00	[M]Chronic myeloproliferative dis
cancer	BBs4.00	[M]Idiopathic thrombocythaemia
cancer	BBs5.00	[M]Chronic lymphoproliferative dis
cancer	BBsz.00	[M]Misc myeloproliferative/ lymphoproliferative dis NOS
cancer	BBT1.00	[M]Haemangiosarcoma
cancer	BBT1.11	[M]Angiosarcoma
cancer	BBT7100	[M]Haemangioendothelioma, malignant
cancer	BBTA.00	[M]Kaposi's sarcoma
cancer	BBTD200	[M]Haemangiopericytoma, malignant
cancer	BBTK.00	[M]Epithelioid haemangioendothelioma, malignant
cancer	BBU1.00	[M]Lymphangiosarcoma
cancer	BBV..00	[M]Osteomas and osteosarcomas
cancer	BBv..00	[M]Myelodysplastic synde
cancer	BBV..11	[M]Juxtacortical osteogenic sarcoma
cancer	BBV..12	[M]Parosteal osteosarcoma
cancer	BBV..13	[M]Periosteal osteogenic sarcoma
cancer	BBv0.00	[M]Monocytoid B-cell lymphoma
cancer	BBV1.00	[M]Osteosarcoma NOS
cancer	BBV1.11	[M]Osteoblastic sarcoma
cancer	BBV1.12	[M]Osteochondrosarcoma
cancer	BBV1.13	[M]Osteogenic sarcoma NOS
cancer	BBV2.00	[M]Chondroblastic osteosarcoma
cancer	BBv2.00	[M]AngiocentricT-cell lymphoma
cancer	BBV3.00	[M]Fibroblastic osteosarcoma
cancer	BBV4.00	[M]Telangiectatic osteosarcoma
cancer	BBV5.00	[M]Osteosarcoma in Paget's dis of bone
cancer	BBV9.00	[M]Myxoid chondrosarcoma
cancer	BBVA.00	[M] Small cell osteosarcoma

cancer	BBW4.00	[M]Chondrosarcoma NOS
cancer	BBW4.11	[M]Fibrochondrosarcoma
cancer	BBW6.00	[M]Juxtacortical chondrosarcoma
cancer	BBW8.00	[M]Chondroblastoma, malignant
cancer	BBW9.00	[M]Mesenchymal chondrosarcoma
cancer	BBX1.00	[M]Giant cell tumour of bone, malignant
cancer	BBX1.11	[M]Giant cell bone sarcoma
cancer	BBX1.12	[M]Osteoclastoma, malignant
cancer	BBX3.00	[M]Malignant giant cell tumour of soft parts
cancer	BBY0.00	[M]Ewing's sarcoma
cancer	BBY0.11	[M]Endothelial bone sarcoma
cancer	BBZ2.00	[M]Odontogenic tumour, malignant
cancer	BBZ2.11	[M]Intraosseous carcinoma
cancer	BBZC.00	[M]Ameloblastic odontosarcoma
cancer	BBZG.00	[M]Ameloblastoma, malignant
cancer	BBZG.11	[M]Adamantinoma, malignant
cancer	BBZN.00	[M]Ameloblastic fibrosarcoma
cancer	BBZN.11	[M]Odontogenic fibrosarcoma
cancer	Byu0.00	[X]Malignant neoplasm of lip, oral cavity and pharynx
cancer	Byu1.00	[X]Malignant neoplasm of digestive organs
cancer	Byu1100	[X]Other specified carcinomas of liver
cancer	Byu1200	[X]Malignant neoplasm of intestinal tract, part unsp
cancer	Byu1300	[X]Malignant neoplsm/ill-defin sites within digestive syst
cancer	Byu2.00	[X]Malignant neoplasm of respiratory intrathoracic orga
cancer	Byu2000	[X]Malignant neoplasm of bronchus or lung, unspecified
cancer	Byu2100	[X]Malignant neoplasm/overlap les/heart,mediast+pleura
cancer	Byu2300	[X]Malign neopl/overlapping les/resp+intrathoracic organ
cancer	Byu2400	[X]Malignant neoplasm/ill-defined sites within resp syst
cancer	Byu2500	[X]Malignant neoplasm of mediastinum, part unspecified
cancer	Byu3.00	[X]Malignant neoplasm of bone and articular cartilage
cancer	Byu3100	[X]Malig neoplasm/bones+articular cartilage/limb,unspfd
cancer	Byu3200	[X]Malignant neopl/overlap lesion/bone+articulr cartilage
cancer	Byu3300	[X]Malignant neoplasm/bone+articular cartilage, unspec
cancer	Byu4000	[X]Malignant melanoma of other+unspecified parts face
cancer	Byu4100	[X]Malignant melanoma of skin, unspecified
cancer	Byu5.00	[X]Malignant neoplasm of mesothelial and soft tissue
cancer	Byu5000	[X]Mesothelioma of other sites
cancer	Byu5011	[X]Mesothelioma of lung
cancer	Byu5100	[X]Mesothelioma, unspecified
cancer	Byu5300	[X]Kaposi's sarcoma, unspecified
cancer	Byu5400	[X]Malign neoplasm/peripheral nerves trunk,unspecified
cancer	Byu5500	[X]Mal neopl/overlap les/periph nerv+autono nerv syst
cancer	Byu5700	[X]Malignant neoplasm of peritoneum, unspecified
cancer	Byu5800	[X]Mal neoplasm/connective+soft tissue of trunk,unspec

cancer	Byu5900	[X]Malignant neoplasm/connective + soft tissue,unspec
cancer	Byu5B00	[X]Kaposi's sarcoma of other sites
cancer	Byu6.00	[X]Malignant neoplasm of breast
cancer	Byu7.00	[X]Malignant neoplasm of female genital organs
cancer	Byu7000	[X]Malignant neoplasm of uterine adnexa, unspecified
cancer	Byu7100	[X]Malign neoplasm/other specified female genital organs
cancer	Byu7300	[X]Malignant neoplasm of female genital organ, unspec
cancer	Byu8.00	[X]Malignant neoplasm of male genital organs
cancer	Byu8000	[X]Malignant neopl/other specified male genital organs
cancer	Byu8200	[X]Malignant neoplasm of male genital organ, unspecified
cancer	Byu9.00	[X]Malignant neoplasm of urinary tract
cancer	Byu9000	[X]Malignant neoplasm of urinary organ, unspecified
cancer	ByuA.00	[X]Malignant neoplasm of eye, brain and other parts cent
cancer	ByuA000	[X]Malignant neoplasm/other and unspec cranial nerves
cancer	ByuA100	[X]Malignant neoplasm/central nervous system, unspec
cancer	ByuA200	[X]Malignant neoplasm of meninges, unspecified
cancer	ByuA300	[X]Malig neopl, overlap lesion brain & other part of CNS
cancer	ByuB.00	[X]Malignant neoplasm of thyroid other endocrine glands
cancer	ByuB100	[X]Malignant neoplasm of endocrine gland, unspecified
cancer	ByuC.00	[X]Malignant neoplasm of ill-defined, secondary unspeci
cancer	ByuC000	[X]Malignant neoplasm of other specified sites
cancer	ByuC100	[X]Malig neoplasm/overlap lesion/other+ill-defined sites
cancer	ByuC800	[X]Malignant neoplasm without specification of site
cancer	ByuD.00	[X]Malignant neoplasms of lymphoid, haematopoietic rela
cancer	ByuD000	[X]Other Hodgkin's dis
cancer	ByuD100	[X]Other types of follicular non-Hodgkin's lymphoma
cancer	ByuD200	[X]Other types of diffuse non-Hodgkin's lymphoma
cancer	ByuD300	[X]Other specified types of non-Hodgkin's lymphoma
cancer	ByuD400	[X]Other malignant immunoproliferative diss
cancer	ByuD500	[X]Other lymphoid leukaemia
cancer	ByuD600	[X]Other myeloid leukaemia
cancer	ByuD700	[X]Other monocytic leukaemia
cancer	ByuD800	[X]Other specified leukaemias
cancer	ByuD900	[X]Other leukaemia of unspecified cell type
cancer	ByuDA00	[X]Oth spcf mal neoplsm/lymphoid,haematopo+rltd tissue
cancer	ByuDB00	[X]Mal neopl/lymphoid,haematopo+related tissu,unspcf
cancer	ByuDC00	[X]Diffuse non-Hodgkin's lymphoma, unspecified
cancer	ByuDE00	[X]Unspecified B-cell non-Hodgkin's lymphoma
cancer	ByuDF00	[X]Non-Hodgkin's lymphoma, unspecified type
cancer	ByuDF11	[X]Non-Hodgkin's lymphoma NOS
cancer	ByuE.00	[X]Malignant neoplasms/independent (primary) mult sites
cancer	ByuE000	[X]Malignant neoplasms/independent(primary)mult sites
cancer	ByuF.00	[X]In situ neoplasms
cancer	ByuF100	[X]Carcinoma in situ of other specified digestive organs

cancer	ByuF300	[X]Carcinoma in situ of other parts of respiratory system
cancer	ByuF600	[X]Melanoma in situ of other sites
cancer	ByuFA00	[X]Carcinoma in situ of other parts of cervix
cancer	ByuFC00	[X]Carcinoma in situ of oth+unspecified male genital orga
cancer	ByuFF00	[X]Melanoma in situ, unspecified
cancer	ByuFG00	[X]Other carcinoma in situ of breast
cancer	ByuHD00	[X]Myelodysplastic synde, unspecified
cancer	F373.00	Polyneuropathy in malignant dis
cancer	F381100	Myasthenic synde due to other malignancy
cancer	F396200	Myopathy due to malignant dis
cancer	H51y700	Malignant pleural effusion
cancer	K01w100	Drash synde
cancer	K01w112	Wilms' tumour+nephrotic synde+pseudohermaphrodi
cancer	N330900	Osteoporosis in multiple myelomatosis
cerebrovascular dis	1477	H/O: cerebrovascular dis
cerebrovascular dis	A94y600	Rupture of syphilitic cerebral aneurysm
cerebrovascular dis	E030400	Acute confusional state, of cerebrovascular origin
cerebrovascular dis	E031400	Subacute confusional state, of cerebrovascular origin
cerebrovascular dis	F11x200	Cerebral degeneration due to cerebrovascular dis
cerebrovascular dis	F423600	Amaurosis fugax
cerebrovascular dis	F423700	Retinal transient arterial occlusion NOS
cerebrovascular dis	F481400	Other transient visual loss
cerebrovascular dis	Fyu5500	[X]Other transnt cerebral ischaemic attacks+related synd
cerebrovascular dis	Fyu5700	[X]Other vascular synd/brain in cerebrovasculr diss
cerebrovascular dis	G6...00	Cerebrovascular dis
cerebrovascular dis	G60..00	Subarachnoid haemorrhage
cerebrovascular dis	G600.00	Ruptured berry aneurysm
cerebrovascular dis	G601.00	Subarachnoid haemorrh from carotid siphon bifurcation
cerebrovascular dis	G602.00	Subarachnoid haemorrhage from middle cerebral artery
cerebrovascular dis	G603.00	Subarachnoid haemorrh frm ant communicating artery
cerebrovascular dis	G604.00	Subarachnoid haemorrh from post communicating artery
cerebrovascular dis	G605.00	Subarachnoid haemorrhage from basilar artery
cerebrovascular dis	G606.00	Subarachnoid haemorrhage from vertebral artery
cerebrovascular dis	G60X.00	Subarachnoid haemorrh from intracranial artery, unspecif
cerebrovascular dis	G60z.00	Subarachnoid haemorrhage NOS
cerebrovascular dis	G61..00	Intracerebral haemorrhage
cerebrovascular dis	G61..11	CVA-cerebrovascular accid due to intracerebral haemorrh
cerebrovascular dis	G61..12	Stroke due to intracerebral haemorrhage
cerebrovascular dis	G610.00	Cortical haemorrhage
cerebrovascular dis	G611.00	Internal capsule haemorrhage
cerebrovascular dis	G612.00	Basal nucleus haemorrhage
cerebrovascular dis	G613.00	Cerebellar haemorrhage
cerebrovascular dis	G614.00	Pontine haemorrhage
cerebrovascular dis	G615.00	Bulbar haemorrhage

cerebrovascular dis	G616.00	External capsule haemorrhage
cerebrovascular dis	G617.00	Intracerebral haemorrhage, intraventricular
cerebrovascular dis	G618.00	Intracerebral haemorrhage, multiple localized
cerebrovascular dis	G619.00	Lobar cerebral haemorrhage
cerebrovascular dis	G61X.00	Intracerebral haemorrhage in hemisphere, unspecified
cerebrovascular dis	G61X000	Left sided intracerebral haemorrhage, unspecified
cerebrovascular dis	G61X100	Right sided intracerebral haemorrhage, unspecified
cerebrovascular dis	G61z.00	Intracerebral haemorrhage NOS
cerebrovascular dis	G62..00	Other and unspecified intracranial haemorrhage
cerebrovascular dis	G620.00	Extradural haemorrhage - nontraumatic
cerebrovascular dis	G621.00	Subdural haemorrhage - nontraumatic
cerebrovascular dis	G622.00	Subdural haematoma - nontraumatic
cerebrovascular dis	G623.00	Subdural haemorrhage NOS
cerebrovascular dis	G62z.00	Intracranial haemorrhage NOS
cerebrovascular dis	G63..00	Precerebral arterial occlusion
cerebrovascular dis	G63..11	Infarction - precerebral
cerebrovascular dis	G63..12	Stenosis of precerebral arteries
cerebrovascular dis	G630.00	Basilar artery occlusion
cerebrovascular dis	G631.00	Carotid artery occlusion
cerebrovascular dis	G631.11	Stenosis, carotid artery
cerebrovascular dis	G631.12	Thrombosis, carotid artery
cerebrovascular dis	G632.00	Vertebral artery occlusion
cerebrovascular dis	G633.00	Multiple and bilateral precerebral arterial occlusion
cerebrovascular dis	G634.00	Carotid artery stenosis
cerebrovascular dis	G63y.00	Other precerebral artery occlusion
cerebrovascular dis	G63y000	Cerebral infarct due to thrombosis of precerebral arteries
cerebrovascular dis	G63y100	Cerebral infarction due to embolism precerebral arteries
cerebrovascular dis	G63z.00	Precerebral artery occlusion NOS
cerebrovascular dis	G64..00	Cerebral arterial occlusion
cerebrovascular dis	G64..11	CVA - cerebral artery occlusion
cerebrovascular dis	G64..12	Infarction - cerebral
cerebrovascular dis	G64..13	Stroke due to cerebral arterial occlusion
cerebrovascular dis	G640.00	Cerebral thrombosis
cerebrovascular dis	G640000	Cerebral infarction due to thrombosis of cerebral arteries
cerebrovascular dis	G641.00	Cerebral embolism
cerebrovascular dis	G641.11	Cerebral embolus
cerebrovascular dis	G641000	Cerebral infarction due to embolism of cerebral arteries
cerebrovascular dis	G64z.00	Cerebral infarction NOS
cerebrovascular dis	G64z.11	Brainstem infarction NOS
cerebrovascular dis	G64z.12	Cerebellar infarction
cerebrovascular dis	G64z000	Brainstem infarction
cerebrovascular dis	G64z100	Wallenberg synde
cerebrovascular dis	G64z111	Lateral medullary synde
cerebrovascular dis	G64z200	Left sided cerebral infarction

cerebrovascular dis	G64z300	Right sided cerebral infarction
cerebrovascular dis	G64z400	Infarction of basal ganglia
cerebrovascular dis	G65..00	Transient cerebral ischaemia
cerebrovascular dis	G65..11	Drop attack
cerebrovascular dis	G65..12	Transient ischaemic attack
cerebrovascular dis	G65..13	Vertebro-basilar insufficiency
cerebrovascular dis	G650.00	Basilar artery synde
cerebrovascular dis	G650.11	Insufficiency - basilar artery
cerebrovascular dis	G651.00	Vertebral artery synde
cerebrovascular dis	G651000	Vertebro-basilar artery synde
cerebrovascular dis	G652.00	Subclavian steal synde
cerebrovascular dis	G653.00	Carotid artery synde hemispheric
cerebrovascular dis	G654.00	Multiple and bilateral precerebral artery syndes
cerebrovascular dis	G655.00	Transient global amnesia
cerebrovascular dis	G656.00	Vertebrobasilar insufficiency
cerebrovascular dis	G657.00	Carotid territory transient ischaemic attack
cerebrovascular dis	G65y.00	Other transient cerebral ischaemia
cerebrovascular dis	G65z.00	Transient cerebral ischaemia NOS
cerebrovascular dis	G65z000	Impending cerebral ischaemia
cerebrovascular dis	G65z100	Intermittent cerebral ischaemia
cerebrovascular dis	G65zz00	Transient cerebral ischaemia NOS
cerebrovascular dis	G66..00	Stroke and cerebrovascular accident unspecified
cerebrovascular dis	G66..11	CVA unspecified
cerebrovascular dis	G66..12	Stroke unspecified
cerebrovascular dis	G66..13	CVA - Cerebrovascular accident unspecified
cerebrovascular dis	G660.00	Middle cerebral artery synde
cerebrovascular dis	G661.00	Anterior cerebral artery synde
cerebrovascular dis	G662.00	Posterior cerebral artery synde
cerebrovascular dis	G663.00	Brain stem stroke synde
cerebrovascular dis	G664.00	Cerebellar stroke synde
cerebrovascular dis	G665.00	Pure motor lacunar synde
cerebrovascular dis	G666.00	Pure sensory lacunar synde
cerebrovascular dis	G667.00	Left sided CVA
cerebrovascular dis	G668.00	Right sided CVA
cerebrovascular dis	G669.00	Cerebral palsy, not congenital or infantile, acute
cerebrovascular dis	G67..00	Other cerebrovascular dis
cerebrovascular dis	G670.00	Cerebral atherosclerosis
cerebrovascular dis	G670.11	Precerebral atherosclerosis
cerebrovascular dis	G671.00	Generalised ischaemic cerebrovascular dis NOS
cerebrovascular dis	G671000	Acute cerebrovascular insufficiency NOS
cerebrovascular dis	G671100	Chronic cerebral ischaemia
cerebrovascular dis	G671z00	Generalised ischaemic cerebrovascular dis NOS
cerebrovascular dis	G673.00	Cerebral aneurysm, nonruptured
cerebrovascular dis	G673000	Dissection of cerebral arteries, nonruptured

cerebrovascular dis	G673100	Carotico-cavernous sinus fistula
cerebrovascular dis	G673200	Carotid artery dissection
cerebrovascular dis	G673300	Vertebral artery dissection
cerebrovascular dis	G674.00	Cerebral arteritis
cerebrovascular dis	G674000	Cerebral amyloid angiopathy
cerebrovascular dis	G675.00	Moyamoya dis
cerebrovascular dis	G676.00	Nonpyogenic venous sinus thrombosis
cerebrovascular dis	G676000	Cereb infarct due cerebral ven thrombosis, nonpyogenic
cerebrovascular dis	G677.00	Occlusion/stenosis cerebral arts not result cerebral infarct
cerebrovascular dis	G677000	Occlusion and stenosis of middle cerebral artery
cerebrovascular dis	G677100	Occlusion and stenosis of anterior cerebral artery
cerebrovascular dis	G677200	Occlusion and stenosis of posterior cerebral artery
cerebrovascular dis	G677300	Occlusion and stenosis of cerebellar arteries
cerebrovascular dis	G677400	Occlusion+stenosis of multiple and bilat cerebral arteries
cerebrovascular dis	G678.00	Cereb autosom dom arteriop subcort infarcts leukoenc
cerebrovascular dis	G679.00	Small vessel cerebrovascular dis
cerebrovascular dis	G67A.00	Cerebral vein thrombosis
cerebrovascular dis	G67y.00	Other cerebrovascular dis OS
cerebrovascular dis	G67z.00	Other cerebrovascular dis NOS
cerebrovascular dis	G68..00	Late effects of cerebrovascular dis
cerebrovascular dis	G680.00	Sequelae of subarachnoid haemorrhage
cerebrovascular dis	G681.00	Sequelae of intracerebral haemorrhage
cerebrovascular dis	G682.00	Sequelae of other nontraumatic intracranial haemorrhage
cerebrovascular dis	G683.00	Sequelae of cerebral infarction
cerebrovascular dis	G68W.00	Sequelae/other + unspecified cerebrovascular diss
cerebrovascular dis	G68X.00	Sequelae of stroke,not specfd as h'morrhage or infarction
cerebrovascular dis	G6W..00	Cereb infarct due unsp occlus/stenos precerebr arteries
cerebrovascular dis	G6X..00	Cerebrl infarctn due/unspcf occlusn or sten/cerebrl artrs
cerebrovascular dis	G6y..00	Other specified cerebrovascular dis
cerebrovascular dis	G6z..00	Cerebrovascular dis NOS
cerebrovascular dis	Gyu6.00	[X]Cerebrovascular diss
cerebrovascular dis	Gyu6000	[X]Subarachnoid haemorrh fr other intracranial arteries
cerebrovascular dis	Gyu6100	[X]Other subarachnoid haemorrhage
cerebrovascular dis	Gyu6200	[X]Other intracerebral haemorrhage
cerebrovascular dis	Gyu6300	[X]Cerebrl infarctn due/unspcf occlusn/sten/cerebrl artrs
cerebrovascular dis	Gyu6400	[X]Other cerebral infarction
cerebrovascular dis	Gyu6500	[X]Occlusion and stenosis of other precerebral arteries
cerebrovascular dis	Gyu6600	[X]Occlusion and stenosis of other cerebral arteries
cerebrovascular dis	Gyu6A00	[X]Other cerebrovascular disorders in diss CE
cerebrovascular dis	Gyu6E00	[X]Subarachnoid haemorrh frm intracranial artery, unspec
cerebrovascular dis	Gyu6F00	[X]Intracerebral haemorrhage in hemisphere, unspecified
cerebrovascular dis	Gyu6G00	[X]Cereb infarct due unsp occlus/stenos precerebr arter
cerebrovascular dis	L440.00	Cerebrovascular disorders in the puerperium
cerebrovascular dis	L440.11	CVA - cerebrovascular accident in the puerperium

cerebrovascular dis	L440.12	Stroke in the puerperium
cerebrovascular dis	L440000	Puerperal cerebrovascular disorder unspecified
cerebrovascular dis	L440100	Puerperal cerebrovascular disorder - delivered
cerebrovascular dis	L440300	Puerperal cerebrovascular disorder with antenatal comp
cerebrovascular dis	R014.00	[D]Transient paralysis of a limb
cerebrovascular dis	R014000	[D]Transient monoplegia NOS
cerebrovascular dis	R014z00	[D]Transient limb paralysis NOS
cerebrovascular dis	ZV12511	[V]Personal history of stroke
cerebrovascular dis	ZV12512	[V]Personal history of cerebrovascular accident (CVA)
cerebrovascular dis	ZV12D00	[V]Personal history of transient ischaemic attack
Chronic pulmonary dis	173A.00	Exercise induced asthma
Chronic pulmonary dis	173c.00	Occupational asthma
Chronic pulmonary dis	173d.00	Work aggravated asthma
Chronic pulmonary dis	178..00	Asthma trigger
Chronic pulmonary dis	1780	Aspirin induced asthma
Chronic pulmonary dis	1781	Asthma trigger - pollen
Chronic pulmonary dis	1782	Asthma trigger - tobacco smoke
Chronic pulmonary dis	1783	Asthma trigger - warm air
Chronic pulmonary dis	1784	Asthma trigger - emotion
Chronic pulmonary dis	1785	Asthma trigger - damp
Chronic pulmonary dis	1786	Asthma trigger - animals
Chronic pulmonary dis	1787	Asthma trigger - seasonal
Chronic pulmonary dis	1788	Asthma trigger - cold air
Chronic pulmonary dis	1789	Asthma trigger - respiratory infection
Chronic pulmonary dis	178A.00	Asthma trigger - airborne dust
Chronic pulmonary dis	178B.00	Asthma trigger - exercise
Chronic pulmonary dis	1O2..00	Asthma confirmed
Chronic pulmonary dis	663d.00	Emergency asthma admission since last appointment
Chronic pulmonary dis	663e.00	Asthma restricts exercise
Chronic pulmonary dis	6.63E+02	Asthma sometimes restricts exercise
Chronic pulmonary dis	663f.00	Asthma never restricts exercise
Chronic pulmonary dis	663h.00	Asthma - currently dormant
Chronic pulmonary dis	663j.00	Asthma - currently active
Chronic pulmonary dis	663m.00	Asthma accidt and emergency attendance since last visit
Chronic pulmonary dis	663n.00	Asthma treatment compliance satisfactory
Chronic pulmonary dis	663N.00	Asthma disturbing sleep
Chronic pulmonary dis	663N000	Asthma causing night waking
Chronic pulmonary dis	663N100	Asthma disturbs sleep weekly
Chronic pulmonary dis	663N200	Asthma disturbs sleep frequently
Chronic pulmonary dis	663O.00	Asthma not disturbing sleep
Chronic pulmonary dis	663O000	Asthma never disturbs sleep
Chronic pulmonary dis	663p.00	Asthma treatment compliance unsatisfactory
Chronic pulmonary dis	663P.00	Asthma limiting activities
Chronic pulmonary dis	663P000	Asthma limits activities 1 to 2 times per month

Chronic pulmonary dis	663P100	Asthma limits activities 1 to 2 times per week
Chronic pulmonary dis	663P200	Asthma limits activities most days
Chronic pulmonary dis	663Q.00	Asthma not limiting activities
Chronic pulmonary dis	663q.00	Asthma daytime symptoms
Chronic pulmonary dis	663r.00	Asthma causes night symptoms 1 to 2 times per month
Chronic pulmonary dis	663s.00	Asthma never causes daytime symptoms
Chronic pulmonary dis	663t.00	Asthma causes daytime symptoms 1 to 2 times per month
Chronic pulmonary dis	663u.00	Asthma causes daytime symptoms 1 to 2 times per week
Chronic pulmonary dis	663U.00	Asthma management plan given
Chronic pulmonary dis	663V.00	Asthma severity
Chronic pulmonary dis	663v.00	Asthma causes daytime symptoms most days
Chronic pulmonary dis	663V000	Occasional asthma
Chronic pulmonary dis	663V100	Mild asthma
Chronic pulmonary dis	663V200	Moderate asthma
Chronic pulmonary dis	663V300	Severe asthma
Chronic pulmonary dis	663w.00	Asthma limits walking up hills or stairs
Chronic pulmonary dis	663W.00	Asthma prophylactic medication used
Chronic pulmonary dis	663x.00	Asthma limits walking on the flat
Chronic pulmonary dis	663y.00	Number of asthma exacerbations in past year
Chronic pulmonary dis	66Y5.00	Change in asthma management plan
Chronic pulmonary dis	66Y9.00	Step up change in asthma management plan
Chronic pulmonary dis	66YA.00	Step down change in asthma management plan
Chronic pulmonary dis	66YC.00	Absent from work or school due to asthma
Chronic pulmonary dis	66YJ.00	Asthma annual review
Chronic pulmonary dis	66YK.00	Asthma follow-up
Chronic pulmonary dis	66Yp.00	Asthma review using Roy Colleg of Physicians three quest
Chronic pulmonary dis	66YP.00	Asthma night-time symptoms
Chronic pulmonary dis	66Yq.00	Asthma causes night time symptoms 1-2 times per week
Chronic pulmonary dis	66Yr.00	Asthma causes symptoms most nights
Chronic pulmonary dis	66Ys.00	Asthma never causes night symptoms
Chronic pulmonary dis	8791	Further asthma - drug prevent.
Chronic pulmonary dis	8793	Asthma control step 0
Chronic pulmonary dis	8794	Asthma control step 1
Chronic pulmonary dis	8795	Asthma control step 2
Chronic pulmonary dis	8796	Asthma control step 3
Chronic pulmonary dis	8797	Asthma control step 4
Chronic pulmonary dis	8798	Asthma control step 5
Chronic pulmonary dis	8B3j.00	Asthma medication review
Chronic pulmonary dis	8CR0.00	Asthma clinical management plan
Chronic pulmonary dis	9N1d.00	Seen in asthma clinic
Chronic pulmonary dis	H061400	Obliterating fibrous bronchiolitis
Chronic pulmonary dis	H312300	Bronchiolitis obliterans
Chronic pulmonary dis	H32y100	Atrophic (senile) emphysema
Chronic pulmonary dis	H33..00	Asthma

Chronic pulmonary dis	H33..11	Bronchial asthma
Chronic pulmonary dis	H330.00	Extrinsic (atopic) asthma
Chronic pulmonary dis	H330.11	Allergic asthma
Chronic pulmonary dis	H330.12	Childhood asthma
Chronic pulmonary dis	H330.13	Hay fever with asthma
Chronic pulmonary dis	H330.14	Pollen asthma
Chronic pulmonary dis	H330000	Extrinsic asthma without status asthmaticus
Chronic pulmonary dis	H330011	Hay fever with asthma
Chronic pulmonary dis	H330100	Extrinsic asthma with status asthmaticus
Chronic pulmonary dis	H330111	Extrinsic asthma with asthma attack
Chronic pulmonary dis	H330z00	Extrinsic asthma NOS
Chronic pulmonary dis	H331.00	Intrinsic asthma
Chronic pulmonary dis	H331.11	Late onset asthma
Chronic pulmonary dis	H331000	Intrinsic asthma without status asthmaticus
Chronic pulmonary dis	H331100	Intrinsic asthma with status asthmaticus
Chronic pulmonary dis	H331111	Intrinsic asthma with asthma attack
Chronic pulmonary dis	H331z00	Intrinsic asthma NOS
Chronic pulmonary dis	H332.00	Mixed asthma
Chronic pulmonary dis	H334.00	Brittle asthma
Chronic pulmonary dis	H33z.00	Asthma unspecified
Chronic pulmonary dis	H33z.11	Hyperreactive airways dis
Chronic pulmonary dis	H33z000	Status asthmaticus NOS
Chronic pulmonary dis	H33z011	Severe asthma attack
Chronic pulmonary dis	H33z100	Asthma attack
Chronic pulmonary dis	H33z111	Asthma attack NOS
Chronic pulmonary dis	H33z200	Late-onset asthma
Chronic pulmonary dis	H33zz00	Asthma NOS
Chronic pulmonary dis	H33zz11	Exercise induced asthma
Chronic pulmonary dis	H33zz12	Allergic asthma NEC
Chronic pulmonary dis	H33zz13	Allergic bronchitis NEC
Chronic pulmonary dis	H34..00	Bronchiectasis
Chronic pulmonary dis	H340.00	Recurrent bronchiectasis
Chronic pulmonary dis	H341.00	Post-infective bronchiectasis
Chronic pulmonary dis	H34z.00	Bronchiectasis NOS
Chronic pulmonary dis	H35y600	Sequoiosis (red-cedar asthma)
Chronic pulmonary dis	H35y700	Wood asthma
Chronic pulmonary dis	H4...11	Pneumoconioses
Chronic pulmonary dis	H4...12	Occupational lung dis
Chronic pulmonary dis	H40..00	Coal workers' pneumoconiosis
Chronic pulmonary dis	H41..00	Asbestosis
Chronic pulmonary dis	H410.00	Pleural plaque dis due to asbestosis
Chronic pulmonary dis	H410.11	Asbestos-induced pleural plaque
Chronic pulmonary dis	H41z.00	Asbestosis NOS
Chronic pulmonary dis	H42..00	Silica and silicate pneumoconiosis

Chronic pulmonary dis	H420.00	Talc pneumoconiosis
Chronic pulmonary dis	H421.00	Simple silicosis
Chronic pulmonary dis	H422.00	Complicated silicosis
Chronic pulmonary dis	H423.00	Massive silicotic fibrosis
Chronic pulmonary dis	H42z.00	Silica pneumoconiosis NOS
Chronic pulmonary dis	H43..00	Pneumoconiosis due to other inorganic dust
Chronic pulmonary dis	H431.00	Bauxite fibrosis of lung
Chronic pulmonary dis	H432.00	Berylliosis
Chronic pulmonary dis	H433.00	Graphite fibrosis of lung
Chronic pulmonary dis	H434.00	Siderosis
Chronic pulmonary dis	H435.00	Stannosis
Chronic pulmonary dis	H43z.00	Pneumoconiosis due to inorganic dust NOS
Chronic pulmonary dis	H44..00	Pneumopathy due to inhalation of other dust
Chronic pulmonary dis	H440.00	Byssinosis
Chronic pulmonary dis	H441.00	Cannabinosis
Chronic pulmonary dis	H44z.00	Pneumopathy due to inhalation of other dust NOS
Chronic pulmonary dis	H45..00	Pneumoconiosis NOS
Chronic pulmonary dis	H450.00	Pneumoconiosis associated with tuberculosis
Chronic pulmonary dis	H464.00	Chronic respiratory conditions due to chemical fumes
Chronic pulmonary dis	H464000	Chronic emphysema due to chemical fumes
Chronic pulmonary dis	H464100	Obliterative bronchiolitis due to chemical fumes
Chronic pulmonary dis	H464200	Chronic pulmonary fibrosis due to chemical fumes
Chronic pulmonary dis	H464z00	Chronic respiratory conditions due to chemical fumes
Chronic pulmonary dis	H47y000	Detergent asthma
Chronic pulmonary dis	H4y1.00	Chronic pulmonary radiation dis
Chronic pulmonary dis	H4y1000	Chronic pulmonary fibrosis following radiation
Chronic pulmonary dis	H4y1z00	Chronic pulmonary radiation dis NOS
Chronic pulmonary dis	H4y2100	Chronic drug-induced interstitial lung disorders
Chronic pulmonary dis	H560.00	Pulmonary alveolar proteinosis
Chronic pulmonary dis	H561.00	Idiopathic pulmonary haemosiderosis
Chronic pulmonary dis	H562.00	Pulmonary alveolar microlithiasis
Chronic pulmonary dis	H563.00	Idiopathic fibrosing alveolitis
Chronic pulmonary dis	H563.11	Hamman - Rich synde
Chronic pulmonary dis	H563.12	Cryptogenic fibrosing alveolitis
Chronic pulmonary dis	H563.13	Idiopathic pulmonary fibrosis
Chronic pulmonary dis	H563100	Diffuse pulmonary fibrosis
Chronic pulmonary dis	H563200	Pulmonary fibrosis
Chronic pulmonary dis	H563300	Usual interstitial pneumonitis
Chronic pulmonary dis	H563z00	Idiopathic fibrosing alveolitis NOS
Chronic pulmonary dis	H564.00	Bronchiolitis obliterans organising pneumonia
Chronic pulmonary dis	H564.11	Cryptogenic organising pneumonia
Chronic pulmonary dis	H56y000	Endogenous lipoid pneumonia
Chronic pulmonary dis	H581.00	Interstitial emphysema
Chronic pulmonary dis	H582.00	Compensatory emphysema

Chronic pulmonary dis	H583200	Eosinophilic bronchitis
Chronic pulmonary dis	H591.00	Chronic respiratory failure
Chronic pulmonary dis	H593.00	Chronic type 2 respiratory failure
Chronic pulmonary dis	Hyu3.00	[X]Chronic lower respiratory diss
Chronic pulmonary dis	Hyu4000	[X]Pneumoconiosis due to other dust containing silica
Chronic pulmonary dis	Hyu4800	[X]Chronic+other pulmonary manifestations due radiation
Chronic pulmonary dis	Hyu5000	[X]Other interstitial pulmonary diss with fibrosis
Cong heart failure	1O1..00	Heart failure confirmed
Cong heart failure	33BA.00	Impaired left ventricular function
Cong heart failure	585f.00	Echocardiogram shows left ventricular systolic dysfunct
Cong heart failure	585g.00	Echocardiogram shows left ventricular diastolic dysfunct
Cong heart failure	662f.00	New York Heart Association classification - class I
Cong heart failure	662g.00	New York Heart Association classification - class II
Cong heart failure	662h.00	New York Heart Association classification - class III
Cong heart failure	662i.00	New York Heart Association classification - class IV
Cong heart failure	662p.00	Heart failure 6 month review
Cong heart failure	662T.00	Cong heart failure monitoring
Cong heart failure	662W.00	Heart failure annual review
Cong heart failure	8B29.00	Cardiac failure therapy
Cong heart failure	8H2S.00	Admit heart failure emergency
Cong heart failure	8HBE.00	Heart failure follow-up
Cong heart failure	9N0k.00	Seen in heart failure clinic
Cong heart failure	9N2p.00	Seen by community heart failure nurse
Cong heart failure	G41..00	Chronic pulmonary heart dis
Cong heart failure	G41y.00	Other chronic pulmonary heart dis
Cong heart failure	G41yz00	Other chronic pulmonary heart dis NOS
Cong heart failure	G41z.00	Chronic pulmonary heart dis NOS
Cong heart failure	G41z.11	Chronic cor pulmonale
Cong heart failure	G58..00	Heart failure
Cong heart failure	G58..11	Cardiac failure
Cong heart failure	G580.00	Cong heart failure
Cong heart failure	G580.11	Cong cardiac failure
Cong heart failure	G580.12	Right heart failure
Cong heart failure	G580.13	Right ventricular failure
Cong heart failure	G580.14	Biventricular failure
Cong heart failure	G580000	Acute Cong heart failure
Cong heart failure	G580100	Chronic Cong heart failure
Cong heart failure	G580200	Decompensated cardiac failure
Cong heart failure	G580300	Compensated cardiac failure
Cong heart failure	G580400	Cong heart failure due to valvular dis
Cong heart failure	G581.00	Left ventricular failure
Cong heart failure	G581.11	Asthma - cardiac
Cong heart failure	G581.12	Pulmonary oedema - acute
Cong heart failure	G581.13	Impaired left ventricular function

Cong heart failure	G581000	Acute left ventricular failure
Cong heart failure	G582.00	Acute heart failure
Cong heart failure	G583.00	Heart failure with normal ejection fraction
Cong heart failure	G583.11	HFNEF - heart failure with normal ejection fraction
Cong heart failure	G583.12	Heart failure with preserved ejection fraction
Cong heart failure	G584.00	Right ventricular failure
Cong heart failure	G58z.00	Heart failure NOS
Cong heart failure	G58z.11	Weak heart
Cong heart failure	G58z.12	Cardiac failure NOS
Cong heart failure	G5yy900	Left ventricular systolic dysfunction
Cong heart failure	G5yyA00	Left ventricular diastolic dysfunction
Cong heart failure	G5yyB00	Right ventricular diastolic dysfunction
Cong heart failure	G5yyC00	Diastolic dysfunction
Cong heart failure	G5yyD00	Left ventricular cardiac dysfunction
Cong heart failure	R055100	[D]Cardiogenic shock
Cong heart failure	R055111	[D]Heart shock
Cong heart failure	R2y1000	[D]Cardiorespiratory failure
Connective tiss disord	14G1.00	H/O: rheumatoid arthritis
Connective tiss disord	43F1.00	Rheumatoid factor positive
Connective tiss disord	66H..00	Rheumatol. disorder monitoring
Connective tiss disord	66H..13	Rheumatoid arthrit. monitoring
Connective tiss disord	66H3.00	Rheumat.dis.- joints affected
Connective tiss disord	66H4.00	Rheumat. symptom change
Connective tiss disord	66H5.00	Rheumat. drug side effect
Connective tiss disord	66H6.00	Rheumat. treatment change
Connective tiss disord	66H6.11	Rheumat.dis.treatment changed
Connective tiss disord	66H7.00	Rheumat.dis.treatment started
Connective tiss disord	66H8.00	Rheumat.dis.treatment stopped
Connective tiss disord	66HB.00	Rheumatology disorder annual review
Connective tiss disord	66HB000	Rheumatoid arthritis annual review
Connective tiss disord	66HC.00	Rheumatic disorder annual review invitation
Connective tiss disord	66HZ.00	Rheumatol.dis. monitoring NOS
Connective tiss disord	AD5..00	Sarcoidosis
Connective tiss disord	AD50.00	Sarcoidosis of lung
Connective tiss disord	AD51.00	Sarcoidosis of lymph nodes
Connective tiss disord	AD52.00	Sarcoidosis of lung with sarcoidosis of lymph nodes
Connective tiss disord	AD53.00	Sarcoidosis of skin
Connective tiss disord	AD53000	Lupus pernio
Connective tiss disord	AD54.00	Sarcoidosis of inferior turbinates
Connective tiss disord	AD55.00	Sarcoid arthropathy
Connective tiss disord	C332100	Cryoglobulinaemic vasculitis
Connective tiss disord	Cyu0600	[X]Sarcoidosis of other and combined sites
Connective tiss disord	F013.00	Meningitis due to sarcoidosis
Connective tiss disord	F326300	Multiple cranial nerve palsies in sarcoidosis

Connective tiss disord	F371.00	Polyneuropathy in collagen vascular dis
Connective tiss disord	F371000	Polyneuropathy in disseminated lupus erythematosus
Connective tiss disord	F371100	Polyneuropathy in polyarteritis nodosa
Connective tiss disord	F371200	Polyneuropathy in rheumatoid arthritis
Connective tiss disord	F371z00	Polyneuropathy in collagen vascular dis NOS
Connective tiss disord	F374900	Polyneuropathy in sarcoidosis
Connective tiss disord	F396100	Myopathy due to disseminated lupus erythematosus
Connective tiss disord	F396300	Myopathy due to polyarteritis nodosa
Connective tiss disord	F396400	Myopathy due to rheumatoid arthritis
Connective tiss disord	F396500	Myopathy due to sarcoidosis
Connective tiss disord	F396600	Myopathy due to scleroderma
Connective tiss disord	F396700	Myopathy due to Sjogren's dis
Connective tiss disord	G558300	Sarcoid heart dis
Connective tiss disord	G5y7.00	Sarcoid myocarditis
Connective tiss disord	G5y8.00	Rheumatoid myocarditis
Connective tiss disord	G5yA.00	Rheumatoid carditis
Connective tiss disord	G75..00	Polyarteritis nodosa and allied conditions
Connective tiss disord	G750.00	Polyarteritis nodosa
Connective tiss disord	G750.11	Necrotising angiitis
Connective tiss disord	G751.00	Acute febrile mucocutaneous lymph node synde
Connective tiss disord	G751000	Kawasaki dis
Connective tiss disord	G751z00	Acute febrile mucocutaneous lymph node synde NOS
Connective tiss disord	G752.00	Hypersensitivity angiitis
Connective tiss disord	G752.11	Hypersensitivity arteritis
Connective tiss disord	G752000	Goodpasture's synde
Connective tiss disord	G752111	Antiglomerular basement membrane dis
Connective tiss disord	G752112	Anti GBM dis - Antiglomerular basement membrane dis
Connective tiss disord	G752z00	Hypersensitivity angiitis NOS
Connective tiss disord	G754.00	Wegener's granulomatosis
Connective tiss disord	G754.11	
Connective tiss disord	G755.00	Giant cell arteritis
Connective tiss disord	G755000	Cranial arteritis
Connective tiss disord	G755100	Temporal arteritis
Connective tiss disord	G755200	Horton's dis
Connective tiss disord	G755z00	Giant cell arteritis NOS
Connective tiss disord	G757.00	Takayasu's dis
Connective tiss disord	G757.11	Aortic arch arteritis
Connective tiss disord	G757.12	Pulseless dis
Connective tiss disord	G758.00	Churg-Strauss vasculitis
Connective tiss disord	G759.00	Juvenile polyarteritis
Connective tiss disord	G75A.00	Microscopic polyangiitis
Connective tiss disord	G75z.00	Polyarteritis nodosa and allied conditions NOS
Connective tiss disord	G766.00	Arteritis unspecified
Connective tiss disord	G766.11	Aortitis

Connective tiss disord	G76B.00	Vasculitis
Connective tiss disord	H570.00	Rheumatoid lung
Connective tiss disord	H571.00	Rheumatic pneumonia
Connective tiss disord	H572.00	Lung dis with systemic sclerosis
Connective tiss disord	H57y100	Lung dis with polymyositis
Connective tiss disord	H57y200	Pulmonary sarcoidosis
Connective tiss disord	H57y300	Lung dis with Sjogren's dis
Connective tiss disord	H57y400	Lung dis with systemic lupus erythematosus
Connective tiss disord	J63A.00	Hepatic granulomas in sarcoidosis
Connective tiss disord	K01x300	Nephrotic synde in polyarteritis nodosa
Connective tiss disord	K01x400	Nephrotic synde in systemic lupus erythematosus
Connective tiss disord	K01x411	Lupus nephritis
Connective tiss disord	K0H..00	Acute scleroderma renal crisis
Connective tiss disord	K0J0.00	Renal involvement in scleroderma
Connective tiss disord	N000.00	Systemic lupus erythematosus
Connective tiss disord	N000000	Disseminated lupus erythematosus
Connective tiss disord	N000100	Libman-Sacks dis
Connective tiss disord	N000200	Drug-induced systemic lupus erythematosus
Connective tiss disord	N000300	Systemic lupus erythematosus with organ or sys involv
Connective tiss disord	N000400	Systemic lupus erythematosus with pericarditis
Connective tiss disord	N000500	Neonatal lupus erythematosus
Connective tiss disord	N000600	Cerebral lupus
Connective tiss disord	N000z00	Systemic lupus erythematosus NOS
Connective tiss disord	N001.00	Scleroderma
Connective tiss disord	N001.11	Acrosclerosis
Connective tiss disord	N001.12	Systemic sclerosis
Connective tiss disord	N001000	Progressive systemic sclerosis
Connective tiss disord	N001100	CREST synde
Connective tiss disord	N002.00	Sicca (Sjogren's) synde
Connective tiss disord	N002.11	Keratoconjunctivitis sicca
Connective tiss disord	N004.00	Polymyositis
Connective tiss disord	N005.00	Adult Still's Dis
Connective tiss disord	N04..00	Rheumatoid arthritis other inflammatory polyarthropathy
Connective tiss disord	N040.00	Rheumatoid arthritis
Connective tiss disord	N040000	Rheumatoid arthritis of cervical spine
Connective tiss disord	N040100	Other rheumatoid arthritis of spine
Connective tiss disord	N040200	Rheumatoid arthritis of shoulder
Connective tiss disord	N040300	Rheumatoid arthritis of sternoclavicular joint
Connective tiss disord	N040400	Rheumatoid arthritis of acromioclavicular joint
Connective tiss disord	N040500	Rheumatoid arthritis of elbow
Connective tiss disord	N040600	Rheumatoid arthritis of distal radio-ulnar joint
Connective tiss disord	N040700	Rheumatoid arthritis of wrist
Connective tiss disord	N040800	Rheumatoid arthritis of MCP joint
Connective tiss disord	N040900	Rheumatoid arthritis of PIP joint of finger

Connective tiss disord	N040A00	Rheumatoid arthritis of DIP joint of finger
Connective tiss disord	N040B00	Rheumatoid arthritis of hip
Connective tiss disord	N040C00	Rheumatoid arthritis of sacro-iliac joint
Connective tiss disord	N040D00	Rheumatoid arthritis of knee
Connective tiss disord	N040E00	Rheumatoid arthritis of tibio-fibular joint
Connective tiss disord	N040F00	Rheumatoid arthritis of ankle
Connective tiss disord	N040G00	Rheumatoid arthritis of subtalar joint
Connective tiss disord	N040H00	Rheumatoid arthritis of talonavicular joint
Connective tiss disord	N040J00	Rheumatoid arthritis of other tarsal joint
Connective tiss disord	N040K00	Rheumatoid arthritis of 1st MTP joint
Connective tiss disord	N040L00	Rheumatoid arthritis of lesser MTP joint
Connective tiss disord	N040M00	Rheumatoid arthritis of IP joint of toe
Connective tiss disord	N040N00	Rheumatoid vasculitis
Connective tiss disord	N040P00	Seronegative rheumatoid arthritis
Connective tiss disord	N040Q00	Rheumatoid bursitis
Connective tiss disord	N040R00	Rheumatoid nodule
Connective tiss disord	N040S00	Rheumatoid arthritis - multiple joint
Connective tiss disord	N040T00	Flare of rheumatoid arthritis
Connective tiss disord	N041.00	Felty's synde
Connective tiss disord	N042.00	Other rheumatoid arthropathy+visceral/system involv
Connective tiss disord	N042100	Rheumatoid lung dis
Connective tiss disord	N042200	Rheumatoid nodule
Connective tiss disord	N042z00	Rheumatoid arthropathy + visceral/systemic involvement
Connective tiss disord	N043.00	Juvenile rheumatoid arthritis - Still's dis
Connective tiss disord	N043000	Juvenile rheumatoid arthropathy unspecified
Connective tiss disord	N043100	Acute polyarticular juvenile rheumatoid arthritis
Connective tiss disord	N043200	Pauciarticular juvenile rheumatoid arthritis
Connective tiss disord	N043300	Monarticular juvenile rheumatoid arthritis
Connective tiss disord	N043z00	Juvenile rheumatoid arthritis NOS
Connective tiss disord	N045500	Juvenile rheumatoid arthritis
Connective tiss disord	N047.00	Seropositive errosive rheumatoid arthritis
Connective tiss disord	N04X.00	Seropositive rheumatoid arthritis, unspecified
Connective tiss disord	N04y000	Rheumatoid lung
Connective tiss disord	N04y011	Caplan's synde
Connective tiss disord	N04y012	Fibrosing alveolitis associated with rheumatoid arthritis
Connective tiss disord	N04y200	Adult-onset Still's dis
Connective tiss disord	N20..00	Polymyalgia rheumatica
Connective tiss disord	N20..11	Polymyalgia
Connective tiss disord	N200.00	Giant cell arteritis with polymyalgia rheumatica
Connective tiss disord	N233200	Myositis in sarcoidosis
Connective tiss disord	Nyu1000	[X]Rheumatoid arthritis+involvement/other organs or syst
Connective tiss disord	Nyu1100	[X]Other seropositive rheumatoid arthritis
Connective tiss disord	Nyu1200	[X]Other specified rheumatoid arthritis
Connective tiss disord	Nyu1G00	[X]Seropositive rheumatoid arthritis, unspecified

Connective tiss disord	Nyu4.00	[X]Systemic Connective tiss disords
Connective tiss disord	Nyu4100	[X]Other giant cell arteritis
Connective tiss disord	Nyu4300	[X]Other forms of systemic lupus erythematosus
Connective tiss disord	Nyu4500	[X]Other forms of systemic sclerosis
Connective tiss disord	Nyu4600	[X]Other overlap syndes
Connective tiss disord	Nyu4700	[X]Other systemic diss of connective tissue
Connective tiss disord	Nyu4C00	[X]Systemic disorders/connective tissue in other diss CE
Connective tiss disord	Nyu4F00	[X]Mixed connective tissue dis
Dementia	1461	H/O: dementia
Dementia	66h..00	Dementia monitoring
Dementia	6AB..00	Dementia annual review
Dementia	9Ou..00	Dementia monitoring administration
Dementia	9Ou1.00	Dementia monitoring first letter
Dementia	9Ou2.00	Dementia monitoring second letter
Dementia	9Ou3.00	Dementia monitoring third letter
Dementia	9Ou4.00	Dementia monitoring verbal invite
Dementia	9Ou5.00	Dementia monitoring telephone invite
Dementia	A411.00	Jakob-Creutzfeldt dis
Dementia	E00..11	Senile dementia
Dementia	E00..12	Senile/presenile dementia
Dementia	E000.00	Uncomplicated senile dementia
Dementia	E001.00	Presenile dementia
Dementia	E001000	Uncomplicated presenile dementia
Dementia	E001100	Presenile dementia with delirium
Dementia	E001200	Presenile dementia with paranoia
Dementia	E001300	Presenile dementia with depression
Dementia	E001z00	Presenile dementia NOS
Dementia	E002.00	Senile dementia with depressive or paranoid features
Dementia	E002000	Senile dementia with paranoia
Dementia	E002100	Senile dementia with depression
Dementia	E002z00	Senile dementia with depressive or paranoid features
Dementia	E003.00	Senile dementia with delirium
Dementia	E004.00	Arteriosclerotic dementia
Dementia	E004.11	Multi infarct dementia
Dementia	E004000	Uncomplicated arteriosclerotic dementia
Dementia	E004100	Arteriosclerotic dementia with delirium
Dementia	E004200	Arteriosclerotic dementia with paranoia
Dementia	E004300	Arteriosclerotic dementia with depression
Dementia	E004z00	Arteriosclerotic dementia NOS
Dementia	E012.00	Other alcoholic dementia
Dementia	E012.11	Alcoholic dementia NOS
Dementia	E02y100	Drug-induced dementia
Dementia	E041.00	Dementia in conditions EC
Dementia	Eu00.00	[X]Dementia in Alzheimer's dis

Dementia	Eu00000	[X]Dementia in Alzheimer's dis with early onset
Dementia	Eu00011	[X]Presenile dementia,Alzheimer's type
Dementia	Eu00012	[X]Primary degen dement, Alzheim's typ, presenile onset
Dementia	Eu00013	[X]Alzheimer's dis type 2
Dementia	Eu00100	[X]Dementia in Alzheimer's dis with late onset
Dementia	Eu00111	[X]Alzheimer's dis type 1
Dementia	Eu00112	[X]Senile dementia,Alzheimer's type
Dementia	Eu00113	[X]Primary degen dementia of Alzheim's type, senile onset
Dementia	Eu00200	[X]Dementia in Alzheimer's dis, atypical or mixed type
Dementia	Eu00z00	[X]Dementia in Alzheimer's dis, unspecified
Dementia	Eu00z11	[X]Alzheimer's dementia unspec
Dementia	Eu01.00	[X]Vascular dementia
Dementia	Eu01.11	[X]Arteriosclerotic dementia
Dementia	Eu01000	[X]Vascular dementia of acute onset
Dementia	Eu01100	[X]Multi-infarct dementia
Dementia	Eu01111	[X]Predominantly cortical dementia
Dementia	Eu01200	[X]Subcortical vascular dementia
Dementia	Eu01300	[X]Mixed cortical and subcortical vascular dementia
Dementia	Eu01y00	[X]Other vascular dementia
Dementia	Eu01z00	[X]Vascular dementia, unspecified
Dementia	Eu02.00	[X]Dementia in other diss classified elsewhere
Dementia	Eu02000	[X]Dementia in Pick's dis
Dementia	Eu02100	[X]Dementia in Creutzfeldt-Jakob dis
Dementia	Eu02200	[X]Dementia in Huntington's dis
Dementia	Eu02300	[X]Dementia in Parkinson's dis
Dementia	Eu02400	[X]Dementia in human immunodef virus [HIV] dis
Dementia	Eu02500	[X]Lewy body dementia
Dementia	Eu02y00	[X]Dementia in other specified diss classif elsewhere
Dementia	Eu02z00	[X] Unspecified dementia
Dementia	Eu02z11	[X] Presenile dementia NOS
Dementia	Eu02z13	[X] Primary degenerative dementia NOS
Dementia	Eu02z14	[X] Senile dementia NOS
Dementia	Eu02z16	[X] Senile dementia, depressed or paranoid type
Dementia	Eu04100	[X]Delirium superimposed on dementia
Dementia	Eu10711	[X]Alcoholic dementia NOS
Dementia	Eu84311	[X]Dementia infantalis
Dementia	F110.00	Alzheimer's dis
Dementia	F110000	Alzheimer's dis with early onset
Dementia	F110100	Alzheimer's dis with late onset
Dementia	F111.00	Pick's dis
Dementia	F112.00	Senile degeneration of brain
Dementia	F116.00	Lewy body dis
Dementia	Fyu3000	[X]Other Alzheimer's dis
Dementia	ZS7C500	Language disorder of dementia

Diabetes	42W2.00	Hb. A1C 7-10% - borderline
Diabetes	42W3.00	Hb. A1C > 10% - bad control
Diabetes	66A4.00	Diabetic on oral treatment
Diabetes	66A5.00	Diabetic on insulin
Diabetes	66AJ.00	Diabetic - poor control
Diabetes	66AJ.11	Unstable diabetes
Diabetes	66AJ100	Brittle diabetes
Diabetes	66AJz00	Diabetic - poor control NOS
Diabetes	66AV.00	Diabetic on insulin and oral treatment
Diabetes	C10..00	Diabetes mellitus
Diabetes	C100.00	Diabetes mellitus with no mention of complication
Diabetes	C100000	Diabetes mellitus, juvenile type, no mention of complicat
Diabetes	C100011	Insulin dependent diabetes mellitus
Diabetes	C100100	Diabetes mellitus, adult onset, no mention of complicat
Diabetes	C100111	Maturity onset diabetes
Diabetes	C100112	Non-insulin dependent diabetes mellitus
Diabetes	C100z00	Diabetes mellitus NOS with no mention of complication
Diabetes	C101.00	Diabetes mellitus with ketoacidosis
Diabetes	C101000	Diabetes mellitus, juvenile type, with ketoacidosis
Diabetes	C101100	Diabetes mellitus, adult onset, with ketoacidosis
Diabetes	C101y00	Other specified diabetes mellitus with ketoacidosis
Diabetes	C101z00	Diabetes mellitus NOS with ketoacidosis
Diabetes	C102.00	Diabetes mellitus with hyperosmolar coma
Diabetes	C102000	Diabetes mellitus, juvenile type, with hyperosmolar coma
Diabetes	C102100	Diabetes mellitus, adult onset, with hyperosmolar coma
Diabetes	C102z00	Diabetes mellitus NOS with hyperosmolar coma
Diabetes	C103.00	Diabetes mellitus with ketoacidotic coma
Diabetes	C103000	Diabetes mellitus, juvenile type, with ketoacidotic coma
Diabetes	C103100	Diabetes mellitus, adult onset, with ketoacidotic coma
Diabetes	C103y00	Other specified diabetes mellitus with coma
Diabetes	C103z00	Diabetes mellitus NOS with ketoacidotic coma
Diabetes	C108.00	Insulin dependent diabetes mellitus
Diabetes	C108.11	IDDM-Insulin dependent diabetes mellitus
Diabetes	C108.12	Type 1 diabetes mellitus
Diabetes	C108.13	Type I diabetes mellitus
Diabetes	C108400	Unstable insulin dependent diabetes mellitus
Diabetes	C108411	Unstable type I diabetes mellitus
Diabetes	C108412	Unstable type 1 diabetes mellitus
Diabetes	C108800	Insulin dependent diabetes mellitus - poor control
Diabetes	C108811	Type I diabetes mellitus - poor control
Diabetes	C108812	Type 1 diabetes mellitus - poor control
Diabetes	C108900	Insulin dependent diabetes maturity onset
Diabetes	C108911	Type I diabetes mellitus maturity onset
Diabetes	C108912	Type 1 diabetes mellitus maturity onset

Diabetes	C108A00	Insulin-dependent diabetes without complication
Diabetes	C108A11	Type I diabetes mellitus without complication
Diabetes	C108E00	Insulin depend diabetes mell with hypoglycaemic coma
Diabetes	C108E11	Type I diabetes mellitus with hypoglycaemic coma
Diabetes	C108E12	Type 1 diabetes mellitus with hypoglycaemic coma
Diabetes	C109.00	Non-insulin dependent diabetes mellitus
Diabetes	C109.11	NIDDM - Non-insulin dependent diabetes mellitus
Diabetes	C109.12	Type 2 diabetes mellitus
Diabetes	C109.13	Type II diabetes mellitus
Diabetes	C109700	Non-insulin dependent diabetes mellitus - poor control
Diabetes	C109711	Type II diabetes mellitus - poor control
Diabetes	C109712	Type 2 diabetes mellitus - poor control
Diabetes	C109900	Non-insulin-dependent diabetes mell without complicat
Diabetes	C109911	Type II diabetes mellitus without complication
Diabetes	C109912	Type 2 diabetes mellitus without complication
Diabetes	C109D00	Noninsulin dependent diabetes mell with hypoglyca coma
Diabetes	C109D11	Type II diabetes mellitus with hypoglycaemic coma
Diabetes	C109D12	Type 2 diabetes mellitus with hypoglycaemic coma
Diabetes	C109J00	Insulin treated Type 2 diabetes mellitus
Diabetes	C109J11	Insulin treated non-insulin dependent diabetes mellitus
Diabetes	C109J12	Insulin treated Type II diabetes mellitus
Diabetes	C109K00	Hyperosmolar nonketotic state in type 2 diabetes mellitus
Diabetes	C10A.00	Malnutrition-related diabetes mellitus
Diabetes	C10A000	Malnutrition-related diabetes mellitus with coma
Diabetes	C10A100	Malnutrition-related diabetes mellitus with ketoacidosis
Diabetes	C10B.00	Diabetes mellitus induced by steroids
Diabetes	C10B000	Steroid induced diabetes mellitus without complication
Diabetes	C10C.00	Diabetes mellitus autosomal dominant
Diabetes	C10C.11	Maturity onset diabetes in youth
Diabetes	C10C.12	Maturity onset diabetes in youth type 1
Diabetes	C10D.00	Diabetes mellitus autosomal dominant type 2
Diabetes	C10D.11	Maturity onset diabetes in youth type 2
Diabetes	C10E.00	Type 1 diabetes mellitus
Diabetes	C10E.11	Type I diabetes mellitus
Diabetes	C10E.12	Insulin dependent diabetes mellitus
Diabetes	C10E400	Unstable type 1 diabetes mellitus
Diabetes	C10E411	Unstable type I diabetes mellitus
Diabetes	C10E412	Unstable insulin dependent diabetes mellitus
Diabetes	C10E800	Type 1 diabetes mellitus - poor control
Diabetes	C10E811	Type I diabetes mellitus - poor control
Diabetes	C10E812	Insulin dependent diabetes mellitus - poor control
Diabetes	C10E900	Type 1 diabetes mellitus maturity onset
Diabetes	C10E911	Type I diabetes mellitus maturity onset
Diabetes	C10E912	Insulin dependent diabetes maturity onset

Diabetes	C10EA00	Type 1 diabetes mellitus without complication
Diabetes	C10EA11	Type I diabetes mellitus without complication
Diabetes	C10EA12	Insulin-dependent diabetes without complication
Diabetes	C10EE00	Type 1 diabetes mellitus with hypoglycaemic coma
Diabetes	C10EE12	Insulin depend diabetes mell with hypoglycaemic coma
Diabetes	C10EM00	Type 1 diabetes mellitus with ketoacidosis
Diabetes	C10EM11	Type I diabetes mellitus with ketoacidosis
Diabetes	C10EN00	Type 1 diabetes mellitus with ketoacidotic coma
Diabetes	C10EN11	Type I diabetes mellitus with ketoacidotic coma
Diabetes	C10ER00	Latent autoimmune diabetes mellitus in adult
Diabetes	C10F.00	Type 2 diabetes mellitus
Diabetes	C10F.11	Type II diabetes mellitus
Diabetes	C10F700	Type 2 diabetes mellitus - poor control
Diabetes	C10F711	Type II diabetes mellitus - poor control
Diabetes	C10F900	Type 2 diabetes mellitus without complication
Diabetes	C10F911	Type II diabetes mellitus without complication
Diabetes	C10FD00	Type 2 diabetes mellitus with hypoglycaemic coma
Diabetes	C10FD11	Type II diabetes mellitus with hypoglycaemic coma
Diabetes	C10FJ00	Insulin treated Type 2 diabetes mellitus
Diabetes	C10FJ11	Insulin treated Type II diabetes mellitus
Diabetes	C10FK00	Hyperosmolar non-ketotic state in type 2 diabetes melli
Diabetes	C10FK11	Hyperosmolar non-ketotic state in type II diabetes melli
Diabetes	C10FN00	Type 2 diabetes mellitus with ketoacidosis
Diabetes	C10FN11	Type II diabetes mellitus with ketoacidosis
Diabetes	C10FP00	Type 2 diabetes mellitus with ketoacidotic coma
Diabetes	C10FP11	Type II diabetes mellitus with ketoacidotic coma
Diabetes	C10FS00	Maternally inherited diabetes mellitus
Diabetes	C10G.00	Secondary pancreatic diabetes mellitus
Diabetes	C10G000	Secondary pancreatic diabetes mellitus without complic
Diabetes	C10H.00	Diabetes mellitus induced by non-steroid drugs
Diabetes	C10H000	DM induced by non-steroid drugs without complication
Diabetes	C10M.00	Lipoatrophic diabetes mellitus
Diabetes	C10N.00	Secondary diabetes mellitus
Diabetes	C10N000	Secondary diabetes mellitus without complication
Diabetes	C10N100	Cystic fibrosis related diabetes mellitus
Diabetes	C10y.00	Diabetes mellitus with other specified manifestation
Diabetes	C10y100	Diabetes mellitus, adult, + other specified manifestation
Diabetes	C10yy00	Other specified diabetes mellitus with other spec comps
Diabetes	C10yz00	Diabetes mellitus NOS with other specified manifestation
Diabetes	C10z.00	Diabetes mellitus with unspecified complication
Diabetes	C10z000	Diabetes mellitus, juvenile type, + unspecified complic
Diabetes	C10z100	Diabetes mellitus, adult onset, + unspecified complication
Diabetes	C10zy00	Other specified diabetes mellitus with unspecified comps
Diabetes	C10zz00	Diabetes mellitus NOS with unspecified complication

Diabetes	C11y000	Steroid induced diabetes
Diabetes	C314.00	Renal glycosuria
Diabetes	C314.11	Renal diabetes
Diabetes	Cyu2.00	[X]Diabetes mellitus
Diabetes	Cyu2000	[X]Other specified diabetes mellitus
Diabetes	F420700	High risk proliferative diabetic retinopathy
Diabetes	F420800	High risk non proliferative diabetic retinopathy
Diabetes	L180500	Pre-existing diabetes mellitus, insulin-dependent
Diabetes	L180600	Pre-existing diabetes mellitus, non-insulin-dependent
Diabetes	L180700	Pre-existing malnutrition-related diabetes mellitus
Diabetes	L180X00	Pre-existing diabetes mellitus, unspecified
Diabetes	PKyP.00	Diab insipidus,diab mell,optic atrophy and deafness
Diabetes	PKyP.11	Wolfram synde
Diabetes with complic	2BBF.00	Retinal abnormality - diabetes related
Diabetes with complic	2BBk.00	O/E - right eye stable treated prolif diabetic retinopathy
Diabetes with complic	2BBl.00	O/E - left eye stable treated prolif diabetic retinopathy
Diabetes with complic	2BBL.00	O/E - diabetic maculopathy present both eyes
Diabetes with complic	2BBo.00	O/E - sight threatening diabetic retinopathy
Diabetes with complic	2BBP.00	O/E - right eye background diabetic retinopathy
Diabetes with complic	2BBQ.00	O/E - left eye background diabetic retinopathy
Diabetes with complic	2BBr.00	Impaired vision due to diabetic retinopathy
Diabetes with complic	2BBR.00	O/E - right eye preproliferative diabetic retinopathy
Diabetes with complic	2BBS.00	O/E - left eye preproliferative diabetic retinopathy
Diabetes with complic	2BBT.00	O/E - right eye proliferative diabetic retinopathy
Diabetes with complic	2BBV.00	O/E - left eye proliferative diabetic retinopathy
Diabetes with complic	2BBW.00	O/E - right eye diabetic maculopathy
Diabetes with complic	2BBX.00	O/E - left eye diabetic maculopathy
Diabetes with complic	2G51000	Foot abnormality - diabetes related
Diabetes with complic	2G5C.00	Foot abnormality - diabetes related
Diabetes with complic	2G5H.00	O/E - Right diabetic foot - ulcerated
Diabetes with complic	2G5L.00	O/E - Left diabetic foot - ulcerated
Diabetes with complic	2G5V.00	O/E - right chronic diabetic foot ulcer
Diabetes with complic	2G5W.00	O/E - left chronic diabetic foot ulcer
Diabetes with complic	C104.00	Diabetes mellitus with renal manifestation
Diabetes with complic	C104.11	Diabetic nephropathy
Diabetes with complic	C104000	Diabetes mellitus, juvenile type, with renal manifestation
Diabetes with complic	C104100	Diabetes mellitus, adult onset, with renal manifestation
Diabetes with complic	C104y00	Other specified diabetes mellitus with renal complic
Diabetes with complic	C104z00	Diabetes mellitus with nephropathy NOS
Diabetes with complic	C105.00	Diabetes mellitus with ophthalmic manifestation
Diabetes with complic	C105000	Diabetes mell, juvenile type+ophthalmic manifestation
Diabetes with complic	C105100	Diabetes mellitus, adult onset+ophthalmic manifestation
Diabetes with complic	C105y00	Other specified diabetes mellitus with ophthalmic complic
Diabetes with complic	C105z00	Diabetes mellitus NOS with ophthalmic manifestation

Diabetes with complic	C106.00	Diabetes mellitus with neurological manifestation
Diabetes with complic	C106.11	Diabetic amyotrophy
Diabetes with complic	C106.12	Diabetes mellitus with neuropathy
Diabetes with complic	C106.13	Diabetes mellitus with polyneuropathy
Diabetes with complic	C106000	Diabetes mellitus, juvenile, + neurological manifestation
Diabetes with complic	C106100	Diabetes mellitus, adult onset+neurological manifestation
Diabetes with complic	C106y00	Other specified diabetes mellitus with neurological comps
Diabetes with complic	C106z00	Diabetes mellitus NOS with neurological manifestation
Diabetes with complic	C107.00	Diabetes mellitus with peripheral circulatory disorder
Diabetes with complic	C107.11	Diabetes mellitus with gangrene
Diabetes with complic	C107.12	Diabetes with gangrene
Diabetes with complic	C107000	Diabetes mell, juvenile +peripheral circulatory disorder
Diabetes with complic	C107100	Diabetes mellitus, adult, + peripheral circulatory disorder
Diabetes with complic	C107200	Diabetes mellitus, adult with gangrene
Diabetes with complic	C107300	IDDM with peripheral circulatory disorder
Diabetes with complic	C107400	NIDDM with peripheral circulatory disorder
Diabetes with complic	C107z00	Diabetes mellitus NOS with peripheral circulatory disorder
Diabetes with complic	C108000	Insulin-dependent diabetes mellitus with renal complic
Diabetes with complic	C108011	Type I diabetes mellitus with renal complic
Diabetes with complic	C108012	Type 1 diabetes mellitus with renal complic
Diabetes with complic	C108100	Insulin-dependent diabetes mell with ophthalmic comps
Diabetes with complic	C108112	Type 1 diabetes mellitus with ophthalmic complic
Diabetes with complic	C108200	Insulin-dependent diabetes mell with neurological comps
Diabetes with complic	C108211	Type I diabetes mellitus with neurological complic
Diabetes with complic	C108212	Type 1 diabetes mellitus with neurological complic
Diabetes with complic	C108300	Insulin dependent diabetes mellitus with multiple compl
Diabetes with complic	C108311	Type I diabetes mellitus with multiple complic
Diabetes with complic	C108500	Insulin dependent diabetes mellitus with ulcer
Diabetes with complic	C108511	Type I diabetes mellitus with ulcer
Diabetes with complic	C108512	Type 1 diabetes mellitus with ulcer
Diabetes with complic	C108600	Insulin dependent diabetes mellitus with gangrene
Diabetes with complic	C108700	Insulin dependent diabetes mellitus with retinopathy
Diabetes with complic	C108711	Type I diabetes mellitus with retinopathy
Diabetes with complic	C108712	Type 1 diabetes mellitus with retinopathy
Diabetes with complic	C108B00	Insulin dependent diabetes mell with mononeuropathy
Diabetes with complic	C108B11	Type I diabetes mellitus with mononeuropathy
Diabetes with complic	C108C00	Insulin dependent diabetes mellitus with polyneuropathy
Diabetes with complic	C108D00	Insulin dependent diabetes mellitus with nephropathy
Diabetes with complic	C108D11	Type I diabetes mellitus with nephropathy
Diabetes with complic	C108F00	Insulin dependent diabetes mellitus with diabetic cataract
Diabetes with complic	C108F11	Type I diabetes mellitus with diabetic cataract
Diabetes with complic	C108G00	Insulin dependent diab mell with peripheral angiopathy
Diabetes with complic	C108H00	Insulin dependent diabetes mellitus with arthropathy
Diabetes with complic	C108H11	Type I diabetes mellitus with arthropathy

Diabetes with complic	C108J00	Insulin dependent diab mell with neuropathic arthropathy
Diabetes with complic	C108J11	Type I diabetes mellitus with neuropathic arthropathy
Diabetes with complic	C108J12	Type 1 diabetes mellitus with neuropathic arthropathy
Diabetes with complic	C108y00	Other specified diabetes mellitus with multiple comps
Diabetes with complic	C108z00	Unspecified diabetes mellitus with multiple complic
Diabetes with complic	C109000	Noninsulin-dependent diabetes mellitus with renal comps
Diabetes with complic	C109011	Type II diabetes mellitus with renal complic
Diabetes with complic	C109012	Type 2 diabetes mellitus with renal complic
Diabetes with complic	C109100	Noninsulin-dependent diabetes mell with ophthalm comp
Diabetes with complic	C109111	Type II diabetes mellitus with ophthalmic complic
Diabetes with complic	C109112	Type 2 diabetes mellitus with ophthalmic complic
Diabetes with complic	C109200	Noninsulin-dependent diabetes mell with neuro comps
Diabetes with complic	C109211	Type II diabetes mellitus with neurological complic
Diabetes with complic	C109212	Type 2 diabetes mellitus with neurological complic
Diabetes with complic	C109300	Noninsulin-dependent diabetes mell with multiple comps
Diabetes with complic	C109312	Type 2 diabetes mellitus with multiple complic
Diabetes with complic	C109400	Non-insulin dependent diabetes mellitus with ulcer
Diabetes with complic	C109411	Type II diabetes mellitus with ulcer
Diabetes with complic	C109412	Type 2 diabetes mellitus with ulcer
Diabetes with complic	C109500	Non-insulin dependent diabetes mellitus with gangrene
Diabetes with complic	C109511	Type II diabetes mellitus with gangrene
Diabetes with complic	C109512	Type 2 diabetes mellitus with gangrene
Diabetes with complic	C109600	Non-insulin-dependent diabetes mellitus with retinopathy
Diabetes with complic	C109611	Type II diabetes mellitus with retinopathy
Diabetes with complic	C109612	Type 2 diabetes mellitus with retinopathy
Diabetes with complic	C109A00	Noninsulin dependt diabetes mell with mononeuropathy
Diabetes with complic	C109A11	Type II diabetes mellitus with mononeuropathy
Diabetes with complic	C109B00	Noninsulin dependent diabetes mell with polyneuropathy
Diabetes with complic	C109B11	Type II diabetes mellitus with polyneuropathy
Diabetes with complic	C109C00	Non-insulin dependent diabetes mell with nephropathy
Diabetes with complic	C109C11	Type II diabetes mellitus with nephropathy
Diabetes with complic	C109C12	Type 2 diabetes mellitus with nephropathy
Diabetes with complic	C109E00	Non-insulin depend diabetes mell with diabetic cataract
Diabetes with complic	C109E11	Type II diabetes mellitus with diabetic cataract
Diabetes with complic	C109E12	Type 2 diabetes mellitus with diabetic cataract
Diabetes with complic	C109F00	Non-insulin-dependent d m with peripheral angiopath
Diabetes with complic	C109F11	Type II diabetes mellitus with peripheral angiopathy
Diabetes with complic	C109F12	Type 2 diabetes mellitus with peripheral angiopathy
Diabetes with complic	C109G00	Non-insulin dependent diabetes mellitus with arthropathy
Diabetes with complic	C109G11	Type II diabetes mellitus with arthropathy
Diabetes with complic	C109G12	Type 2 diabetes mellitus with arthropathy
Diabetes with complic	C109H00	Non-insulin dependent d m with neuropathic arthropathy
Diabetes with complic	C109H11	Type II diabetes mellitus with neuropathic arthropathy
Diabetes with complic	C109H12	Type 2 diabetes mellitus with neuropathic arthropathy

Diabetes with complic	C10A500	Malnutritn-relat diabetes mell wth periph circul complctn
Diabetes with complic	C10E000	Type 1 diabetes mellitus with renal complic
Diabetes with complic	C10E012	Insulin-dependent diabetes mellitus with renal complic
Diabetes with complic	C10E100	Type 1 diabetes mellitus with ophthalmic complic
Diabetes with complic	C10E111	Type I diabetes mellitus with ophthalmic complic
Diabetes with complic	C10E112	Insulindependent diabetes mell with ophthalmic comps
Diabetes with complic	C10E200	Type 1 diabetes mellitus with neurological complic
Diabetes with complic	C10E212	Insulindependent diabetes mell with neurological comps
Diabetes with complic	C10E300	Type 1 diabetes mellitus with multiple complic
Diabetes with complic	C10E311	Type I diabetes mellitus with multiple complic
Diabetes with complic	C10E312	Insulin dependent diabetes mell with multiple complicat
Diabetes with complic	C10E500	Type 1 diabetes mellitus with ulcer
Diabetes with complic	C10E511	Type I diabetes mellitus with ulcer
Diabetes with complic	C10E512	Insulin dependent diabetes mellitus with ulcer
Diabetes with complic	C10E600	Type 1 diabetes mellitus with gangrene
Diabetes with complic	C10E611	Type I diabetes mellitus with gangrene
Diabetes with complic	C10E612	Insulin dependent diabetes mellitus with gangrene
Diabetes with complic	C10E700	Type 1 diabetes mellitus with retinopathy
Diabetes with complic	C10E711	Type I diabetes mellitus with retinopathy
Diabetes with complic	C10E712	Insulin dependent diabetes mellitus with retinopathy
Diabetes with complic	C10EB00	Type 1 diabetes mellitus with mononeuropathy
Diabetes with complic	C10EC00	Type 1 diabetes mellitus with polyneuropathy
Diabetes with complic	C10EC11	Type I diabetes mellitus with polyneuropathy
Diabetes with complic	C10EC12	Insulin dependent diabetes mellitus with polyneuropathy
Diabetes with complic	C10ED00	Type 1 diabetes mellitus with nephropathy
Diabetes with complic	C10ED12	Insulin dependent diabetes mellitus with nephropathy
Diabetes with complic	C10EF00	Type 1 diabetes mellitus with diabetic cataract
Diabetes with complic	C10EF12	Insulin dependent diabetes mellitus with diabetic cataract
Diabetes with complic	C10EG00	Type 1 diabetes mellitus with peripheral angiopathy
Diabetes with complic	C10EH00	Type 1 diabetes mellitus with arthropathy
Diabetes with complic	C10EJ00	Type 1 diabetes mellitus with neuropathic arthropathy
Diabetes with complic	C10EK00	Type 1 diabetes mellitus with persistent proteinuria
Diabetes with complic	C10EL00	Type 1 diabetes mellitus with persistent microalbuminuria
Diabetes with complic	C10EL11	Type I diabetes mellitus with persistent microalbuminuria
Diabetes with complic	C10EP00	Type 1 diabetes mellitus with exudative maculopathy
Diabetes with complic	C10EP11	Type I diabetes mellitus with exudative maculopathy
Diabetes with complic	C10EQ00	Type 1 diabetes mellitus with gastroparesis
Diabetes with complic	C10EQ11	Type I diabetes mellitus with gastroparesis
Diabetes with complic	C10F000	Type 2 diabetes mellitus with renal complic
Diabetes with complic	C10F011	Type II diabetes mellitus with renal complic
Diabetes with complic	C10F100	Type 2 diabetes mellitus with ophthalmic complic
Diabetes with complic	C10F111	Type II diabetes mellitus with ophthalmic complic
Diabetes with complic	C10F200	Type 2 diabetes mellitus with neurological complic
Diabetes with complic	C10F211	Type II diabetes mellitus with neurological complic

Diabetes with complic	C10F300	Type 2 diabetes mellitus with multiple complic
Diabetes with complic	C10F311	Type II diabetes mellitus with multiple complic
Diabetes with complic	C10F400	Type 2 diabetes mellitus with ulcer
Diabetes with complic	C10F411	Type II diabetes mellitus with ulcer
Diabetes with complic	C10F500	Type 2 diabetes mellitus with gangrene
Diabetes with complic	C10F511	Type II diabetes mellitus with gangrene
Diabetes with complic	C10F600	Type 2 diabetes mellitus with retinopathy
Diabetes with complic	C10F611	Type II diabetes mellitus with retinopathy
Diabetes with complic	C10FA00	Type 2 diabetes mellitus with mononeuropathy
Diabetes with complic	C10FA11	Type II diabetes mellitus with mononeuropathy
Diabetes with complic	C10FB00	Type 2 diabetes mellitus with polyneuropathy
Diabetes with complic	C10FB11	Type II diabetes mellitus with polyneuropathy
Diabetes with complic	C10FC00	Type 2 diabetes mellitus with nephropathy
Diabetes with complic	C10FC11	Type II diabetes mellitus with nephropathy
Diabetes with complic	C10FE00	Type 2 diabetes mellitus with diabetic cataract
Diabetes with complic	C10FE11	Type II diabetes mellitus with diabetic cataract
Diabetes with complic	C10FF00	Type 2 diabetes mellitus with peripheral angiopathy
Diabetes with complic	C10FF11	Type II diabetes mellitus with peripheral angiopathy
Diabetes with complic	C10FG00	Type 2 diabetes mellitus with arthropathy
Diabetes with complic	C10FG11	Type II diabetes mellitus with arthropathy
Diabetes with complic	C10FH00	Type 2 diabetes mellitus with neuropathic arthropathy
Diabetes with complic	C10FH11	Type II diabetes mellitus with neuropathic arthropathy
Diabetes with complic	C10FL00	Type 2 diabetes mellitus with persistent proteinuria
Diabetes with complic	C10FL11	Type II diabetes mellitus with persistent proteinuria
Diabetes with complic	C10FM00	Type 2 diabetes mellitus with persistent microalbuminuria
Diabetes with complic	C10FM11	Type II diabetes mellitus with persistent microalbuminuria
Diabetes with complic	C10FQ00	Type 2 diabetes mellitus with exudative maculopathy
Diabetes with complic	C10FR00	Type 2 diabetes mellitus with gastroparesis
Diabetes with complic	Cyu2300	[X]Unspecified diabetes mellitus with renal complic
Diabetes with complic	F171100	Autonomic neuropathy due to diabetes
Diabetes with complic	F345000	Diabetic mononeuritis multiplex
Diabetes with complic	F35z000	Diabetic mononeuritis NOS
Diabetes with complic	F372.00	Polyneuropathy in diabetes
Diabetes with complic	F372.11	Diabetic polyneuropathy
Diabetes with complic	F372.12	Diabetic neuropathy
Diabetes with complic	F372000	Acute painful diabetic neuropathy
Diabetes with complic	F372100	Chronic painful diabetic neuropathy
Diabetes with complic	F372200	Asymptomatic diabetic neuropathy
Diabetes with complic	F381300	Myasthenic synde due to diabetic amyotrophy
Diabetes with complic	F381311	Diabetic amyotrophy
Diabetes with complic	F3y0.00	Diabetic mononeuropathy
Diabetes with complic	F420.00	Diabetic retinopathy
Diabetes with complic	F420000	Background diabetic retinopathy
Diabetes with complic	F420100	Proliferative diabetic retinopathy

Diabetes with complic	F420200	Preproliferative diabetic retinopathy
Diabetes with complic	F420300	Advanced diabetic maculopathy
Diabetes with complic	F420400	Diabetic maculopathy
Diabetes with complic	F420500	Advanced diabetic retinal dis
Diabetes with complic	F420600	Non proliferative diabetic retinopathy
Diabetes with complic	F420z00	Diabetic retinopathy NOS
Diabetes with complic	F440700	Diabetic iritis
Diabetes with complic	F464000	Diabetic cataract
Diabetes with complic	G73y000	Diabetic peripheral angiopathy
Diabetes with complic	K01x100	Nephrotic synde in diabetes mellitus
Diabetes with complic	K01x111	Kimmelstiel - Wilson dis
Diabetes with complic	K08yA00	Proteinuric diabetic nephropathy
Diabetes with complic	K08yA11	Clinical diabetic nephropathy
Diabetes with complic	Kyu0300	[X]Glomerular disorders in diabetes mellitus
Diabetes with complic	M037200	Cellulitis in diabetic foot
Diabetes with complic	M271000	Ischaemic ulcer diabetic foot
Diabetes with complic	M271100	Neuropathic diabetic ulcer - foot
Diabetes with complic	M271200	Mixed diabetic ulcer - foot
Diabetes with complic	N030000	Diabetic cheiroarthropathy
Diabetes with complic	N030011	Diabetic cheiropathy
Diabetes with complic	N030100	Diabetic Charcot arthropathy
Diabetes with complic	R054200	[D]Gangrene of toe in diabetic
Diabetes with complic	R054300	[D]Widespread diabetic foot gangrene
hemiplegia/paraplegia	2833	O/E - hemiplegia
hemiplegia/paraplegia	2835	O/E - paraplegia
hemiplegia/paraplegia	2836	O/E - quadriplegia
hemiplegia/paraplegia	F22..00	Hemiplegia
hemiplegia/paraplegia	F22..11	Hemiparesis
hemiplegia/paraplegia	F220.00	Flaccid hemiplegia
hemiplegia/paraplegia	F221.00	Spastic hemiplegia
hemiplegia/paraplegia	F221.11	Spastic foot
hemiplegia/paraplegia	F222.00	Left hemiplegia
hemiplegia/paraplegia	F222.11	Left sided weakness
hemiplegia/paraplegia	F223.00	Right hemiplegia
hemiplegia/paraplegia	F223.11	Right sided weakness
hemiplegia/paraplegia	F22z.00	Hemiplegia NOS
hemiplegia/paraplegia	F230.11	Paraplegia - congenital
hemiplegia/paraplegia	F230000	Congenital paraplegia
hemiplegia/paraplegia	F231.00	Congenital hemiplegia
hemiplegia/paraplegia	F232.00	Congenital quadriplegia
hemiplegia/paraplegia	F232.11	Tetraplegia - congenital
hemiplegia/paraplegia	F234.00	Infantile hemiplegia NOS
hemiplegia/paraplegia	F240.00	Quadriplegia
hemiplegia/paraplegia	F240.11	Tetraplegia

hemiplegia/paraplegia	F240000	Flaccid tetraplegia
hemiplegia/paraplegia	F240100	Spastic tetraplegia
hemiplegia/paraplegia	F241.00	Paraplegia
hemiplegia/paraplegia	F241000	Flaccid paraplegia
hemiplegia/paraplegia	F241100	Spastic paraplegia
hemiplegia/paraplegia	F2A..00	Hemiparesis
hemiplegia/paraplegia	F2Az.00	Hemiparesis NOS
hemiplegia/paraplegia	F2B0.00	Spastic quadriplegic cerebral palsy
hemiplegia/paraplegia	F2B1.00	Spastic hemiplegic cerebral palsy
HIV/AIDS	43C3.00	HTLV-3 antibody positive
HIV/AIDS	43C3.11	HIV positive
HIV/AIDS	A788.00	Acquired immune deficiency synde
HIV/AIDS	A788.11	Human immunodeficiency virus infection
HIV/AIDS	A788000	Acute human immunodeficiency virus infection
HIV/AIDS	A788100	Asymptomatic human immunodeficiency virus infection
HIV/AIDS	A788200	HIV inf with persistent generalised lymphadenopathy
HIV/AIDS	A788300	Human immunodeficiency virus with constitutional dis
HIV/AIDS	A788400	Human immunodeficiency virus with neurological dis
HIV/AIDS	A788500	Human immunodeficiency virus with secondary infection
HIV/AIDS	A788600	Human immunodeficiency virus with secondary cancers
HIV/AIDS	A788U00	HIV dis result/haematological+immunologic abnorms,NEC
HIV/AIDS	A788W00	HIV dis resulting in unspecified malignant neoplasm
HIV/AIDS	A788X00	HIV dis resulting/unspcf infectious+parasitic dis
HIV/AIDS	A788y00	Human immunodefic virus with other clinical findings
HIV/AIDS	A788z00	Acquired human immunodeficiency virus infection synde
HIV/AIDS	A789.00	Human immunodef virus resulting in other dis
HIV/AIDS	A789000	HIV dis resulting in mycobacterial infection
HIV/AIDS	A789100	HIV dis resulting in cytomegaloviral dis
HIV/AIDS	A789200	HIV dis resulting in candidiasis
HIV/AIDS	A789300	HIV dis resulting in Pneumocystis carinii pneumonia
HIV/AIDS	A789311	HIV dis resulting in Pneumocystis jirovecii pneumonia
HIV/AIDS	A789400	HIV dis resulting in multiple infections
HIV/AIDS	A789500	HIV dis resulting in Kaposi's sarcoma
HIV/AIDS	A789511	HIV dis resulting in Kaposi sarcoma
HIV/AIDS	A789600	HIV dis resulting in Burkitt's lymphoma
HIV/AIDS	A789700	HIV dis resulting oth types of non-Hodgkin's lymphoma
HIV/AIDS	A789800	HIV dis resulting in multiple malignant neoplasms
HIV/AIDS	A789900	HIV dis resulting in lymphoid interstitial pneumonitis
HIV/AIDS	A789A00	HIV dis resulting in wasting synde
HIV/AIDS	A789X00	HIV reslt/oth mal neopl/lymph,h'matopoetc+reltd tissu
HIV/AIDS	AyuC.00	[X]Human immunodeficiency virus dis
HIV/AIDS	AyuC100	[X]HIV dis resulting in other viral infections
HIV/AIDS	AyuC300	[X]HIV dis resulting in multiple infections
HIV/AIDS	AyuC400	[X]HIV dis resulting/other infectious+parasitic diss

HIV/AIDS	AyuC600	[X]HIV dis resulting in other non-Hodgkin's lymphoma
HIV/AIDS	AyuCB00	[X]HIV result/haematological+immunologic abnorms,NEC
HIV/AIDS	AyuCC00	[X]HIV dis resulting in other specified conditions
HIV/AIDS	AyuCD00	[X]Unspecified human immunodeficiency virus [HIV] dis
HIV/AIDS	Eu02400	[X]Dementia in human immunodef virus [HIV] dis
HIV/AIDS	L179.00	HIV dis complicating pregnancy childbirth puerperium
HIV/AIDS	R109.00	[D]Laboratory evidence of human immunodeficiency virus
HIV/AIDS	ZV01A00	[V]Asympto human immunodeficency virus infect status
Metastatic cancer	A788600	Human immunodeficiency virus with secondary cancers
Metastatic cancer	B153.00	Secondary malignant neoplasm of liver
Metastatic cancer	B56..00	Secondary unspecified malignant neoplasm lymph nodes
Metastatic cancer	B56..11	Lymph node metastases
Metastatic cancer	B560.00	Second unspec malig neop lymph nodes head/face/neck
Metastatic cancer	B560000	Secondary unspec malig neop of superficial parotid LN
Metastatic cancer	B560100	Secondary and unspec malignant neoplasm mastoid LN
Metastatic cancer	B560200	Secondary and unspec malig neop superficial cervical LN
Metastatic cancer	B560300	Secondary and unspec malignant neoplasm occipital LN
Metastatic cancer	B560400	Secondary and unspec malig neop deep parotid LN
Metastatic cancer	B560500	Secondary and unspec malig neop submandibular LN
Metastatic cancer	B560600	Secondary and unspec malig neop of facial lymph nodes
Metastatic cancer	B560700	Secondary and unspec malig neop submental LN
Metastatic cancer	B560800	Secondary and unspec malig neop anterior cervical LN
Metastatic cancer	B560900	Secondary and unspec malig neop deep cervical LN
Metastatic cancer	B560z00	Secondary unspec malig neop LN head/face/neck NOS
Metastatic cancer	B561.00	Secondary and unspec malig neop intrathoracic LN
Metastatic cancer	B561000	Secondary and unspec malig neop internal mammary LN
Metastatic cancer	B561100	Secondary and unspec malig neop intercostal LN
Metastatic cancer	B561200	Secondary and unspec malig neop diaphragmatic LN
Metastatic cancer	B561300	Secondary and unspec malig neop ant mediastinal LN
Metastatic cancer	B561400	Secondary and unspec malig neop post mediastinal LN
Metastatic cancer	B561500	Secondary and unspec malig neop paratracheal LN
Metastatic cancer	B561600	Second unspec malig neop superfic tracheobronchial LN
Metastatic cancer	B561700	Second unspec malig neop inferior tracheobronchial LN
Metastatic cancer	B561800	Second unspec malig neop bronchopulmonary LN
Metastatic cancer	B561900	Secondary and unspec malig neop pulmonary LN
Metastatic cancer	B561z00	Secondary and unspec malig neop intrathoracic LN NOS
Metastatic cancer	B562.00	Secondary and unspec malig neop intra-abdominal LN
Metastatic cancer	B562000	Secondary and unspec malig neop coeliac lymph nodes
Metastatic cancer	B562100	Secondary unspec malig neop superficial mesenteric LN
Metastatic cancer	B562200	Secondary and unspec malig neop inferior mesenteric LN
Metastatic cancer	B562300	Secondary and unspec malig neop common iliac LN
Metastatic cancer	B562400	Secondary and unspec malig neop external iliac LN
Metastatic cancer	B562z00	Secondary and unspec malig neop intra-abdominal LN
Metastatic cancer	B563.00	Secondary unspec malig neop axilla and upper limb LN

Metastatic cancer	B563000	Secondary and unspec malig neop axillary lymph nodes
Metastatic cancer	B563100	Secondary and unspec malig neop supratrochlear LN
Metastatic cancer	B563200	Secondary and unspec malig neop infraclavicular LN
Metastatic cancer	B563300	Secondary and unspec malig neop pectoral lymph nodes
Metastatic cancer	B563z00	Second unspec malig neop axilla and upper limb LN NOS
Metastatic cancer	B564.00	Second unspec malig neop inguinal and lower limb LN
Metastatic cancer	B564000	Secondary and unspec malig neop superficial inguinal LN
Metastatic cancer	B564100	Secondary and unspec malig neop deep inguinal LN
Metastatic cancer	B564z00	Secondary and unspec malig neop of inguinal and leg LN
Metastatic cancer	B565.00	Secondary and unspec malig neop intrapelvic LN
Metastatic cancer	B565000	Secondary and unspec malig neop internal iliac LN
Metastatic cancer	B565200	Secondary and unspec malig neop circumflex iliac LN
Metastatic cancer	B565300	Secondary and unspec malig neop sacral lymph nodes
Metastatic cancer	B565z00	Secondary and unspec malig neop intrapelvic LN NOS
Metastatic cancer	B56y.00	Secondary unspec malig neop lymph nodes multiple sites
Metastatic cancer	B56z.00	Secondary and unspec malig neop lymph nodes NOS
Metastatic cancer	B57..00	Secondary malig neop of respiratory and digestive syst
Metastatic cancer	B57..11	Metastases of respiratory and/or digestive systems
Metastatic cancer	B57..12	Secondary carcinoma of respiratory and/or digestive syst
Metastatic cancer	B570.00	Secondary malignant neoplasm of lung
Metastatic cancer	B571.00	Secondary malignant neoplasm of mediastinum
Metastatic cancer	B572.00	Secondary malignant neoplasm of pleura
Metastatic cancer	B573.00	Secondary malignant neoplasm of other respira organs
Metastatic cancer	B574.00	Second malignant neoplasm of small intestine duodenum
Metastatic cancer	B574000	Secondary malignant neoplasm of duodenum
Metastatic cancer	B574200	Secondary malignant neoplasm of ileum
Metastatic cancer	B574z00	Secondary malig neop of small intestine or duodenum
Metastatic cancer	B575.00	Secondary malignant neoplasm of large intestine rectum
Metastatic cancer	B575000	Secondary malignant neoplasm of colon
Metastatic cancer	B575100	Secondary malignant neoplasm of rectum
Metastatic cancer	B575z00	Secondary malig neop of large intestine or rectum NOS
Metastatic cancer	B576.00	Secondary malig neop of retroperitoneum peritoneum
Metastatic cancer	B576000	Secondary malignant neoplasm of retroperitoneum
Metastatic cancer	B576100	Secondary malignant neoplasm of peritoneum
Metastatic cancer	B576200	Malignant ascites
Metastatic cancer	B576z00	Secondary malig neop of retroperitoneum or peritoneum
Metastatic cancer	B577.00	Secondary malignant neoplasm of liver
Metastatic cancer	B577.11	Liver metastases
Metastatic cancer	B57y.00	Secondary malignant neoplasm of other digestive organ
Metastatic cancer	B57z.00	Secondary malig neop of respiratory or digestive system
Metastatic cancer	B58..00	Secondary malignant neoplasm of other specified sites
Metastatic cancer	B58..11	Secondary carcinoma of other specified sites
Metastatic cancer	B580.00	Secondary malignant neoplasm of kidney
Metastatic cancer	B581.00	Secondary malignant neoplasm of other urinary organs

Metastatic cancer	B581000	Secondary malignant neoplasm of ureter
Metastatic cancer	B581100	Secondary malignant neoplasm of bladder
Metastatic cancer	B581200	Secondary malignant neoplasm of urethra
Metastatic cancer	B581z00	Secondary malignant neoplasm of other urinary organ
Metastatic cancer	B582.00	Secondary malignant neoplasm of skin
Metastatic cancer	B582000	Secondary malignant neoplasm of skin of head
Metastatic cancer	B582100	Secondary malignant neoplasm of skin of face
Metastatic cancer	B582200	Secondary malignant neoplasm of skin of neck
Metastatic cancer	B582300	Secondary malignant neoplasm of skin of trunk
Metastatic cancer	B582400	Secondary malignant neoplasm of skin of shoulder/arm
Metastatic cancer	B582500	Secondary malignant neoplasm of skin of hip and leg
Metastatic cancer	B582600	Secondary malignant neoplasm of skin of breast
Metastatic cancer	B582z00	Secondary malignant neoplasm of skin NOS
Metastatic cancer	B583.00	Secondary malignant neoplasm of brain and spinal cord
Metastatic cancer	B583000	Secondary malignant neoplasm of brain
Metastatic cancer	B583100	Secondary malignant neoplasm of spinal cord
Metastatic cancer	B583200	Cerebral metastasis
Metastatic cancer	B583z00	Secondary malignant neoplasm of brain or spinal cord
Metastatic cancer	B584.00	Secondary malignant neoplasm of other part nervous syst
Metastatic cancer	B585.00	Secondary malignant neoplasm of bone and bone marrow
Metastatic cancer	B585000	Pathological fracture due to metastatic bone dis
Metastatic cancer	B586.00	Secondary malignant neoplasm of ovary
Metastatic cancer	B587.00	Secondary malignant neoplasm of adrenal gland
Metastatic cancer	B58y.00	Secondary malignant neoplasm of other specified sites
Metastatic cancer	B58y000	Secondary malignant neoplasm of breast
Metastatic cancer	B58y100	Secondary malignant neoplasm of uterus
Metastatic cancer	B58y200	Secondary malignant neoplasm of cervix uteri
Metastatic cancer	B58y211	Secondary cancer of the cervix
Metastatic cancer	B58y300	Secondary malignant neoplasm of vagina
Metastatic cancer	B58y400	Secondary malignant neoplasm of vulva
Metastatic cancer	B58y411	Secondary cancer of the vulva
Metastatic cancer	B58y500	Secondary malignant neoplasm of prostate
Metastatic cancer	B58y600	Secondary malignant neoplasm of testis
Metastatic cancer	B58y700	Secondary malignant neoplasm of penis
Metastatic cancer	B58y800	Second malignant neoplasm of epididymis/vas deferens
Metastatic cancer	B58y900	Secondary malignant neoplasm of tongue
Metastatic cancer	B58yz00	Secondary malignant neoplasm of other specified site
Metastatic cancer	B58z.00	Secondary malignant neoplasm of other specified site
Metastatic cancer	B594.00	Secondary malignant neoplasm of unknown site
Metastatic cancer	BB03.00	[M]Neoplasm, metastatic
Metastatic cancer	BB03.11	[M]Secondary neoplasm
Metastatic cancer	BB13.00	[M]Carcinoma, metastatic, NOS
Metastatic cancer	BB13.11	[M]Secondary carcinoma
Metastatic cancer	ByuC200	[X]2ndry+unspcf malignant neoplasm LN/multi regions

Metastatic cancer	ByuC300	[X]Second malignant neopl/oth+unspc respiratory organs
Metastatic cancer	ByuC400	[X]Second malignant neopl/oth+unspcfd digestive organs
Metastatic cancer	ByuC500	[X]2ndry malignant neopl/bladder+oth+unsp urina organs
Metastatic cancer	ByuC600	[X]2ndry malignant neopl/oth+unspec parts/nervous syst
Metastatic cancer	ByuC700	[X]Secondary malignant neoplasm of other specified sites
Mild liver dis	9kR..00	Chronic hepatitis annual review -enhanced services admin
Mild liver dis	A707.00	Chronic viral hepatitis
Mild liver dis	A707000	Chronic viral hepatitis B with delta-agent
Mild liver dis	A707100	Chronic viral hepatitis B without delta-agent
Mild liver dis	A707200	Chronic viral hepatitis C
Mild liver dis	A707300	Chronic viral hepatitis B
Mild liver dis	A707X00	Chronic viral hepatitis, unspecified
Mild liver dis	C310200	Hepatorenal glycogenosis
Mild liver dis	C310400	Glycogenosis with hepatic cirrhosis
Mild liver dis	C350012	Pigmentary cirrhosis of liver
Mild liver dis	C370800	Cystic fibrosis related cirrhosis
Mild liver dis	J61..00	Cirrhosis and chronic liver dis
Mild liver dis	J612.00	Alcoholic cirrhosis of liver
Mild liver dis	J612.11	Florid cirrhosis
Mild liver dis	J612.12	Laennec's cirrhosis
Mild liver dis	J614.00	Chronic hepatitis
Mild liver dis	J614000	Chronic persistent hepatitis
Mild liver dis	J614100	Chronic active hepatitis
Mild liver dis	J614111	Autoimmune chronic active hepatitis
Mild liver dis	J614200	Chronic aggressive hepatitis
Mild liver dis	J614300	Recurrent hepatitis
Mild liver dis	J614400	Chronic lobular hepatitis
Mild liver dis	J614y00	Chronic hepatitis unspecified
Mild liver dis	J614z00	Chronic hepatitis NOS
Mild liver dis	J615.00	Cirrhosis - non alcoholic
Mild liver dis	J615.11	Portal cirrhosis
Mild liver dis	J615100	Multilobular portal cirrhosis
Mild liver dis	J615300	Diffuse nodular cirrhosis
Mild liver dis	J615400	Fatty portal cirrhosis
Mild liver dis	J615500	Hypertrophic portal cirrhosis
Mild liver dis	J615600	Capsular portal cirrhosis
Mild liver dis	J615700	Cardiac portal cirrhosis
Mild liver dis	J615711	Cong cirrhosis
Mild liver dis	J615800	Juvenile portal cirrhosis
Mild liver dis	J615812	Indian childhood cirrhosis
Mild liver dis	J615C00	Xanthomatous portal cirrhosis
Mild liver dis	J615D00	Bacterial portal cirrhosis
Mild liver dis	J615H00	Infectious cirrhosis NOS
Mild liver dis	J615y00	Portal cirrhosis unspecified

Mild liver dis	J615z00	Non-alcoholic cirrhosis NOS
Mild liver dis	J615z11	Macronodular cirrhosis of liver
Mild liver dis	J615z12	Cryptogenic cirrhosis of liver
Mild liver dis	J615z13	Cirrhosis of liver NOS
Mild liver dis	J616.00	Biliary cirrhosis
Mild liver dis	J616000	Primary biliary cirrhosis
Mild liver dis	J616100	Secondary biliary cirrhosis
Mild liver dis	J616200	Biliary cirrhosis of children
Mild liver dis	J616z00	Biliary cirrhosis NOS
Mild liver dis	J617000	Chronic alcoholic hepatitis
Mild liver dis	J635300	Toxic liver dis with chronic persistent hepatitis
Mild liver dis	J635400	Toxic liver dis with chronic lobular hepatitis
Mild liver dis	J635500	Toxic liver dis with chronic active hepatitis
Mild liver dis	J635600	Toxic liver dis with fibrosis and cirrhosis of liver
Mild liver dis	Jyu7100	[X]Other and unspecified cirrhosis of liver
Mod/severe liver dis	7800	Transplantation of liver
Mod/severe liver dis	7800000	Orthotopic transplantation of liver
Mod/severe liver dis	7800100	Heterotopic transplantation of liver
Mod/severe liver dis	7800111	Auxillary liver transplant
Mod/severe liver dis	7800112	Piggy back liver transplant
Mod/severe liver dis	7800200	Replacement of previous liver transplant
Mod/severe liver dis	7800400	Orthotopic transplantation of whole liver
Mod/severe liver dis	7800500	Orthotopic transplantation of liver NEC
Mod/severe liver dis	7800y00	Other specified transplantation of liver
Mod/severe liver dis	7800z00	Transplantation of liver NOS
Mod/severe liver dis	G850.00	Oesophageal varices with bleeding
Mod/severe liver dis	G851.00	Oesophageal varices without bleeding
Mod/severe liver dis	G852.00	Oesophageal varices in diss EC
Mod/severe liver dis	G852000	Oesophageal varices with bleeding in diss EC
Mod/severe liver dis	G852100	Oesophageal varices without bleeding in diss EC
Mod/severe liver dis	G852200	Oesophageal varices in cirrhosis of the liver
Mod/severe liver dis	G852300	Oesophageal varices in alcoholic cirrhosis of the liver
Mod/severe liver dis	G852z00	Oesophageal varices in diss EC NOS
Mod/severe liver dis	G858.00	Oesophageal varices NOS
Mod/severe liver dis	J622.00	Hepatic coma
Mod/severe liver dis	J622.11	Encephalopathy - hepatic
Mod/severe liver dis	J623.00	Portal hypertension
Mod/severe liver dis	J624.00	Hepatorenal synde
Mod/severe liver dis	SP08600	Liver transplant failure and rejection
Mod/severe liver dis	ZV42700	[V]Liver transplanted
Mod/Severe Renal dis	14V2.00	H/O: renal dialysis
Mod/Severe Renal dis	14V2.11	H/O: kidney dialysis
Mod/Severe Renal dis	1Z1..00	Chronic renal impairment
Mod/Severe Renal dis	1Z10.00	Chronic kidney dis stage 1

Mod/Severe Renal dis	1Z11.00	Chronic kidney dis stage 2
Mod/Severe Renal dis	1Z12.00	Chronic kidney dis stage 3
Mod/Severe Renal dis	1Z13.00	Chronic kidney dis stage 4
Mod/Severe Renal dis	1Z14.00	Chronic kidney dis stage 5
Mod/Severe Renal dis	1Z15.00	Chronic kidney dis stage 3A
Mod/Severe Renal dis	1Z16.00	Chronic kidney dis stage 3B
Mod/Severe Renal dis	1Z17.00	Chronic kidney dis stage 1 with proteinuria
Mod/Severe Renal dis	1Z17.11	CKD stage 1 with proteinuria
Mod/Severe Renal dis	1Z18.00	Chronic kidney dis stage 1 without proteinuria
Mod/Severe Renal dis	1Z19.00	Chronic kidney dis stage 2 with proteinuria
Mod/Severe Renal dis	1Z19.11	CKD stage 2 with proteinuria
Mod/Severe Renal dis	1Z1A.00	Chronic kidney dis stage 2 without proteinuria
Mod/Severe Renal dis	1Z1A.11	CKD stage 2 without proteinuria
Mod/Severe Renal dis	1Z1B.00	Chronic kidney dis stage 3 with proteinuria
Mod/Severe Renal dis	1Z1B.11	CKD stage 3 with proteinuria
Mod/Severe Renal dis	1Z1C.00	Chronic kidney dis stage 3 without proteinuria
Mod/Severe Renal dis	1Z1C.11	CKD stage 3 without proteinuria
Mod/Severe Renal dis	1Z1D.00	Chronic kidney dis stage 3A with proteinuria
Mod/Severe Renal dis	1Z1D.11	CKD stage 3A with proteinuria
Mod/Severe Renal dis	1Z1E.00	Chronic kidney dis stage 3A without proteinuria
Mod/Severe Renal dis	1Z1E.11	CKD stage 3A without proteinuria
Mod/Severe Renal dis	1Z1F.00	Chronic kidney dis stage 3B with proteinuria
Mod/Severe Renal dis	1Z1F.11	CKD stage 3B with proteinuria
Mod/Severe Renal dis	1Z1G.00	Chronic kidney dis stage 3B without proteinuria
Mod/Severe Renal dis	1Z1G.11	CKD stage 3B without proteinuria
Mod/Severe Renal dis	1Z1H.00	Chronic kidney dis stage 4 with proteinuria
Mod/Severe Renal dis	1Z1H.11	CKD stage 4 with proteinuria
Mod/Severe Renal dis	1Z1J.00	Chronic kidney dis stage 4 without proteinuria
Mod/Severe Renal dis	1Z1J.11	CKD stage 4 without proteinuria
Mod/Severe Renal dis	1Z1K.00	Chronic kidney dis stage 5 with proteinuria
Mod/Severe Renal dis	1Z1K.11	CKD stage 5 with proteinuria
Mod/Severe Renal dis	1Z1L.00	Chronic kidney dis stage 5 without proteinuria
Mod/Severe Renal dis	1Z1L.11	CKD stage 5 without proteinuria
Mod/Severe Renal dis	66i..00	Chronic kidney dis monitoring
Mod/Severe Renal dis	7A60600	Creation of graft fistula for dialysis
Mod/Severe Renal dis	7A61900	Ligation of arteriovenous dialysis fistula
Mod/Severe Renal dis	7A61A00	Ligation of arteriovenous dialysis graft
Mod/Severe Renal dis	7B00.00	Transplantation of kidney
Mod/Severe Renal dis	7B00000	Autotransplant of kidney
Mod/Severe Renal dis	7B00100	Transplantation of kidney from live donor
Mod/Severe Renal dis	7B00111	Allotransplantation of kidney from live donor
Mod/Severe Renal dis	7B00200	Transplantation of kidney from cadaver
Mod/Severe Renal dis	7B00211	Allotransplantation of kidney from cadaver
Mod/Severe Renal dis	7B00212	Cadaveric renal transplant

Mod/Severe Renal dis	7B00300	Allotransplantation of kidney from cadaver, heart-beating
Mod/Severe Renal dis	7B00400	Allotransplantat kidney from cadaver, heart nonbeating
Mod/Severe Renal dis	7B00600	Xenograft renal transplant
Mod/Severe Renal dis	7B00y00	Other specified transplantation of kidney
Mod/Severe Renal dis	7B00z00	Transplantation of kidney NOS
Mod/Severe Renal dis	7B01500	Transplant nephrectomy
Mod/Severe Renal dis	7B01511	Excision of rejected transplanted kidney
Mod/Severe Renal dis	7B0F.00	Interventions associated with transplantation of kidney
Mod/Severe Renal dis	7B0F100	Pre-transplantation of kidney work-up, recipient
Mod/Severe Renal dis	7B0F200	Pre-transplantation of kidney work-up, live donor
Mod/Severe Renal dis	7B0F300	Post-transplantation of kidney examination, recipient
Mod/Severe Renal dis	7B0F400	Post-transplantation of kidney examination, live donor
Mod/Severe Renal dis	7B0Fy00	OS interventions associa with transplantation of kidney
Mod/Severe Renal dis	7B0Fz00	Interventions associated with transplantation of kidney
Mod/Severe Renal dis	7L1A.00	Compensation for renal failure
Mod/Severe Renal dis	7L1A.11	Dialysis for renal failure
Mod/Severe Renal dis	7L1A000	Renal dialysis
Mod/Severe Renal dis	7L1A011	Thomas intravascular shunt for dialysis
Mod/Severe Renal dis	7L1A100	Peritoneal dialysis
Mod/Severe Renal dis	7L1A200	Haemodialysis NEC
Mod/Severe Renal dis	7L1A300	Haemofiltration
Mod/Severe Renal dis	7L1A400	Automated peritoneal dialysis
Mod/Severe Renal dis	7L1A500	Continuous ambulatory peritoneal dialysis
Mod/Severe Renal dis	7L1A600	Peritoneal dialysis NEC
Mod/Severe Renal dis	7L1Ay00	Other specified compensation for renal failure
Mod/Severe Renal dis	7L1Az00	Compensation for renal failure NOS
Mod/Severe Renal dis	7L1B.00	Placement ambulatory apparatus compensation renal fail
Mod/Severe Renal dis	7L1B.11	Placemt ambulatory dialysis apparatus-compens ren fail
Mod/Severe Renal dis	7L1B000	Insertion of ambulatory peritoneal dialysis catheter
Mod/Severe Renal dis	7L1B100	Removal of ambulatory peritoneal dialysis catheter
Mod/Severe Renal dis	7L1B200	Flushing of peritoneal dialysis catheter
Mod/Severe Renal dis	7L1By00	Placemt ambulatory apparatus-compensate renal fail OS
Mod/Severe Renal dis	7L1C.00	Placement other apparatus for compensation for renal fail
Mod/Severe Renal dis	7L1C000	Insertion of temporary peritoneal dialysis catheter
Mod/Severe Renal dis	7L1Cy00	Placement other apparatus- compensate for renal fail OS
Mod/Severe Renal dis	7L1Cz00	Placement other apparatus- compensate for renal failure
Mod/Severe Renal dis	9Ot..00	Chronic kidney dis monitoring administration
Mod/Severe Renal dis	9Ot0.00	Chronic kidney dis monitoring first letter
Mod/Severe Renal dis	9Ot1.00	Chronic kidney dis monitoring second letter
Mod/Severe Renal dis	9Ot2.00	Chronic kidney dis monitoring third letter
Mod/Severe Renal dis	9Ot3.00	Chronic kidney dis monitoring verbal invite
Mod/Severe Renal dis	9Ot4.00	Chronic kidney dis monitoring telephone invite
Mod/Severe Renal dis	9Ot5.00	Predicted stage chronic kidney dis
Mod/Severe Renal dis	D215000	Anaemia secondary to chronic renal failure

Mod/Severe Renal dis	F374A00	Polyneuropathy in uraemia
Mod/Severe Renal dis	G500400	Acute pericarditis - uraemic
Mod/Severe Renal dis	G72C.00	Ruptured aneurysm of dialysis vascular access
Mod/Severe Renal dis	G72D.00	Aneurysm of dialysis arteriovenous fistula
Mod/Severe Renal dis	G72D100	Aneurysm of needle site of dialysis arteriovenous fistula
Mod/Severe Renal dis	G72D200	Aneurysm of anastomotic site of dialysis AV fistula
Mod/Severe Renal dis	Gy1..00	Stenosis of dialysis vascular access
Mod/Severe Renal dis	Gy10.00	Stenosis of dialysis arteriovenous graft
Mod/Severe Renal dis	Gy21.00	Thrombosis of dialysis arteriovenous fistula
Mod/Severe Renal dis	Gy3..00	Occlusion of dialysis vascular access
Mod/Severe Renal dis	Gy30.00	Occlusion of dialysis arteriovenous graft
Mod/Severe Renal dis	Gy31.00	Occlusion of dialysis arteriovenous fistula
Mod/Severe Renal dis	Gy40.00	Infection of dialysis arteriovenous graft
Mod/Severe Renal dis	Gy41.00	Infection of dialysis arteriovenous fistula
Mod/Severe Renal dis	Gy5..00	Haemorrhage of dialysis vascular access
Mod/Severe Renal dis	Gy51.00	Haemorrhage of dialysis arteriovenous fistula
Mod/Severe Renal dis	Gy60.00	Rupture of dialysis arteriovenous graft
Mod/Severe Renal dis	K02..00	Chronic glomerulonephritis
Mod/Severe Renal dis	K02..11	Nephritis - chronic
Mod/Severe Renal dis	K02..12	Nephropathy - chronic
Mod/Severe Renal dis	K020.00	Chronic proliferative glomerulonephritis
Mod/Severe Renal dis	K021.00	Chronic membranous glomerulonephritis
Mod/Severe Renal dis	K022.00	Chronic membranoproliferative glomerulonephritis
Mod/Severe Renal dis	K023.00	Chronic rapidly progressive glomerulonephritis
Mod/Severe Renal dis	K02y.00	Other chronic glomerulonephritis
Mod/Severe Renal dis	K02y000	Chronic glomerulonephritis + diss EC
Mod/Severe Renal dis	K02y200	Chronic focal glomerulonephritis
Mod/Severe Renal dis	K02y300	Chronic diffuse glomerulonephritis
Mod/Severe Renal dis	K02yz00	Other chronic glomerulonephritis NOS
Mod/Severe Renal dis	K02z.00	Chronic glomerulonephritis NOS
Mod/Severe Renal dis	K05..00	Chronic renal failure
Mod/Severe Renal dis	K05..11	Chronic uraemia
Mod/Severe Renal dis	K05..12	End stage renal failure
Mod/Severe Renal dis	K05..13	Chronic kidney dis
Mod/Severe Renal dis	K050.00	End stage renal failure
Mod/Severe Renal dis	K051.00	Chronic kidney dis stage 1
Mod/Severe Renal dis	K052.00	Chronic kidney dis stage 2
Mod/Severe Renal dis	K053.00	Chronic kidney dis stage 3
Mod/Severe Renal dis	K054.00	Chronic kidney dis stage 4
Mod/Severe Renal dis	K055.00	Chronic kidney dis stage 5
Mod/Severe Renal dis	K06..00	Renal failure unspecified
Mod/Severe Renal dis	K06..11	Uraemia NOS
Mod/Severe Renal dis	K06..12	Kidney failure unspecified
Mod/Severe Renal dis	K060.00	Renal impairment

Mod/Severe Renal dis	K060.11	Impaired renal function
Mod/Severe Renal dis	K0A3.00	Chronic nephritic synde
Mod/Severe Renal dis	K0A3000	Chronic nephritic synde, minor glomerular abnormality
Mod/Severe Renal dis	K0A3100	Chronic nephritic syndrm focal+segmental glomerular les
Mod/Severe Renal dis	K0A3200	Chron nephritic synd difuse membranous glomerulonephr
Mod/Severe Renal dis	K0A3300	Chron neph syn difus mesangial prolifrtiv glomerulonephr
Mod/Severe Renal dis	K0A3500	Chronic neph syn difus mesangiocapillary glomerulonephr
Mod/Severe Renal dis	K0A3600	Chronic nephritic synde, dense deposit dis
Mod/Severe Renal dis	K0A3700	Chronic nephritic syn diffuse crescentic glomerulonephr
Mod/Severe Renal dis	K0B5.00	Renal tubulo-interstitial disordrs in transplant rejectn
Mod/Severe Renal dis	K0D..00	End-stage renal dis
Mod/Severe Renal dis	K0E..00	Acute-on-chronic renal failure
Mod/Severe Renal dis	K105.00	Chronic infective interstitial nephritis
Mod/Severe Renal dis	K13C000	Chronic cyclosporin A nephrotoxicity
Mod/Severe Renal dis	Kyu1000	[X]Other chronic tubulo-interstitial nephritis
Mod/Severe Renal dis	Kyu1C00	[X]Renal tubulo-interstitial disorders/transplant rejection
Mod/Severe Renal dis	Kyu2.00	[X]Renal failure
Mod/Severe Renal dis	Kyu2100	[X]Other chronic renal failure
Mod/Severe Renal dis	SP01500	Mechanical complication of dialysis catheter
Mod/Severe Renal dis	SP05613	[X] Peritoneal dialysis associated peritonitis
Mod/Severe Renal dis	SP06B00	Continuous ambulatory peritoneal dialysis associat perit
Mod/Severe Renal dis	SP07G00	Stenosis of arteriovenous dialysis fistula
Mod/Severe Renal dis	SP08011	Det.ren.func.after ren.transpl
Mod/Severe Renal dis	SP08300	Kidney transplant failure and rejection
Mod/Severe Renal dis	SP08D00	Acute-on-chronic rejection of renal transplant
Mod/Severe Renal dis	SP08E00	Acute rejection of renal transplant - grade I
Mod/Severe Renal dis	SP08F00	Acute rejection of renal transplant - grade II
Mod/Severe Renal dis	SP08G00	Acute rejection of renal transplant - grade III
Mod/Severe Renal dis	SP08H00	Acute rejection of renal transplant
Mod/Severe Renal dis	SP08J00	Chronic rejection of renal transplant
Mod/Severe Renal dis	SP08N00	Unexplained episode of renal transplant dysfunction
Mod/Severe Renal dis	SP08P00	Stenosis of vein of transplanted kidney
Mod/Severe Renal dis	SP08R00	Renal transplant rejection
Mod/Severe Renal dis	SP08T00	Urological complication of renal transplant
Mod/Severe Renal dis	SP08V00	Very mild acute rejection of renal transplant
Mod/Severe Renal dis	SP08V11	Borderline changes of acute rejection
Mod/Severe Renal dis	SP08W00	Vascular complication of renal transplant
Mod/Severe Renal dis	SP0E.00	Disorders associated with peritoneal dialysis
Mod/Severe Renal dis	SP0F.00	Haemodialysis first use synde
Mod/Severe Renal dis	SP0G.00	Anaphylactoid reaction due to haemodialysis
Mod/Severe Renal dis	TA02000	Accid cut,puncture,perf,h'ge - kidney dialysis
Mod/Severe Renal dis	TA22000	Failure of sterile precautions during kidney dialysis
Mod/Severe Renal dis	TB00100	Kidney transplant with complication, without blame
Mod/Severe Renal dis	TB00111	Renal transplant with complication, without blame

Mod/Severe Renal dis	TB11.00	Kidney dialysis with complication, without blame
Mod/Severe Renal dis	TB11.11	Renal dialysis with complication, without blame
Mod/Severe Renal dis	ZV42000	[V]Kidney transplanted
Mod/Severe Renal dis	ZV45100	[V]Renal dialysis status
Mod/Severe Renal dis	ZV56.00	[V]Aftercare involving intermittent dialysis
Mod/Severe Renal dis	ZV56000	[V]Aftercare involving extracorporeal dialysis
Mod/Severe Renal dis	ZV56011	[V]Aftercare involving renal dialysis NOS
Mod/Severe Renal dis	ZV56100	[V]Preparatory care for dialysis
Mod/Severe Renal dis	ZV56y00	[V]Other specified aftercare involving intermittent dialysis
Mod/Severe Renal dis	ZV56y11	[V]Aftercare involving peritoneal dialysis
Mod/Severe Renal dis	ZV56z00	[V]Unspecified aftercare involving intermittent dialysis
Myocardial infarction	14A3.00	H/O: myocardial infarct <60
Myocardial infarction	14A4.00	H/O: myocardial infarct >60
Myocardial infarction	14AH.00	H/O: Myocardial infarction in last year
Myocardial infarction	14AT.00	History of myocardial infarction
Myocardial infarction	G30..00	Acute myocardial infarction
Myocardial infarction	G30..11	Attack - heart
Myocardial infarction	G30..12	Coronary thrombosis
Myocardial infarction	G30..13	Cardiac rupture following myocardial infarction (MI)
Myocardial infarction	G30..14	Heart attack
Myocardial infarction	G30..15	MI - acute myocardial infarction
Myocardial infarction	G30..16	Thrombosis - coronary
Myocardial infarction	G30..17	Silent myocardial infarction
Myocardial infarction	G300.00	Acute anterolateral infarction
Myocardial infarction	G301.00	Other specified anterior myocardial infarction
Myocardial infarction	G301000	Acute anteroapical infarction
Myocardial infarction	G301100	Acute anteroseptal infarction
Myocardial infarction	G301z00	Anterior myocardial infarction NOS
Myocardial infarction	G302.00	Acute inferolateral infarction
Myocardial infarction	G303.00	Acute inferoposterior infarction
Myocardial infarction	G304.00	Posterior myocardial infarction NOS
Myocardial infarction	G305.00	Lateral myocardial infarction NOS
Myocardial infarction	G306.00	True posterior myocardial infarction
Myocardial infarction	G307.00	Acute subendocardial infarction
Myocardial infarction	G307000	Acute non-Q wave infarction
Myocardial infarction	G307100	Acute non-ST segment elevation myocardial infarction
Myocardial infarction	G308.00	Inferior myocardial infarction NOS
Myocardial infarction	G309.00	Acute Q-wave infarct
Myocardial infarction	G30B.00	Acute posterolateral myocardial infarction
Myocardial infarction	G30X.00	Acute transmural myocardial infarction of unspecif site
Myocardial infarction	G30X000	Acute ST segment elevation myocardial infarction
Myocardial infarction	G30y.00	Other acute myocardial infarction
Myocardial infarction	G30y000	Acute atrial infarction
Myocardial infarction	G30y100	Acute papillary muscle infarction

Myocardial infarction	G30y200	Acute septal infarction
Myocardial infarction	G30yz00	Other acute myocardial infarction NOS
Myocardial infarction	G30z.00	Acute myocardial infarction NOS
Myocardial infarction	G310.00	Postmyocardial infarction synde
Myocardial infarction	G310.11	Dressler's synde
Myocardial infarction	G31y100	Microinfarction of heart
Myocardial infarction	G32..00	Old myocardial infarction
Myocardial infarction	G32..11	Healed myocardial infarction
Myocardial infarction	G32..12	Personal history of myocardial infarction
Myocardial infarction	G35..00	Subsequent myocardial infarction
Myocardial infarction	G350.00	Subsequent myocardial infarction of anterior wall
Myocardial infarction	G351.00	Subsequent myocardial infarction of inferior wall
Myocardial infarction	G353.00	Subsequent myocardial infarction of other sites
Myocardial infarction	G35X.00	Subsequent myocardial infarction of unspecified site
Myocardial infarction	G36..00	Current complication follow acute myocardial infarct
Myocardial infarction	G360.00	Haemopericard/currt comp folow acut myocard infarct
Myocardial infarction	G361.00	Atrial septal defect/curr comp folow acut myoc infarct
Myocardial infarction	G362.00	Ventric septal defect/curr comp fol acut myoc infarctn
Myocardial infarction	G363.00	Ruptur cardiac wall haemopericard/cur comp fol ac MI
Myocardial infarction	G364.00	Ruptur chordae tendinae/curr comp fol acute myoc infar
Myocardial infarction	G365.00	Ruptur papillary muscle/curr comp fol acte myocard infar
Myocardial infarction	G366.00	Thrbsis atrium,auric append&vent/curr comp foll acte MI
Myocardial infarction	G38..00	Postoperative myocardial infarction
Myocardial infarction	G380.00	Postoperative transmural myocard infarction anterior wall
Myocardial infarction	G381.00	Postoperative transmural myocard infarction inferior wall
Myocardial infarction	G383.00	Postoperative transmural myocardial infarction unspec si
Myocardial infarction	G384.00	Postoperative subendocardial myocardial infarction
Myocardial infarction	G38z.00	Postoperative myocardial infarction, unspecified
Myocardial infarction	G501.00	Post infarction pericarditis
Myocardial infarction	Gyu3400	[X]Acute transmural myocardial infarction of unspecif site
Myocardial infarction	Gyu3500	[X]Subsequent myocardial infarction of other sites
Myocardial infarction	Gyu3600	[X]Subsequent myocardial infarction of unspecified site
Peptic ulcer dis	J102.00	Ulcer of oesophagus
Peptic ulcer dis	J102000	Peptic ulcer of oesophagus
Peptic ulcer dis	J102100	Fungal ulcer of oesophagus
Peptic ulcer dis	J102200	Oesophageal ulcer due to aspirin
Peptic ulcer dis	J102300	Oesophageal ulcer due to chemicals
Peptic ulcer dis	J102400	Oesophageal ulcer due to medicines
Peptic ulcer dis	J102500	Barrett's ulcer of oesophagus
Peptic ulcer dis	J102z00	Ulcer of oesophagus NOS
Peptic ulcer dis	J11..00	Gastric ulcer - (GU)
Peptic ulcer dis	J11..11	Prepyloric ulcer
Peptic ulcer dis	J11..12	Pyloric ulcer
Peptic ulcer dis	J110.00	Acute gastric ulcer

Peptic ulcer dis	J110000	Acute gastric ulcer without mention of complication
Peptic ulcer dis	J110100	Acute gastric ulcer with haemorrhage
Peptic ulcer dis	J110111	Bleeding acute gastric ulcer
Peptic ulcer dis	J110200	Acute gastric ulcer with perforation
Peptic ulcer dis	J110300	Acute gastric ulcer with haemorrhage and perforation
Peptic ulcer dis	J110y00	Acute gastric ulcer unspecified
Peptic ulcer dis	J110z00	Acute gastric ulcer NOS
Peptic ulcer dis	J111.00	Chronic gastric ulcer
Peptic ulcer dis	J111000	Chronic gastric ulcer without mention of complication
Peptic ulcer dis	J111100	Chronic gastric ulcer with haemorrhage
Peptic ulcer dis	J111111	Bleeding chronic gastric ulcer
Peptic ulcer dis	J111200	Chronic gastric ulcer with perforation
Peptic ulcer dis	J111211	Perforated chronic gastric ulcer
Peptic ulcer dis	J111300	Chronic gastric ulcer with haemorrhage and perforation
Peptic ulcer dis	J111400	Chronic gastric ulcer with obstruction
Peptic ulcer dis	J111y00	Chronic gastric ulcer unspecified
Peptic ulcer dis	J111z00	Chronic gastric ulcer NOS
Peptic ulcer dis	J112.00	Anti-platelet induced gastric ulcer
Peptic ulcer dis	J112z00	Anti-platelet induced gastric ulcer NOS
Peptic ulcer dis	J113.00	Non steroidal antiinflammatory drug induced gastric ulcer
Peptic ulcer dis	J113z00	Non steroidal antiinflammatory drug induced gastric ulc
Peptic ulcer dis	J11y.00	Unspecified gastric ulcer
Peptic ulcer dis	J11y000	Unspecified gastric ulcer without mention of complication
Peptic ulcer dis	J11y100	Unspecified gastric ulcer with haemorrhage
Peptic ulcer dis	J11y200	Unspecified gastric ulcer with perforation
Peptic ulcer dis	J11y400	Unspecified gastric ulcer with obstruction
Peptic ulcer dis	J11yy00	Unspec gastric ulcer, haemorrhage and/or perforation
Peptic ulcer dis	J11yz00	Unspecified gastric ulcer NOS
Peptic ulcer dis	J11z.00	Gastric ulcer NOS
Peptic ulcer dis	J11z.11	Gastric erosions
Peptic ulcer dis	J11z.12	Multiple gastric ulcers
Peptic ulcer dis	J12..00	Duodenal ulcer - (DU)
Peptic ulcer dis	J120.00	Acute duodenal ulcer
Peptic ulcer dis	J120000	Acute duodenal ulcer without mention of complication
Peptic ulcer dis	J120100	Acute duodenal ulcer with haemorrhage
Peptic ulcer dis	J120200	Acute duodenal ulcer with perforation
Peptic ulcer dis	J120300	Acute duodenal ulcer with haemorrhage and perforation
Peptic ulcer dis	J120400	Acute duodenal ulcer with obstruction
Peptic ulcer dis	J120y00	Acute duodenal ulcer unspecified
Peptic ulcer dis	J120z00	Acute duodenal ulcer NOS
Peptic ulcer dis	J121.00	Chronic duodenal ulcer
Peptic ulcer dis	J121000	Chronic duodenal ulcer without mention of complication
Peptic ulcer dis	J121100	Chronic duodenal ulcer with haemorrhage
Peptic ulcer dis	J121111	Bleeding chronic duodenal ulcer

Peptic ulcer dis	J121200	Chronic duodenal ulcer with perforation
Peptic ulcer dis	J121211	Perforated chronic duodenal ulcer
Peptic ulcer dis	J121300	Chronic duodenal ulcer with haemorrhage/perforation
Peptic ulcer dis	J121400	Chronic duodenal ulcer with obstruction
Peptic ulcer dis	J121y00	Chronic duodenal ulcer unspecified
Peptic ulcer dis	J121z00	Chronic duodenal ulcer NOS
Peptic ulcer dis	J122.00	Duodenal ulcer dis
Peptic ulcer dis	J123.00	Duodenal erosion
Peptic ulcer dis	J124.00	Recurrent duodenal ulcer
Peptic ulcer dis	J125.00	Anti-platelet induced duodenal ulcer
Peptic ulcer dis	J126.00	Nonsteroidal antiinflammatory drug induced duodl ulcer
Peptic ulcer dis	J12y.00	Unspecified duodenal ulcer
Peptic ulcer dis	J12y000	Unspecified duodenal ulcer without mention of complic
Peptic ulcer dis	J12y100	Unspecified duodenal ulcer with haemorrhage
Peptic ulcer dis	J12y200	Unspecified duodenal ulcer with perforation
Peptic ulcer dis	J12y300	Unspec duodenal ulcer with haemorrhage and perforation
Peptic ulcer dis	J12y400	Unspecified duodenal ulcer with obstruction
Peptic ulcer dis	J12yy00	Unspec duodenal ulcer, haemorrhage and/or perforation
Peptic ulcer dis	J12yz00	Unspecified duodenal ulcer NOS
Peptic ulcer dis	J12z.00	Duodenal ulcer NOS
Peptic ulcer dis	J13..00	Peptic ulcer - (PU) site unspecified
Peptic ulcer dis	J13..11	Stress ulcer NOS
Peptic ulcer dis	J130.00	Acute peptic ulcer
Peptic ulcer dis	J130000	Acute peptic ulcer without mention of complication
Peptic ulcer dis	J130100	Acute peptic ulcer with haemorrhage
Peptic ulcer dis	J130200	Acute peptic ulcer with perforation
Peptic ulcer dis	J130300	Acute peptic ulcer with haemorrhage and perforation
Peptic ulcer dis	J130y00	Acute peptic ulcer unspecified
Peptic ulcer dis	J130z00	Acute peptic ulcer NOS
Peptic ulcer dis	J131.00	Chronic peptic ulcer
Peptic ulcer dis	J131000	Chronic peptic ulcer without mention of complication
Peptic ulcer dis	J131100	Chronic peptic ulcer with haemorrhage
Peptic ulcer dis	J131200	Chronic peptic ulcer with perforation
Peptic ulcer dis	J131400	Chronic peptic ulcer with obstruction
Peptic ulcer dis	J131y00	Chronic peptic ulcer unspecified
Peptic ulcer dis	J131z00	Chronic peptic ulcer NOS
Peptic ulcer dis	J13y.00	Unspecified peptic ulcer
Peptic ulcer dis	J13y000	Unspecified peptic ulcer without mention of complication
Peptic ulcer dis	J13y100	Unspecified peptic ulcer with haemorrhage
Peptic ulcer dis	J13y200	Unspecified peptic ulcer with perforation
Peptic ulcer dis	J13y300	Unspecified peptic ulcer with haemorrhage/perforation
Peptic ulcer dis	J13y400	Unspecified peptic ulcer with obstruction
Peptic ulcer dis	J13yz00	Unspecified peptic ulcer NOS
Peptic ulcer dis	J13z.00	Peptic ulcer NOS

Peripheral vascular dis	14AE.00	H/O: aortic aneurysm
Peripheral vascular dis	14NB.00	H/O: Peripheral vascular dis procedure
Peripheral vascular dis	16I..00	Claudication distance
Peripheral vascular dis	2I16.00	O/E - gangrene
Peripheral vascular dis	662U.00	Peripheral vascular dis monitoring
Peripheral vascular dis	66f3.00	Aortic aneurysm monitoring
Peripheral vascular dis	7A11.00	Replacement of aneurysmal bifurcation of aorta
Peripheral vascular dis	7A11000	Emg repl aneurysm bifurc aorta by anast aorta to fem art
Peripheral vascular dis	7A11100	Repl aneurysm bifurc aorta by anast aorta to femoral art
Peripheral vascular dis	7A11200	Emerg repl aneurysm bifurc aorta by anast aorta to iliac a
Peripheral vascular dis	7A11211	Y graft of abdominal Aortic aneurysm (emergency)
Peripheral vascular dis	7A11300	Repl aneurysm bifurc aorta by anast aorta to iliac artery
Peripheral vascular dis	7A11311	Y graft abdominal Aortic aneurysm
Peripheral vascular dis	7A11y00	Replacement of aneurysmal bifurcation of aorta OS
Peripheral vascular dis	7A11z00	Replacement of aneurysmal bifurcation of aorta NOS
Peripheral vascular dis	7A13.00	Emergency replacement of aneurysmal segment of aorta
Peripheral vascular dis	7A13.11	Emergency repair of aortic aneurysm
Peripheral vascular dis	7A13000	Emerg repl aneurysm asc aorta by anastom aorta to aorta
Peripheral vascular dis	7A13100	Emg repl aneurysm thor aorta by anastom aorta to aorta
Peripheral vascular dis	7A13300	Emg repl aneurysm infrarenal aorta by anast aorta/aorta
Peripheral vascular dis	7A13400	Emg repl aneurysm abdom aorta by anast aorta/aortaNEC
Peripheral vascular dis	7A13411	Tube graft abdominal Aortic aneurysm (emergency)
Peripheral vascular dis	7A13y00	Emergency replacement of aneurysmal segment of aorta
Peripheral vascular dis	7A13z00	Emergency replacement of aneurysmal segment of aorta
Peripheral vascular dis	7A14.00	Other replacement of aneurysmal segment of aorta
Peripheral vascular dis	7A14.11	Aortic aneurysm repair
Peripheral vascular dis	7A14000	Repl aneurysm ascend aorta by anast of aorta/aorta NEC
Peripheral vascular dis	7A14100	Repl aneurysm thoracic aorta by anast of aorta/aorta NEC
Peripheral vascular dis	7A14200	Repl aneurys suprarenal aorta by anast aorta to aorta NEC
Peripheral vascular dis	7A14300	Repl aneurys infrarenal aorta by anast aorta to aorta NEC
Peripheral vascular dis	7A14400	Repl aneurysm abdo aorta by anast aorta to aortaNEC
Peripheral vascular dis	7A14411	Tube graft of Abdominal aortic aneurysm
Peripheral vascular dis	7A14y00	Other replacement of aneurysmal segment of aorta OS
Peripheral vascular dis	7A14z00	Other replacement of aneurysmal segment of aorta NOS
Peripheral vascular dis	7A19400	Operation on aneurysm of aorta NEC
Peripheral vascular dis	7A1B.00	Transluminal operations on aneurysmal segment of aorta
Peripheral vascular dis	7A1B000	Endovasstenting infrarenal abdominal aortic aneurysm
Peripheral vascular dis	7A1B100	Endovasstenting of suprarenal aortic aneurysm
Peripheral vascular dis	7A1B200	Endovasstenting of thoracic aortic aneurysm
Peripheral vascular dis	7A1B500	Endovasstenting of aorto-uniiliac aneurysm
Peripheral vascular dis	7A1B600	Endovasc stenting for aortic aneurysm of bifurcationNEC
Peripheral vascular dis	7A1B700	Endovasstenting for aorto-uniiliac aneurysm
Peripheral vascular dis	7A1B800	Endovasc insert stent infrarenal abdo aortic aneurysm
Peripheral vascular dis	7A1B900	Endovasc insertion stent for suprarenal aortic aneurysm

Peripheral vascular dis	7A1BA00	Endovasinsertion of stent for thoracic aortic aneurysm
Peripheral vascular dis	7A1BC00	Endovas insert stent for aortic aneurysm of bifurcatio NEC
Peripheral vascular dis	7A1BD00	Endovasinsertion of stent for aorto-uniiliac aneurysm
Peripheral vascular dis	7A1Bz00	Transluminal operations on aneurysmal segment of aorta
Peripheral vascular dis	7A1C.00	Translum insert stent graft for aneurysmal segmt of aort
Peripheral vascular dis	7A1C000	Endovas ins stent graft for infrarenal abdom aortic aneury
Peripheral vascular dis	7A1C100	Endovas inse of stent graft for suprarenal aortic aneurysm
Peripheral vascular dis	7A1C200	Endov insertion of stent graft for thoracic aortic aneurysm
Peripheral vascular dis	7A1C500	Endovas insert of stent graft for aorto-uniiliac aneurysm
Peripheral vascular dis	7A1Cy00	OS translum ins stent graft for aneurysm segment of aorta
Peripheral vascular dis	7A1Cz00	Translum ins stent graft for aneurysmal segment of aorta
Peripheral vascular dis	7A41.00	Other bypass of iliac artery
Peripheral vascular dis	7A41.11	Other bypass of iliac artery by anastomosis
Peripheral vascular dis	7A41000	Emerg bypass iliac art by iliac/femoral art anastomosisNE
Peripheral vascular dis	7A41100	Bypass iliac artery by iliac/femoral artery anastomosis NE
Peripheral vascular dis	7A41200	Emerg bypass iliac artery by femoral/femoral art anast NE
Peripheral vascular dis	7A41211	Emergency femoro-femoral prosthetic cross over graft
Peripheral vascular dis	7A41300	Bypass iliac artery by femoral/femoral art anastomosis NE
Peripheral vascular dis	7A41311	Femoro-femoral prosthetic cross over graft
Peripheral vascular dis	7A41400	Emerg bypass comm iliac art by aorta/com iliac art anast
Peripheral vascular dis	7A41600	Emerg bypass leg artery by aorta/com fem art anastom
Peripheral vascular dis	7A41900	Bypass common iliac artery by aorta/com iliac art anast
Peripheral vascular dis	7A41B00	Bypass leg artery by aorta/com femoral art anastomosis
Peripheral vascular dis	7A41C00	Bypass leg artery by aorta/deep femoral art anastomosis
Peripheral vascular dis	7A41D00	Bypass iliac artery by iliac/iliac artery anastomosis NEC
Peripheral vascular dis	7A41F00	Ilio-femoral prosthetic cross over graft
Peripheral vascular dis	7A41y00	Other specified other bypass of iliac artery
Peripheral vascular dis	7A41z00	Other bypass of iliac artery NOS
Peripheral vascular dis	7A47.00	Other emergenc bypass of femoral artery/popliteal artery
Peripheral vascular dis	7A47.11	Other emerg bypass femoral or popliteal art by anastom
Peripheral vascular dis	7A47.12	Other emergency bypass of common femoral artery
Peripheral vascular dis	7A47.13	Other emergency bypass of deep femoral artery
Peripheral vascular dis	7A47.14	Other emergency bypass of popliteal artery
Peripheral vascular dis	7A47.15	Other emergency bypass of superficial femoral artery
Peripheral vascular dis	7A47.16	Other emergency bypass of femoral artery
Peripheral vascular dis	7A47000	Emerg bypass femoral art by fem/pop art anast c prosth
Peripheral vascular dis	7A47100	Emerg bypass popliteal art by pop/pop art anast c prosth
Peripheral vascular dis	7A47200	Emerg bypass femoral art by fem/pop a anast c vein graft
Peripheral vascular dis	7A47300	Emerg bypass pop art by pop/pop art anast c vein graft
Peripheral vascular dis	7A47400	Emerg bypass femoral art by fem/tib art anast c prosth N
Peripheral vascular dis	7A47600	Emerg bypass femoral art by fem/tib a anast c vein graft N
Peripheral vascular dis	7A47700	Emerg bypass pop art by pop/tib art anast c vein graft N
Peripheral vascular dis	7A47800	Emerg bypass femoral art by fem/peron art anast c prosth
Peripheral vascular dis	7A47B00	Emerg bypass pop art by pop/peron art anast c vein graft

Peripheral vascular dis	7A47C00	Emerg bypass femoral artery by fem/fem art anastomosis
Peripheral vascular dis	7A47D00	Emerg bypass popliteal artery by pop/fem art anastomos
Peripheral vascular dis	7A47y00	Other emergency bypass of femoral or popliteal artery OS
Peripheral vascular dis	7A47z00	Other emergency bypass of femoral or popliteal artery
Peripheral vascular dis	7A48.00	Other bypass of femoral artery or popliteal artery
Peripheral vascular dis	7A48.11	Other bypass of femoral or popliteal artery by anastomos
Peripheral vascular dis	7A48.12	Other bypass of common femoral artery
Peripheral vascular dis	7A48.14	Other bypass of femoral artery
Peripheral vascular dis	7A48.15	Other bypass of popliteal artery
Peripheral vascular dis	7A48.16	Other bypass of superficial femoral artery
Peripheral vascular dis	7A48000	Bypass femoral artery by fem/pop art anast c prosthesis
Peripheral vascular dis	7A48100	Bypass popliteal artery by pop/pop a anast c prosthesis N
Peripheral vascular dis	7A48200	Bypass femoral artery by fem/pop art anast c vein graft N
Peripheral vascular dis	7A48300	Bypass popliteal artery by pop/pop a anast c vein graft N
Peripheral vascular dis	7A48400	Bypass femoral artery by fem/tib art anast c prosthesis N
Peripheral vascular dis	7A48500	Bypass popliteal artery by pop/tib a anast c prosthesis N
Peripheral vascular dis	7A48600	Bypass femoral artery by fem/tib art anast c vein graft NE
Peripheral vascular dis	7A48700	Bypass popliteal artery by pop/tib a anast c vein graft NEC
Peripheral vascular dis	7A48800	Bypass femoral artery by fem/peron a anast c prosthesis
Peripheral vascular dis	7A48900	Bypass popliteal artery by pop/peron art anast c prosth N
Peripheral vascular dis	7A48A00	Bypass femoral artery by fem/peron a anast c vein graft N
Peripheral vascular dis	7A48B00	Bypass popliteal art by pop/peron art anast c vein graft N
Peripheral vascular dis	7A48C00	Bypass femoral artery by femoral/femoral art anastomos
Peripheral vascular dis	7A48D00	Bypass popliteal artery by pop/fem artery anastomosis N
Peripheral vascular dis	7A48E00	Femoro-femoral prosthetic cross over graft
Peripheral vascular dis	7A48y00	Other bypass of femoral artery or popliteal artery OS
Peripheral vascular dis	7A48z00	Other bypass of femoral artery or popliteal artery NOS
Peripheral vascular dis	9m1..00	Peripheral vascular dis monitoring invitation
Peripheral vascular dis	9m10.00	Peripheral vascular dis monitoring first letter
Peripheral vascular dis	9m11.00	Peripheral vascular dis monitoring second letter
Peripheral vascular dis	9m12.00	Peripheral vascular dis monitoring third letter
Peripheral vascular dis	A3A0.00	Gas gangrene
Peripheral vascular dis	A3A0000	Gas gangrene caused by clostridium histolyticum
Peripheral vascular dis	A3A0100	Gas gangrene caused by clostridium oedematiens
Peripheral vascular dis	A3A0200	Gas gangrene caused by clostridium perfringens
Peripheral vascular dis	A3A0500	Gas gangrene
Peripheral vascular dis	A3A0B00	Gas gangrene-hand
Peripheral vascular dis	A3A0E00	Gas gangrene-leg
Peripheral vascular dis	A3A0F00	Gas gangrene-foot
Peripheral vascular dis	G71..00	Aortic aneurysm
Peripheral vascular dis	G710.00	Dissecting aortic aneurysm
Peripheral vascular dis	G711.00	Thoracic aortic aneurysm which has ruptured
Peripheral vascular dis	G711.11	Ruptured thoracic aortic aneurysm
Peripheral vascular dis	G712.00	Thoracic aortic aneurysm without mention of rupture

Peripheral vascular dis	G713.00	Abdominal aortic aneurysm which has ruptured
Peripheral vascular dis	G713.11	Ruptured abdominal aortic aneurysm
Peripheral vascular dis	G713000	Ruptured suprarenal aortic aneurysm
Peripheral vascular dis	G714.00	Abdominal aortic aneurysm without mention of rupture
Peripheral vascular dis	G714.11	AAA - Abdominal aortic aneurysm without rupture
Peripheral vascular dis	G714000	Juxtarenal aortic aneurysm
Peripheral vascular dis	G714100	Inflammatory abdominal aortic aneurysm
Peripheral vascular dis	G714200	Infrarenal abdominal aortic aneurysm
Peripheral vascular dis	G714300	Aneurysm of suprarenal aorta
Peripheral vascular dis	G715.00	Ruptured aortic aneurysm NOS
Peripheral vascular dis	G715000	Thoracoabdominal aortic aneurysm, ruptured
Peripheral vascular dis	G716.00	Aortic aneurysm without mention of rupture NOS
Peripheral vascular dis	G716000	Thoracoabdominal aortic aneurysm, without rupture
Peripheral vascular dis	G717.00	Aortic aneurysm - syphilitic
Peripheral vascular dis	G718.00	Leaking abdominal aortic aneurysm
Peripheral vascular dis	G71z.00	Aortic aneurysm NOS
Peripheral vascular dis	G73..00	Other peripheral vascular dis
Peripheral vascular dis	G73..11	Peripheral ischaemic vascular dis
Peripheral vascular dis	G73..12	Ischaemia of legs
Peripheral vascular dis	G73..13	Peripheral ischaemia
Peripheral vascular dis	G732.00	Peripheral gangrene
Peripheral vascular dis	G732000	Gangrene of toe
Peripheral vascular dis	G732100	Gangrene of foot
Peripheral vascular dis	G732200	Gangrene of finger
Peripheral vascular dis	G732300	Gangrene of thumb
Peripheral vascular dis	G732400	Gangrene of hand
Peripheral vascular dis	G733.00	Ischaemic foot
Peripheral vascular dis	G734.00	Peripheral arterial dis
Peripheral vascular dis	G73y.00	Other specified peripheral vascular dis
Peripheral vascular dis	G73y100	Peripheral angiopathic dis EC NOS
Peripheral vascular dis	G73yz00	Other specified peripheral vascular dis NOS
Peripheral vascular dis	G73z.00	Peripheral vascular dis NOS
Peripheral vascular dis	G73z000	Intermittent claudication
Peripheral vascular dis	G73z011	Claudication
Peripheral vascular dis	G73z012	Vascular claudication
Peripheral vascular dis	G73zz00	Peripheral vascular dis NOS
Peripheral vascular dis	G76A.00	Arterial insufficiency
Peripheral vascular dis	Gyu7100	[X]Aortic aneurysm of unspecified site, ruptured
Peripheral vascular dis	Gyu7200	[X]Aortic aneurysm of unspecified site, nonruptured
Peripheral vascular dis	Gyu7400	[X]Other specified peripheral vascular diss
Peripheral vascular dis	Gyu7800	[X]Aneurysm of aorta in diss classified elsewhere
Peripheral vascular dis	R054.00	[D]Gangrene
Peripheral vascular dis	R054000	[D]Gangrene, spreading cutaneous
Peripheral vascular dis	R054200	[D]Gangrene of toe in diabetic

Peripheral vascular dis	R054300	[D]Widespread diabetic foot gangrene
Peripheral vascular dis	R054z00	[D]Gangrene NOS
